# Safety and efficacy of multimatrix mesalamine in paediatric patients with mild-to-moderate ulcerative colitis: a phase 3, randomised, double-blind study

**DOI:** 10.1016/j.eclinm.2023.102232

**Published:** 2023-10-06

**Authors:** Nicholas Michael Croft, Bartosz Korczowski, Jarosław Kierkuś, Beatriz Caballero, Manoj Kumar Thakur

**Affiliations:** a Faculty of Medicine, Blizard Institute, Queen Mary University of London, London, UK; b Paediatric Gastroenterology, Royal London Children’s Hospital, Barts Health NHS Trust, London, UK; c Department of Pediatrics and Pediatric Gastroenterology, College of Medical Sciences, University of Rzeszów, Rzeszów, Poland; d Department of Gastroenterology, Hepatology and Feeding Disorders, Children’s Memorial Health Institute, Warsaw, Poland; e Takeda Clinical Science, Zurich, Switzerland; f Takeda Development Center Americas, Inc., Lexington, MA, USA

**Keywords:** Efficacy, Mild-to-moderate ulcerative colitis, Multimatrix mesalamine, Paediatric, Phase 3 study, Safety, Trial

## Abstract

**Background:**

Previous studies have demonstrated the tolerability and efficacy of multimatrix mesalamine in inducing and maintaining remission in adults with mild-to-moderate ulcerative colitis (UC). We evaluated the safety and efficacy of low-dose and high-dose once-daily multimatrix mesalamine in children and adolescents with mild-to-moderate UC or those in remission.

**Methods:**

This prospective, randomised, parallel-group, phase 3 study (8-week double-blind acute [DBA] phase; 26-week double-blind maintenance [DBM] phase; and an additional 8-week, open-label acute [OLA] phase) was conducted in 33 sites across North America, Europe, and the Middle East between December 12, 2014, and November 28, 2018. Eligible patients aged 5–17 years and weighing 18–90 kg were randomised 1:1 to either low (900–2400 mg) or high (1800–4800 mg) oral doses of multimatrix mesalamine once daily, stratified by body weight. Interactive response technology was used for randomisation. The primary efficacy outcome was to estimate the clinical response of multimatrix mesalamine (two doses) in different weight groups. Efficacy and safety analyses were conducted in the safety analysis set (Clinicaltrials.gov: NCT02093663; Study completed).

**Findings:**

Overall, 107 patients were randomised into the DBA (n = 54) or DBM phase (n = 88; directly or after completing the double-blind or OLA phases); the overall safety analysis set included 105 patients. In the DBA phase, the high-dose group (n = 17; 65.4%) achieved a higher clinical response rate than the low-dose (n = 10; 37.0%) group; difference 28.3% (95% CI: 2.5–54.2; p = 0.039), odds ratio (OR) 3.21 (95% CI: 1.04–9.88). In the DBM phase at Week 26, similar proportions of patients maintained clinical response in the low-dose (n = 23; 54.8%) and high-dose (n = 24; 53.3%) groups: OR 0.99 (0.42–2.34); p = 0.981. Overall, 246 treatment-emergent adverse events (TEAEs) were reported in 73 patients (69.5%); 23 TEAEs in 14 patients (13.3%) were considered related to the study drug. No treatment-related deaths were reported.

**Interpretation:**

Our findings suggested that the benefit-risk ratio of once-daily multimatrix mesalamine in paediatric patients was favourable and comparable with that reported in adults with mild-to-moderate UC.

**Funding:**

Shire Development LLC, a Takeda company.

**Research in context**

**Evidence before this study**

Multimatrix mesalamine, a once-daily, high-strength, oral formulation of mesalamine, is efficacious in induction and maintenance of ulcerative colitis (UC) remission in adult patients with mild-to-moderate disease. However, to date, no randomised controlled trials (RCTs) have been conducted on the safety and efficacy of once-daily multimatrix mesalamine in children and adolescents with UC. PubMed was searched for articles containing the terms “multimatrix mesalamine”, “ulcerative colitis”, “randomized trial”, and “adolescent OR children OR pediatric”. The date range used was from database inception to August 1, 2023.

**Added value of this study**

To our knowledge, this was the first randomised study of once-daily mesalamine in children. It has demonstrated the efficacy of once-daily oral dosing of multimatrix mesalamine in inducing and maintaining clinical response in paediatric patients with mild-to-moderate UC, or those in remission.

**Implications of all the available evidence**

The results from this study show that multimatrix mesalamine offers a favourable benefit-risk profile in paediatric patients with mild-to-moderate UC and provides evidence to support once-daily dosing in children.

## Introduction

Ulcerative colitis (UC) is a chronic inflammatory disease of the colon and rectum whose major clinical feature is bloody diarrhoea.^1^ The characteristic disease course of UC is marked by remissions and exacerbations over a number of years.^2^ Paediatric-onset UC constitutes approximately 15%–20% of all UC cases, with incidence ranging from one to four of 100,000 individuals per year in North America and Europe.^3^^,^^4^ Most children with UC are diagnosed in late childhood and adolescence, although it can also occur earlier in life.^1^ The disease course tends to be more severe in children than in adults, with more extensive localisation.^5^ Children with UC require hospitalisation for acute severe exacerbation (25%–30% over 3–4 years) more often than adults, and they more often undergo colectomy for medically refractory disease.^6^, ^7^, ^8^, ^9^, ^10^ In children, a colectomy rate of 30%–40% is seen at 10 years compared with that of 20% in adults with UC.^4^^,^^6^

Mesalamine (5-aminosalicylic acid [5-ASA]), a standard first-line treatment for mild-to-moderate UC,^11^^,^^12^ is well tolerated and has a comparable safety profile in children and adults.^10^ Oral mesalamine preparations in children are typically administered in two to three divided doses daily.^13^^,^^14^ Multimatrix mesalamine is a once-daily, high-strength, oral formulation of 5-ASA that is effective for both the induction and maintenance of UC remission in adult patients with mild-to-moderate UC.^15^, ^16^, ^17^ Induction treatment with multimatrix mesalamine is well tolerated with similar rates of treatment-emergent adverse events (TEAEs) as observed among patients treated with a placebo; moreover, maintenance with multimatrix mesalamine was found to be well tolerated in a pooled analysis of long-term safety.^17^^,^^18^ Currently, limited data are available on the safety and efficacy of once-daily multimatrix mesalamine in children and adolescents with UC.

This study aimed to evaluate the safety and efficacy of multimatrix mesalamine in children and adolescents with mild-to-moderate UC or those in remission, and to select an appropriate once-daily dosage in the paediatric population across a range of weight groups.

## Methods

### Study design and participants

**Fig. 1:**
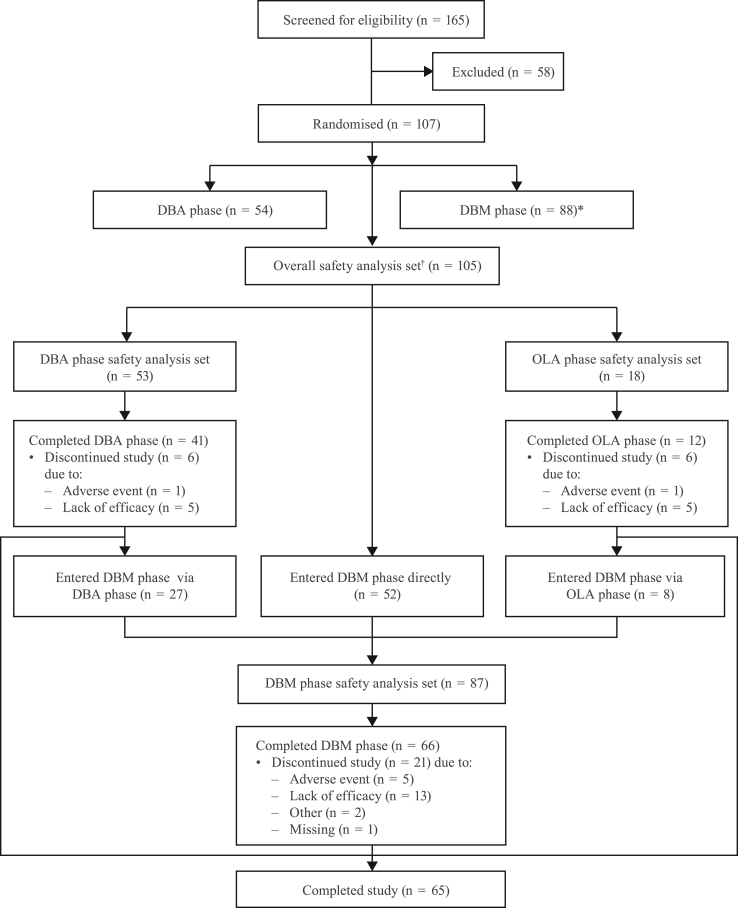
**Patient disposition flow diagram.** DBA = double-blind acute; DBM = double-blind maintenance; OLA = open-label acute; PGA = Physician’s Global Assessment; UC-DAI = Ulcerative Colitis Disease Activity Index. ∗Patients with a clinical response (i.e., partial UC-DAI ≤1 [defined as rectal bleeding = 0, stool frequency ≤1, and PGA = 0]) after completion of treatment in either the DBA or OLA phase were eligible for re-randomisation into the DBM phase provided they still met all baseline visit (Visit 2) inclusion and exclusion criteria (where reassessed). ^†^The overall safety analysis set consisted of randomised patients who had taken at least one dose of investigational product.

This prospective, multicentre, randomised, double-blind, parallel-group, phase 3 study was a Pediatric Research Equity Act post-approval commitment with the United States (US) Food and Drug Administration (FDA) intended to estimate clinical response for two doses (low and high) across a range of weight groups. This study was conducted in 33 sites across North America, Europe, and the Middle East and screened 165 patients for eligibility (Fig. 1). The inclusion and exclusion criteria used are detailed in the Supplementary Methods and Supplementary Table S1.

**Fig. 2:**
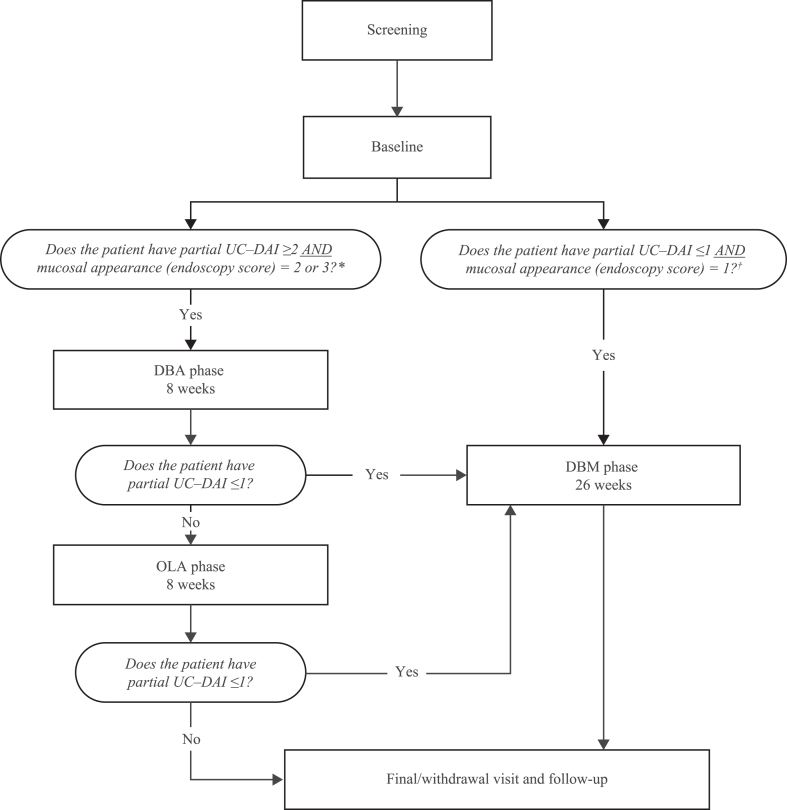
**Study design.** DBA = double-blind acute; DBM = double-blind maintenance; OLA = open-label acute; PGA = Physician’s Global Assessment; UC-DAI = Ulcerative Colitis Disease Activity Index. ∗Patients with partial UC-DAI ≥2 (with a combined rectal bleeding ***and*** stool frequency score of ≥1 ***and*** PGA = 1 or 2) ***and*** with mucosal appearance (endoscopy score) = 2 or 3. ^†^Patients with partial UC-DAI ≤1 (with rectal bleeding = 0 ***and*** stool frequency ≤1 ***and*** PGA = 0) ***and*** with mucosal appearance (endoscopy score) = 0 or 1.

The study included a screening period of 3–21 days, an 8-week double-blind acute (DBA) phase, and a 26-week double-blind maintenance (DBM) phase (Fig. 2). There was an additional 8-week, open-label acute (OLA) phase for patients who did not achieve a clinical response, who were discontinued from the DBA phase and met certain criteria, or who were discontinued from the DBA phase after ≥2 weeks and, in the investigator’s opinion, did not benefit from treatment in the DBA phase. Clinical response was defined as partial Ulcerative Colitis Disease Activity Index (UC-DAI) ≤1 (with rectal bleeding = 0, stool frequency ≤1, and Physician’s Global Assessment [PGA] = 0) (Supplementary Table S2).

This study was conducted in accordance with the International Council for Harmonisation–Good Clinical Practice guidelines, Declaration of Helsinki, and the local ethical and legal requirements. Each patient or the patient’s legally authorised representative, as applicable, provided written informed consent.

### Randomisation and masking

In the DBA phase, patients were randomised in a 1:1 ratio into low (900–2400 mg) or high (1800–4800 mg) once-daily doses of multimatrix mesalamine. Eligible patients from the DBA or OLA phase were re-randomised into the DBM phase. Randomisation was stratified by body weight group. Patients and investigators were blinded to the study treatment. The allocation sequence was generated by interactive response technology, and the blinding part was ensured by keeping both doses of multimatrix mesalamine in identical blister packs.

### Procedures

For the purpose of this study, two paediatric formulations (300- and 600-mg tablets) were developed, which were smaller in size and of lower dosages than the commercially available formulation (1.2 g). Multimatrix mesalamine was administered based on the patient’s body weight: 900 or 1800 mg once daily for patients weighing 18 to ≤23 kg, 1200 or 2400 mg once daily for patients weighing >23 to ≤35 kg, 1800 or 3600 mg once daily for patients weighing >35 to ≤50 kg, and 2400 or 4800 mg once daily for patients weighing >50 to ≤90 kg. In the OLA phase, patients were treated with a high-dose multimatrix mesalamine as appropriate for their weight group.

After the screening visit (Visit 1), patients who were eligible proceeded to the baseline visit (Visit 2). At Visit 2, patients with partial UC-DAI ≥2 (with a combined rectal bleeding and stool frequency score of ≥1 and PGA = 1 or 2) and with mucosal appearance (endoscopy score) = 2 or 3 were eligible to enter the DBA phase, and patients with partial UC-DAI ≤1 (with rectal bleeding = 0 and stool frequency ≤1 and PGA = 0) and with mucosal appearance (endoscopy score) = 0 or 1 were eligible to enter the DBM phase. Patients with a clinical response after treatment completion in either the DBA or OLA phase were eligible to enter the DBM phase based on their partial UC-DAI scores (i.e., without additional endoscopy). Patients without a clinical response after treatment completion in both the DBA and OLA phases were not eligible to enter the DBM phase and were discontinued.

Patients were instructed to bring their unused study medication and empty/used study medication packaging at every visit. Treatment compliance was assessed at each visit at the container/packaging level for unused study medication that was contained within the original tamper-evident sealed container or at the individual count level for opened containers/packaging; compliance was calculated for all study visits after the baseline visit except at follow-up. Patients were considered compliant if they had taken 80%–120% of their study medication.

### Outcomes

The different outcome measures used in this study, such as PGA, Pediatric Ulcerative Colitis Activity Index (PUCAI), and UC-DAI, are detailed in Supplementary Table S2. The primary outcomes were the proportion of patients with a clinical response at Week 8 for the DBA phase and the proportion of patients who had maintained a clinical response at Week 26 for the DBM phase. Secondary outcomes included the proportion of patients with a clinical and endoscopic response at Week 8 (DBA phase) and Week 26 (DBM phase); the change in Daily Ulcerative Colitis Scale (DUCS) score^19^ for children and caregivers from baseline to Week 8 (DBA phase) and from Week 0 to Week 26 (DBM phase); the proportion of patients with improvement (change of ≥20 points) in PUCAI score^20^ from baseline to Week 8 (DBA phase); and the proportion of patients in remission (PUCAI <10) at Week 26 (DBM phase). Endoscopic activity was assessed using the Mayo endoscopic score. Clinical and endoscopic response was defined as UC-DAI ≤2 with rectal bleeding = 0, stool frequency ≤1, and PGA = 0 and with mucosal healing (endoscopy score ≤1) based on central and local reading; in addition, in the DBA phase, there must have been a ≥1-point reduction in endoscopy score from baseline. The DUCS is a content-valid patient/observer-reported outcome instrument for capturing the daily signs and symptoms of UC in paediatric patients aged 5–17 years with mild-to-moderate UC in a clinical trial setting.^19^

Safety was assessed by reviewing adverse events, clinical laboratory tests, physical examinations, vital signs, medical and medication history, and stool characteristics. Adverse events were coded using the Medical Dictionary for Regulatory Activities (MedDRA). TEAEs were classified according to their severity (mild, moderate, or severe). Serious TEAEs were defined as those that resulted in death, congenital anomalies or birth defects, persistent or significant disability, inpatient hospitalisation, or prolonged hospitalisation, or those considered life-threatening or important medical events. The number and percentage of patients with any TEAEs, serious TEAEs, TEAEs related to the study drug, TEAEs leading to discontinuation of the study drug, and TEAEs leading to death were summarised.

### Statistical analysis

This study was an estimation study with no formal hypothesis testing; therefore, the study was not powered to detect differences between treatment groups. More than 100 patients were to be screened and up to 80 patients were to be enrolled in the DBA phase of the study. After agreement with the US FDA, the sample size for the DBA phase was reduced to 53 patients owing to difficulties in recruitment. p-values are presented as descriptive statistics only. The overall safety analysis set consisted of randomised patients who had taken ≥1 dose of multimatrix mesalamine. An independent data monitoring committee reviewed the safety data generated during the study.

In the DBA phase, the primary efficacy outcome was compared between treatment groups using an uncorrected chi-square test on the DBA safety analysis set. In the DBM phase, the primary efficacy outcome was compared between the treatment groups using a Cochran–Mantel–Haenszel test stratified for three levels of Week 8 responder status (entered DBM phase directly, responder at Week 8 of the DBA phase, or responder at Week 8 of the OLA phase) in the DBM phase safety analysis set. Analyses were performed using SAS version 9.2 or higher (SAS Institute Inc., Cary, NC, USA). The detailed statistical analysis plan used in this study is presented in the Supplementary Material. This study is registered with ClinicalTrials.gov (NCT02093663).

### Role of the funding source

The funder of the study had a role in the study design, data collection, data analysis/interpretation, clinical study report writing, and the decision to submit the manuscript. All authors had full access to all the data in the study and accept responsibility for the decision to submit for publication.

## Results

Between December 12, 2014, and November 28, 2018, a total of 165 patients were screened, of whom 107 met the enrolment criteria and were randomised into the DBA (n = 54) or DBM phase (n = 88), either directly or after completing treatment in the DBA or OLA phase (Fig. 1). The safety analysis set had 105 patients; the DBA phase, OLA phase, and DBM phase safety analysis sets had 53, 18, and 87 patients, respectively. In the DBM phase safety analysis set, 52 entered directly, 27 entered through the DBA phase, and eight entered through the OLA phase (Fig. 1). Overall, 65 patients (60.7%) completed the study. Among the 42 patients (39.3%) who did not complete the study, 35 discontinued from their last study phase owing to lack of efficacy (21.5%), adverse events (6.5%), or other reason (3.7%); four (3.7%) were not enrolled in the DBM phase; two (1.9%) did not continue in the study; and one (0.9%) did not indicate a reason for withdrawal (Fig. 1, Supplementary Table S3).

The patient populations in the three study phases were not mutually exclusive; as such, direct comparison of the baseline demographic characteristics is not possible, and data interpretation needs to be performed with caution. The median age of patients was 15.0 years (range: 5–17 years; Table 1). The safety analysis set included 10 patients (9.5%) aged 5–10 years and 95 patients (90.5%) aged 11–17 years. There were similar proportions of male (49.5%) and female (50.5%) patients. Patient demographics by phase were similar to those observed overall, with the exceptions of more male than female patients in the DBA (64.2% vs 35.8%) and OLA (72.2% vs 27.8%) phases and of more female than male patients in the DBM phase (55.2% vs 44.8%; Table 1). For the DBA and DBM phases, no notable differences were seen between patients receiving low-dose or high-dose multimatrix mesalamine. Mean (standard deviation [SD]) time since diagnosis was 16.6 (31.5) months, and 58 patients (55.2%) were not newly diagnosed. The mean (SD) number of acute UC episodes in the past year and since diagnosis was 0.9 (0.9) and 1.6 (1.3), respectively. The extent of disease was classified only for patients who were not newly diagnosed; it was noted as left-sided in 22 patients (21.0%) and involved the transverse colon in 14 patients (13.3%); 21 patients (20.0%) had pancolitis. Patient characteristics by phase were similar to those observed overall, except that most patients in the DBA (77.4%) and OLA (77.8%) phases were newly diagnosed (Table 1). Overall, 87 (82.9%) patients received prior medications, and 102 (97.1%) reported taking concomitant medications. Aminosalicylic acid and similar agents were the most common type of medications taken prior to and during the study (Table 1).

**Table 1:** Demographic and baseline clinical characteristics: overall safety analysis set (N = 105).^a^

	DBA phase			DBM phase			OLA phase	Total (N = 105)
	Low-dose MMX (n = 27)	High-dose MMX (n = 26)	Overall (n = 53)	Low-dose MMX (n = 42)	High-dose MMX (n = 45)	Overall (n = 87)	High-dose MMX (n = 18)	
Age, years			–			–	–	–
Mean (SD)	13.6 (2.2)	14.4 (2.3)	14.0 (2.3)	14.3 (2.2)	14.2 (2.88)	14.2 (2.6)	13.4 (2.3)	14.1 (2.6)
Median (IQR)	14.0 (12–15)	15.0 (13–16)	14.0 (13–15)	15.0 (13–16)	15.0 (13–16)	15.0 (13–16)	13.5 (13–15)	15.0 (13–16)
Age category			–			–	–	–
5–10 years	2 (7.4%)	2 (7.7%)	4 (7.5%)	3 (7.1%)	5 (11.1%)	8 (9.2%)	1 (5.6%)	10 (9.5%)
11–17 years	25 (92.6%)	24 (92.3%)	49 (92.5%)	39 (92.9%)	40 (88.9%)	79 (90.8%)	17 (94.4%)	95 (90.5%)
Sex			–			–	–	–
Male	16 (59.3%)	18 (69.2%)	34 (64.2%)	19 (45.2%)	20 (44.4%)	39 (44.8%)	13 (72.2%)	52 (49.5%)
Female	11 (40.7%)	8 (30.8%)	19 (35.8%)	23 (54.8%)	25 (55.6%)	48 (55.2%)	5 (27.8%)	53 (50.5%)
Race^b^			–			–	–	–
White	25 (92.6%)	24 (92.3%)	49 (92.5%)	40 (95.2%)	44 (97.8%)	84 (96.6%)	17 (94.4%)	101 (96.2%)
Black/African American	1 (3.7%)	0	1 (1.9%)	1 (2.4%)	0	1 (1.1%)	0	1 (1.0%)
Asian	1 (3.7%)	2 (7.7%)	3 (5.7%)	1 (2.4%)	1 (2.2%)	2 (2.3%)	1 (5.6%)	3 (2.9%)
Ethnicity			–			–	–	–
Hispanic or Latino	0	0	0	1 (2.4%)	0	1 (1.1%)	0	1 (1.0%)
Weight (kg)								
Mean (SD)	52.8 (12.9)	52.6 (13.1)	52.7 (12.9)	54.4 (11.9)	53.7 (14.8)	54.0 (13.4)	54.3 (13.2)	53.2 (13.8)
Median (IQR)	53.1 (41.0–65.2)	53.4 (42.8–63.0)	53.1 (42.8–63.0)	54.90 (45.4–64.4)	53.0 (45.6–64.2)	54.7 (45.4–64.4)	53.6 (48.7–63.0)	53.5 (44–64)
Weight group (kg)								
18 to ≤23	0	0	0	1 (2.4%)	2 (4.4%)	3 (3.4%)	0	3 (2.9%)
>23 to ≤35	4 (14.8%)	3 (11.5%)	7 (13.2%)	2 (4.8%)	2 (4.4%)	4 (4.6%)	2 (11.1%)	9 (8.6%)
>35 to ≤50	7 (25.9%)	7 (26.9%)	14 (26.4%)	11 (26.2%)	13 (28.9%)	24 (27.6%)	4 (22.2%)	28 (26.7%)
>50 to ≤90	16 (59.3%)	16 (61.5%)	32 (60.4%)	28 (66.7%)	28 (62.2%)	56 (64.4%)	12 (66.7%)	65 (61.9%)
Height (cm)								
Mean (SD)	161.6 (12.35)	164.4 (13.73)	163.0 (13.00)	162.7 (11.23)	161.0 (15.82)	161.8 (13.74)	165.4 (14.84)	161.7 (14.01)
Median (IQR)	165.0 (155.0–169.7)	164.5 (156.2–175.4)	165.0 (156.2–173.0)	163.4 (156–170.2)	164.0 (157–173)	164.0 (156–171.1)	167.4 (161–176.4)	164.0 (156.2–172.0)
BMI, kg/m^2^			–			–	–	–
Mean (SD)	19.9 (2.9)	19.2 (3.0)	19.6 (2.9)	20.3 (3.0)	20.4 (4.2)	20.4 (3.7)	19.5 (2.2)	20.0 (3.6)
Median (IQR)	20.7 (17.5–21.9)	19.0 (17.1–21.0)	19.2 (17.1–21.6)	20.2 (18.6–21.7)	20.1 (17.7–22.2)	20.1 (18–22)	19.7 (17.4–20.9)	19.7 (17.5–21.9)
Time since diagnosis^c^ (months)	1.7 (4.7)	12.7 (38.3)	7.1 (27.4)	20.0 (32.3)	16.6 (32.1)	18.2 (32.0)	17.1 (45.7)	16.6 (31.5)
Method of diagnosis^d^								
Sigmoidoscopy	5 (18.5%)	7 (26.9%)	12 (22.6%)	6 (14.3%)	8 (17.8%)	14 (16.1%)	3 (16.7%)	19 (18.1%)
Colonoscopy	27 (100.0%)	22 (84.6%)	49 (92.5%)	40 (95.2%)	41 (91.1%)	81 (93.1%)	15 (83.3%)	97 (92.4%)
Compatible histology	27 (100.0%)	26 (100.0%)	53 (100.0%)	42 (100.0%)	45 (100.0%)	87 (100.0%)	18 (100.0%)	105 (100.0%)
Number of newly diagnosed patients	23 (85.2%)	18 (69.2%)	41 (77.4%)	15 (35.7%)	18 (40.0%)	33 (37.9%)	14 (77.8%)	47 (44.8%)
Number of acute UC episodes in the past year^e^	1.8 (1.0) (n = 4)	1.1 (0.6) (n = 8)	1.3 (0.8) (n = 12)	1.0 (1.0) (n = 26)	0.9 (0.8) (n = 27)	0.9 (0.9) (n = 53)	1.0 (0.8) (n = 4)	0.9 (0.9)
Number of acute UC episodes since diagnosis^e^	2.7 (2.1) (n = 3)	1.8 (1.0) (n = 8)	2.0 (1.3) (n = 11)	1.6 (1.3) (n = 26)	1.4 (1.2) (n = 27)	1.5 (1.2) (n = 53)	2.7 (1.2) (n = 3)	1.6 (1.3)
Classification of extent of disease^e^	–	–	–	–	–	–	–	–
Left-sided	3 (11.1%)	3 (11.5%)	6 (11.3%)	9 (21.4%)	12 (26.7%)	21 (24.1%)	2 (11.1%)	22 (21.0%)
Transverse colon involvement	0	1 (3.8%)	1 (1.9%)	9 (21.4%)	4 (8.9%)	13 (14.9%)	1 (5.6%)	14 (13.3%)
Pancolitis	1 (3.7%)	4 (15.4%)	5 (9.4%)	8 (19.0%)	11 (24.4%)	19 (21.8%)	1 (5.6%)	21 (20.0%)
Rectal involvement	3 (11.1%)	5 (19.2%)	8 (15.1%)	22 (52.4%)	19 (42.2%)	41 (47.1%)	3 (16.7%)	44 (41.9%)
Extraintestinal manifestations	0	3 (11.5%)	3 (5.7%)	6 (14.3%)	5 (11.1%)	11 (12.6%)	2 (11.1%)	12 (11.4%)
History of significant GI surgery	0	0	0	0	1 (2.2%)	1 (1.1%)	0	1 (1.0%)
Full extent of the disease (cm)^e^^,^^f^	–	–	–	–	–	–	–	–
≤15	2 (7.4)	1 (3.8)	3 (5.7)	5 (11.9)	6 (13.3)	11 (12.6)	1 (5.6)	12 (11.4)
>15	2 (7.4)	7 (26.9)	9 (17.0)	21 (50.0)	21 (46.7)	42 (48.3)	3 (16.7)	45 (42.9)
Baseline disease activity								
Partial UC-DAI score	4.0 (1.5)	3.9 (1.7) (n = 25)	–	0.1 (0.3) (n = 41)	0.2 (0.4) (n = 44)	–	2.8 (1.7) (n = 16)	–
Full UC-DAI score (central reading)	5.6 (1.7) (n = 25)	6.0 (1.9) (n = 23)	–	0.6 (0.7) (n = 26)	0.6 (0.6) (n = 29)	–	1.7 (−) (n = 1)	–
PUCAI scores	35.6 (14.2)	31.9 (14.1)	–	1.3 (3.3)	2.8 (5.0)	–	24.1 (19.4) (n = 17)	–
Mild (10–34)	10 (37.0%)	10 (38.5%)	–	3 (7.1%)	6 (13.3%)	–	7 (38.9%)	–
Moderate (35–64)	15 (55.6%)	14 (53.8%)	–	0	0	–	6 (33.3%)	–
Severe (≥65)	2 (7.4%)	0 (0%)	–	0	0	–	0	–
Remission (PUCAI score <10)	0	2 (7.7%)	–	39 (92.9%)	39 (86.7%)	–	4 (22.2%)	–
Any prior medication	22 (81.5%)	17 (65.4%)	39 (73.6%)	36 (85.7%)	38 (84.4%)	74 (85.1%)	–	87 (82.9%)
Aminosalicylic acid and similar agents	6 (22.2%)	6 (23.1%)	12 (22.6%)	25 (59.5%)	27 (60.0%)	52 (59.8%)	–	58 (55.2%)
Local corticosteroids	0	1 (3.8%)	1 (1.9%)	4 (9.5%)	3 (6.7%)	7 (8.0%)	–	7 (6.7%)
Local corticosteroids, oral	2 (7.4%)	1 (3.8%)	3 (5.7%)	0	2 (4.4%)	2 (2.3%)	–	4 (3.8%)
Any concomitant medication	27 (100.0%)	24 (92.3%)	51 (96.2%)	41 (97.6%)	43 (95.6%)	84 (96.6%)	18 (100.0%)	102 (97.1%)
Aminosalicylic acid and similar agents	18 (66.7%)	17 (65.4%)	35 (66.0%)	34 (81.0%)	37 (82.2%)	71 (81.6%)	12 (66.7%)	82 (78.1%)
Local corticosteroids	8 (29.6%)	8 (30.8%)	16 (30.2%)	8 (19.0%)	6 (13.3%)	14 (16.1%)	9 (50.0%)	24 (22.9%)
Local corticosteroids, oral	3 (11.1%)	1 (3.8%)	4 (7.5%)	1 (2.4%)	3 (6.7%)	4 (4.6%)	1 (5.6%)	7 (6.7%)

Data are n (%), median (IQR), mean (SD).

BMI = body mass index; DBA = double-blind acute; DBM = double-blind maintenance; GI = gastrointestinal; MMX = multimatrix mesalamine; OLA = open-label acute; SD = standard deviation; UC = ulcerative colitis.

a Patients who received one or more doses of multimatrix mesalamine.

b Percentages may not total to 100 due to rounding.

c Multiple methods may apply per participant. A minimum of two techniques must be selected and one of them must be compatible histology.

d Time since diagnosis (months) is calculated as (screening date − diagnosis date + 1)/30.

e Only for patients who were not newly diagnosed.

f Measured from anal margin (cm) at the time of the most recent endoscopy.

Baseline disease activity scores confirmed that most patients had mild-to-moderate UC and are presented in Table 1. Particularly, in the DBA phase, the mean (SD) partial UC-DAI score was 4.0 (1.5) in the low-dose group (n = 27) and 3.9 (1.7) in the high-dose group (n = 25). The mean (SD) partial UC-DAI score in the DBM phase was 0.1 (0.3) and 0.2 (0.4) in the low-dose (n = 41) and high-dose (n = 44) groups, respectively. In the OLA phase, the partial UC-DAI score was 2.8 (1.7) (n = 16). The mean (SD) full UC-DAI score based on central reading was 5.6 (1.7) in the low-dose group (n = 25) and 6.0 (1.9) in the high-dose group (n = 23) in the DBA phase; 0.6 (0.7) in the low-dose group (n = 26) and 0.6 (0.6) in the high-dose group (n = 29) in the DBM phase; and 1.7 (−) in the high-dose group (n = 1) in the OLA phase. In the DBA phase, no (0%) and two (7.7%) patients in the low-dose and high-dose groups, respectively, were in remission (PUCAI score <10). In the DBM phase, 39 (92.9%) patients in the low-dose group and 39 (86.7%) in the high-dose group were in remission. In the OLA phase, four (22.2%) patients were in remission.

In the DBA phase, the multimatrix mesalamine dose per kg for the low-dose and high-dose groups, respectively, were 35.6 and 77.4 mg for patients with baseline weight of >23 to ≤35 kg, 41.6 and 84.7 mg for patients weighing >35 to ≤50 kg, and 38.9 and 78.5 mg for patients weighing >50 to ≤90 kg (Table 2). In the DBM phase, the multimatrix mesalamine dose per kg for the low-dose and high-dose groups, respectively, was 39.1 and 89.3 mg for patients weighing 18 to ≤23 kg, 37.4 and 82.3 mg for patients weighing >23 to ≤35 kg, 40.7 and 83.3 mg for patients weighing >35 to ≤50 kg, and 39.3 and 76.6 mg for patients weighing >50 to ≤90 kg (Table 2).

**Table 2:** Multimatrix mesalamine dose per kg by baseline weight group.

Baseline weight group	DBA phase (n = 53)			DBM phase (n = 87)		
	n	Average weight (kg)	Dose per kg (mg)	n	Average weight (kg)	Dose per kg (mg)
18 to ≤23 kg^a^	–	–	–	–	–	–
Low dose	–	–	–	1	23.0	39.1
High dose	–	–	–	2	20.2	89.3
>23 to ≤35 kg	–	–	–	–	–	–
Low dose	4	33.8	35.6	2	32.1	37.4
High dose	3	31.0	77.4	2	29.2	82.3
>35 to ≤50 kg	–	–	–	–	–	–
Low dose	7	43.2	41.6	11	44.3	40.7
High dose	7	42.5	84.7	13	43.2	83.3
>50 to ≤90 kg	–	–	–	–	–	–
Low dose	16	61.7	38.9	28	61.1	39.3
High dose	16	61.1	78.5	28	62.7	76.6

DBA = double-blind acute; DBM = double-blind maintenance.

a There were no patients in the 18 to ≤23-kg weight group in the DBA phase.

During the DBA phase, most patients (52 [98.1%]) took 80%–120% of their study medication (26 patients [96.3%] in the low-dose group and 26 patients [100.0%] in the high-dose group); of these 52 patients, 20 (38.5%) and 32 (61.5%) were 5–13 and 14–17 years of age, respectively. During the OLA phase, 14 patients (77.8%) were 80%–120% compliant; of the 14 patients, eight (57.1%) were 5–13 years of age and six (42.9%) were 14–17 years of age. During the DBM phase, most patients (81 [93.1%]) were 80%–120% compliant (38 patients [90.5%] in the low-dose group and 43 patients [95.6%] in the high-dose group); of these 81 patients, 24 (29.6%) were 5–13 years of age and 57 (70.4%) were 14–17 years of age.

**Fig. 3:**
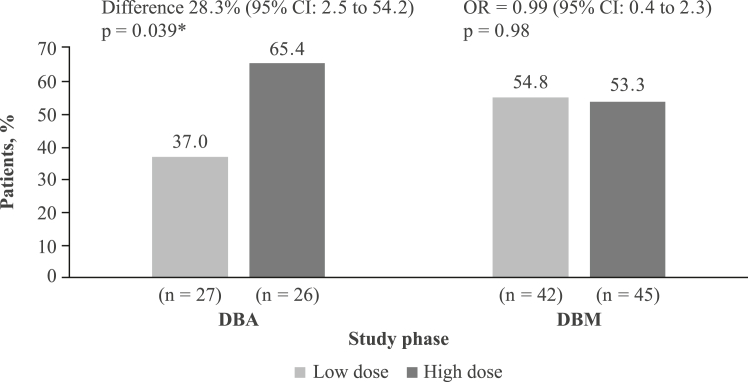
**Proportion of patients with clinical response to multimatrix mesalamine in the DBA (Week 8) and DBM (Week 26) phases (primary efficacy outcomes).** CI = confidence interval; DBA = double-blind acute; DBM = double-blind maintenance; OR = odds ratio. ∗Difference between the low-dose and high-dose multimatrix mesalamine treatment groups. Odds ratio was calculated between the high-dose and low-dose multimatrix mesalamine treatment groups.

In the DBA phase, a higher clinical response rate at Week 8 was observed among patients on high-dose (n = 17; 65.4%) vs low-dose (n = 10; 37.0%) multimatrix mesalamine (difference = 28.3% [95% CI: 2.5–54.2]; Fig. 3); the odds ratio (OR) was 3.21 (95% CI: 1.04–9.88 [p = 0.039]). In the DBM phase, similar proportions of patients maintained clinical response at Week 26 in the low-dose (n = 23; 54.8%) and high-dose (n = 24; 53.3%) groups (difference = −1.4% [95% CI: −22.4 to 19.5]); the OR was 0.99 (95% CI: 0.42–2.34; p = 0.98; Fig. 3). Sensitivity analyses were performed for both the DBA and DBM phases on the following parameters: modified clinical response, complete case analysis, and last observation carried forward. The results were similar to those from the primary efficacy outcome analysis.

**Fig. 4:**
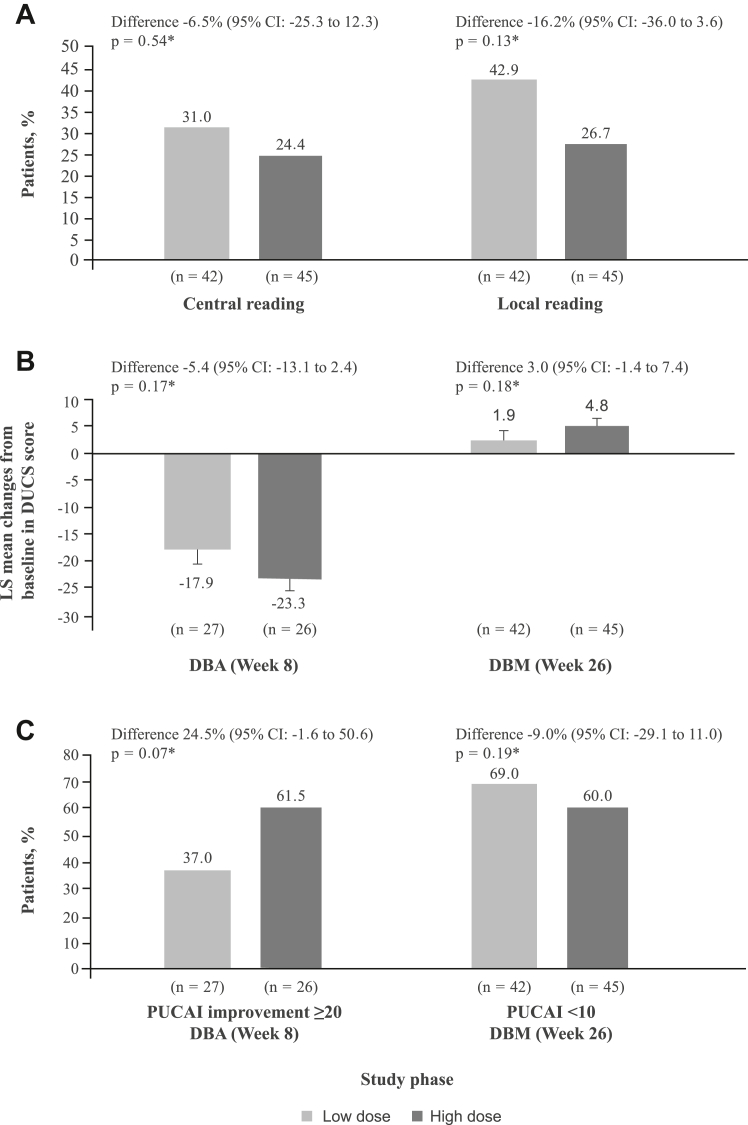
**Secondary efficacy outcomes: (A) Proportion of patients with clinical and endoscopic response**^**†**^**to multimatrix mesalamine at Week 26 in the DBM phase; (B) LS mean (SEM) change from baseline in DUCS score at Week 8 and Week 26∗; and (C) Proportion of patients with PUCAI improvement (Week 8) and remission (Week 26)∗.** CI = confidence interval; DBA = double-blind acute; DBM = double-blind maintenance; DUCS = Daily Ulcerative Colitis Scale; LS = least-squares; PUCAI = Pediatric Ulcerative Colitis Activity Index; SEM = standard error of the mean. ∗Difference between the low-dose and high-dose multimatrix mesalamine treatment groups. Both central and local reading values in panel (A) are for the DBM phase. ^†^Patients who did not have an endoscopy performed were excluded from the analysis of endoscopic remission. At Week 26, 33 patients receiving low-dose and 33 patients receiving high-dose multimatrix mesalamine had an endoscopy performed.

In the DBA phase, interpretation of the proportion of patients with a clinical and endoscopic response at Week 8 was limited by the small number of patients with endoscopic data available at both Week 0 and Week 8 (two of 27 patients for low-dose and three of 26 patients for high-dose multimatrix mesalamine). In the DBM phase, based on central reading at Week 26, 13 (31.0%) vs 11 (24.4%) patients receiving low-dose vs high-dose mesalamine had a clinical and endoscopic response. The difference between treatment groups was −6.5% (95% CI: −25.3 to 12.3; p = 0.54; Fig. 4A). Based on local reading at Week 26, 18 (42.9%) vs 12 (26.7%) patients receiving low-dose vs high-dose multimatrix mesalamine had a clinical and endoscopic response. The difference between treatment groups was −16.2% (95% CI: −36.0 to 3.6; p = 0.13; Fig. 4A).

In the DBA phase, the least-squares (LS) mean (standard error of the mean [SEM]) change from baseline in DUCS score at Week 8 was −17.9 (2.83) vs −23.3 (2.56) for patients in the low-dose vs high-dose groups. The difference in LS mean between treatment groups was −5.4 (95% CI: −13.1 to 2.4; p = 0.17; Fig. 4B). In the DBM phase, the LS mean (SEM) change from baseline in DUCS score at Week 26 was 1.9 (1.90) vs 4.8 (1.67) for patients receiving low-dose vs high-dose multimatrix mesalamine. The difference in LS mean was 3.0 (95% CI: −1.4 to 7.4; p = 0.18; Fig. 4B).

In the DBA phase, 10 (37.0%) vs 16 (61.5%) patients in the low-dose vs high-dose groups had an improvement in the PUCAI score of ≥20 points at Week 8. The difference between treatment groups was 24.5% (95% CI: −1.6 to 50.6; p = 0.07; Fig. 4C). In the DBM phase, 29 (69.0%) vs 27 (60.0%) patients in the low-dose vs high-dose groups were in remission (PUCAI score <10 points) at Week 26. The difference between treatment groups was −9.0% (95% CI: −29.1 to 11.0; p = 0.19; Fig. 4C).

Overall, 246 TEAEs were reported in 73 patients (69.5%); 23 TEAEs in 14 patients (13.3%) were considered related to the study drug (Table 3). Most patients experienced TEAEs that were mild (n = 38; 36.2%) or moderate (n = 28; 26.7%); severe TEAEs occurred in seven patients (6.7%). Eighteen serious TEAEs were reported in 12 patients (11.4%), and 29 TEAEs leading to discontinuation of the study drug were reported in 28 patients (26.7%; Table 3). Overall, more serious TEAEs (9/18) were reported as gastrointestinal disorders compared with those of other systems; the most common serious TEAEs were progression and/or exacerbation or flare of UC (3.8%) and anaemia (1.9%). No serious TEAEs were considered related to the study drug, and no deaths occurred during the study.

**Table 3:** TEAEs by dose and study phase: overall safety analysis set (N = 105).^a^

	DBA phase		DBM phase		OLA phase	Total
	Low-dose (n = 27)	High-dose (n = 26)	Low-dose (n = 42)	High-dose (n = 45)	High-dose (n = 18)	Overall (N = 105)
Any TEAE, n (%)	17 (63.0)	15 (57.7)	27 (64.3)	27 (60.0)	13 (72.2)	73 (69.5)
Serious TEAE, n (%)	4 (14.8)	0	3 (7.1)	2 (4.4)	3 (16.7)	12 (11.4)
TEAEs related to the study drug, n (%)	1 (3.7)	0	7 (16.7)	6 (13.3)	0	14 (13.3)
TEAEs leading to study drug discontinuation, n (%)	9 (33.3)	0	8 (19.0)	9 (20.0)	2 (11.1)	28 (26.7)
TEAEs leading to death, n (%)	0	0	0	0	0	0

DBA = double-blind acute; DBM = double-blind maintenance; OLA = open-label acute; TEAE = treatment-emergent adverse event.

a Patients who received one or more doses of multimatrix mesalamine.

In the DBA phase, the proportions of patients with TEAEs were similar for the low-dose (63.0%) and high-dose (57.7%) groups (Table 3). Among patients receiving low-dose multimatrix mesalamine, serious TEAEs were reported in 14.8% of patients. TEAEs leading to study drug discontinuation were reported in 33.3% of patients, and one patient (3.7%) had a single TEAE of dizziness that was considered related to the study drug. Among patients receiving high-dose multimatrix mesalamine, no serious TEAEs, TEAEs leading to discontinuation, or TEAEs related to the study drug were reported (Table 3). In the OLA phase, TEAEs were reported in 72.2% of patients who received high-dose multimatrix mesalamine (Table 3). Overall, 16.7% of patients experienced serious TEAEs, and 11.1% of patients experienced TEAEs leading to study drug discontinuation; no patient had TEAEs that were considered related to the study drug (Table 3). In the DBM phase, similar proportions of patients in the low-dose and high-dose groups reported any TEAEs (64.3% and 60.0%, respectively), serious TEAEs (7.1% and 4.4%), TEAEs leading to discontinuation of the study drug (19.0% and 20.0%), and TEAEs considered related to the study drug (16.7% and 13.3%; Table 3).

Frequently occurring (≥5% for the DBA or DBM phases and ≥10% for the OLA phase) TEAEs are shown in Table 4. The most common TEAEs by preferred term overall were UC (22.9%), abdominal pain (9.5%), and nasopharyngitis (8.6%).

**Table 4:** Frequently occurring TEAEs^a^: overall safety analysis set (N = 105).

TEAEs by MedDRA preferred term, n (%)	DBA phase		DBM phase		OLA phase^a^	Total
	Low-dose (n = 27)	High-dose (n = 26)	Low-dose (n = 42)	High-dose (n = 45)	High-dose (n = 18)	Overall (N = 105)
UC	8 (29.6)	0	6 (14.3)	8 (17.8)	2 (11.1)	24 (22.9)
Abdominal pain	1 (3.7)	2 (7.7)	3 (7.1)	5 (11.1)	1 (5.6)	10 (9.5)
Nasopharyngitis	0	0	3 (7.1)	6 (13.3)	0	9 (8.6)
Vomiting	2 (7.4)	1 (3.8)	0	3 (6.7)	1 (5.6)	7 (6.7)
Headache	2 (7.4)	2 (7.7)	2 (4.8)	2 (4.4)	0	6 (5.7)
Viral infection	1 (3.7)	2 (7.7)	2 (4.8)	1 (2.2)	0	6 (5.7)
Anaemia	1 (3.7)	1 (3.8)	1 (2.4)	1 (2.2)	2 (11.1)	6 (5.7)
Upper abdominal pain	0	1 (3.8)	3 (7.1)	2 (4.4)	0	6 (5.7)
Dyspepsia	1 (3.7)	3 (11.5)	0	2 (4.4)	0	5 (4.8)
Oropharyngeal pain	2 (7.4)	0	1 (2.4)	3 (6.7)	1 (5.6)	5 (4.8)
Upper respiratory tract infection	1 (3.7)	0	1 (2.4)	1 (2.2)	2 (11.1)	5 (4.8)
Pharyngitis	1 (3.7)	2 (7.7)	0	1 (2.2)	0	4 (3.8)
Cough	2 (7.4)	0	1 (2.4)	1 (2.2)	0	4 (3.8)
Pyrexia	2 (7.4)	1 (3.8)	0	1 (2.2)	0	3 (2.9)
Arthralgia	0	1 (3.8)	0	0	2 (11.1)	3 (2.9)
Rhinorrhoea	0	0	0	3 (6.7)	0	3 (2.9)

DBA = double-blind acute; DBM = double-blind maintenance; MedDRA = Medical Dictionary for Regulatory Activities; OLA = open-label acute; TEAE = treatment-emergent adverse event; UC = ulcerative colitis.

a Frequently occurring (≥5%) TEAEs, except for the OLA phase, which showed TEAEs in ≥10% of patients.

## Discussion

This study demonstrated that once-daily dosing of multimatrix mesalamine was effective in inducing and maintaining clinical response in paediatric patients with mild-to-moderate UC. A higher clinical response rate was observed in the high-dose (65.4%) compared with the low-dose (37.0%) group after 8 weeks of the DBA phase, whereas maintenance of clinical response was similar between the high-dose (53.3%) and low-dose (54.8%) groups after 26 weeks of double-blind treatment. Additionally, central and local readings confirmed similar rates of clinical and endoscopic response in low-dose and high-dose groups at Week 26. Discordance in results based on central vs local reading for the low-dose group may have been due to inter-reader variability in local reads.

DUCS and PUCAI were utilised in this study to assess disease activity and symptom burden in paediatric patients with mild-to-moderate UC. Although the DUCS instrument is similar to the PUCAI instrument, PUCAI is reported by clinicians whereas DUCS is based on an electronic diary for daily signs and symptoms that is completed by paediatric patients or, for younger patients, their caregivers.^19^^,^^20^ The LS mean change from baseline in DUCS showed similar responses for the low-dose vs high-dose groups for both the DBA and DBM phases. Similar to the clinical response rates, a numerically higher proportion of patients in the high-dose group (61.5%) vs the low-dose group (37.0%) showed an improvement in the PUCAI score at Week 8 in the DBA phase; in the DBM phase, similar proportions of patients in each treatment group (high-dose, 60.0%; low-dose, 69.0%) had PUCAI scores indicating remission at Week 26. As the psychometric properties of DUCS have not been established, DUCS results are for exploratory and illustrative purposes only.^19^ Furthermore, despite the subjective nature of PGA, its inclusion in the composite scoring system was likely driven by the strong correlation between PUCAI scores and PGA in evaluating disease activity, as well as the PUCAI score’s high predictability in determining the need for escalated medical therapy.^21^ It is also noteworthy that at the time of the study, there was limited evidence and expertise regarding alternative scoring systems.

Comparisons between clinical studies should be approached with caution owing to differences in patient populations, study designs, and methods; however, similar efficacy results were also observed in previous studies. In a randomised phase 4 study of once-daily vs twice-daily mesalamine induction in paediatric patients with mild-to-moderate UC, a response rate of 60% and a remission rate of 30% were observed at Week 6 with once-daily dosing.^14^ In a pooled analysis of two phase 3 studies of multimatrix mesalamine induction in adult patients with mild-to-moderate UC, the remission rate at Week 8 was 37.2% in patients receiving a dose of 2.4 g/d and 35.1% in those receiving a dose of 4.8 g/d.^18^ Additionally, in a phase 4 study of clinical recurrence in adult patients with quiescent UC treated with multimatrix mesalamine, 76.5% and 64.4% of patients remained recurrence free at Months 6 and 12, respectively, of the maintenance phase.^16^

The tolerability of multimatrix mesalamine in paediatric patients with UC was first demonstrated in a phase 1 study wherein 52 patients received once-daily multimatrix mesalamine for 7 days.^22^ In that study, all TEAEs were mild-to-moderate; no serious or fatal TEAEs or TEAEs leading to treatment discontinuation were observed. In the current study, TEAEs were mostly gastrointestinal disorders, which is expected in this study population, and most TEAEs were of mild or moderate severity. Most TEAEs that occurred in this study were consistent with the known safety profile for multimatrix mesalamine.^23^

Compliance with 5-ASA therapy is an important factor in the successful control of UC.^24^ Compliance rates in the DBA and DBM phases were high and comparable with those observed in previous studies of induction and maintenance with multimatrix mesalamine,^17^^,^^25^ as well as with previous studies that used once-daily and twice-daily oral mesalamine dosing in paediatric patients.^14^^,^^26^ The compliance rate in the OLA phase of this study was lower than that of the DBA and DBM phases, which may be due to the higher proportion of younger patients aged 5–13 years in the OLA phase (50.0%) vs the DBA (37.7%) and DBM phases (29.9%).

The main limitation of this study was that it was not powered to detect differences between treatment groups, as it was an estimation study with no formal hypothesis testing. Instead, the objective of this study, conducted in collaboration with the US FDA, was to estimate clinical response of two doses (low and high) of multimatrix mesalamine across a range of weight groups in children and adolescents. The sample size was selected based on practical considerations and agreement with the US FDA, where more than 100 patients were to be screened and up to 80 patients were to be enrolled in the DBA phase. However, the sample size for the DBA phase was reduced to 53 patients owing to difficulties with recruitment. Because of these challenges, the study was terminated to meet regulatory commitments to health agencies and to provide, in a timely manner, important safety and efficacy data for this unmet need in paediatric patients with UC. Furthermore, the considerable delay in publishing the study outcomes may be attributed to several factors including challenges in patient recruitment, takeover of companies involved in the study, the demanding schedules of the investigators, and disruptions caused by the COVID-19 pandemic.

In conclusion, high-dose multimatrix mesalamine achieved a higher clinical response rate than low-dose multimatrix mesalamine in the DBA phase of this study. In the DBM phase, similar proportions of patients in both treatment groups maintained clinical response; however, the proportion of patients who maintained clinical response was slightly higher in the low-dose group. The benefit-risk profile of multimatrix mesalamine in paediatric patients was favourable and comparable with that seen in the adult population in clinical studies.

## Contributors

NMC contributed to the design of the study and the collection and interpretation of the data. BK contributed to the collection, analysis, and interpretation of the data. JK contributed to the collection, analysis, and interpretation of the data. BC contributed to the collection, analysis, and interpretation of the data. MKT contributed to the conception and design of the study and the analysis of the data. All authors drafted and revised the article critically for important intellectual content and approved the final version of the article, including the authorship list. All authors had full access to all the data in the study and accept responsibility for the decision to submit for publication. Authors MKT and NMC have verified the underlying data.

## Data sharing statement

The datasets, including the redacted study protocol, redacted statistical analysis plan, and individual participant data supporting the results reported in this article, will be available to researchers who provide a methodologically sound proposal. The data will be provided after de-identification, in compliance with applicable privacy laws, data protection, and requirements for consent and anonymisation. To request access to data, the corresponding author may be contacted.

## Declaration of interests

NMC (into employer’s investigator accounts) received speaker fees, advisory board fees, and research funding from Eli Lilly, Takeda, AbbVie, Shire, Pfizer, Roche, Celgene, and 4D Pharma. BK received a grant from Shire for the study research conducted. JK (into employer’s investigator accounts) received speaker fees, advisory board fees, and research funding from Eli Lilly, Celltrion, and Nestlé. BC was an employee of Takeda at the time the study was conducted. MKT is an employee of Takeda and received no specific funding for this work.

## Appendix A Supplementary data

Supplementary Material

Protocol
