# Supplementary material for: Safety and efficacy of multimatrix mesalamine in paediatric patients with mild-to-moderate ulcerative colitis: a phase 3, randomised, double-blind study
Source: eClinicalMedicine. 2023 Oct 6;65:102232. doi: 10.1016/j.eclinm.2023.102232 (PMC10579284; doi:10.1016/j.eclinm.2023.102232)
Supplement: Supplementary Material [file mmc1.pdf]

SUPPLEMENTARY MATERIAL

Safety and efficacy of multimatrix mesalamine in paediatric patients with mild-to-moderate ulcerative colitis: a phase 3, randomised, double-blind study

Nicholas Michael Croft MBBS,[1,2] Bartosz Korczowski MD,[3] Jarosław Kierkuś MD,[4] Beatriz Caballero MD,[5] Manoj Kumar Thakur MS[6]

[1]Blizard Institute, Faculty of Medicine, Queen Mary University of London, London, UK; [2] Paediatric Gastroenterology, Royal London Children's Hospital, Barts Health NHS Trust, London, UK; [3]Department of Pediatrics and Pediatric Gastroenterology, College of Medical Sciences, University of Rzeszów, Rzeszów, Poland; [4]Department of Gastroenterology, Hepatology and Feeding Disorders, Children's Memorial Health Institute, Warsaw, Poland; [5]Takeda Clinical Science, Zurich, Switzerland; [6]Takeda Development Center Americas, Inc., Lexington, MA, USA.

Table of Contents

Supplementary Methods 3
Supplementary Table 1. Common prior excluded treatments 3
Supplementary Table 2. Outcome measures 5
Supplementary Results 8
Supplementary Table 3. Patient disposition 8

Supplementary Methods

Inclusion and exclusion criteria

Eligible patients were 5–17 years of age, with body weight of 18–90 kg and a confirmed diagnosis of mild-to-moderate ulcerative colitis (UC), established by sigmoidoscopy/colonoscopy with compatible histology. Patients were excluded if they had severe UC, defined as Physician's Global Assessment (PGA) score of 3 at baseline, Crohn's disease, bleeding disorders, and/or active peptic ulcer disease. The common prior treatments that are excluded medications for this study are detailed in Supplementary Table 1. During the study, administration of the following medication was not permitted and would result in withdrawal of the subject:

- Systemic or rectal corticosteroids
- Other medications containing 5-aminosalicylic acid (5-ASA; e.g. sulfasalazine or mesalamine/mesalazine), including topical administration
- Immunomodulators (e.g. 6-mercaptopurine, azathioprine, cyclosporine, and methotrexate)
- Biologics (e.g. anti–tumour necrosis factor agents, such as infliximab)
- Others: nicotine patches, any products containing fish oils (fish oils are prohibited unless on a stable dose 4 weeks prior to the screening visit [Visit 1] and throughout study participation), or any investigational or marketed drug that may interfere with the evaluation of the investigational product.

Non-steroidal anti-inflammatory drugs, anti-diarrhoeals, laxatives, antibiotics, and drugs that cause constipation are permitted for up to 10 consecutive days if taken for a condition unrelated to UC. Prophylactic use of a stable dose of aspirin up to 325 mg/day for cardiac disease was permitted. For mild/acute pain, acetaminophen was recommended. Administration of seasonal vaccines was permitted during the study; however, routine vaccinations should be deferred until after study completion where possible.

Supplementary Table 1. Common prior excluded treatments

Treatments	Minimum time before screening					
	3 days	7 days	21 days	4 weeks	6 weeks	No use permitted
Anti-diarrhoeals and/or anti-spasmodics*	X					
Antibiotics*		X				
Anti-inflammatory drugs*,†		X				
Vaccination/immunisation			X			
Systemic or rectal corticosteroids				X		
Immunomodulators					X	
Oral anticoagulants						X
Biologics						1 year

*Non-steroidal anti-inflammatory drugs, anti-diarrhoeals, laxatives, and antibiotics are permitted for up to 10 consecutive days if taken for a condition unrelated to UC

†Does not include current 5-aminosalicylic acid treatment

Study visits

Study visits occurred at the following time points after Visit 2, depending on the patient's partial Ulcerative Colitis Disease Activity Index (UC-DAI) score: Week 2, Week 4, and Week 8 (double-blind acute [DBA] phase withdrawal) in the DBA phase; Week 2, Week 4, and Week 8 (open-label acute [OLA] phase withdrawal) in the OLA phase; and Weeks 2–4, Week 13, and Week 26 (double-blind maintenance [DBM] phase withdrawal) in the DBM phase.

Statistical analysis

This study was an estimation study with no formal hypothesis testing; therefore, the study was not powered to detect differences between treatment groups. More than 100 patients were to be screened and up to 80 patients were to be enrolled in the DBA phase of the study. After agreement with the United States Food and Drug Administration (FDA), the sample size for the DBA phase was reduced to 53 patients owing to difficulties in recruitment. P-values are presented as descriptive statistics only. The overall safety analysis set consisted of

randomised patients who had taken ≥1 doses of multimatrix mesalamine. An independent data monitoring committee reviewed the safety data generated during the study.

In the DBA phase, the primary efficacy outcome was compared between treatment groups using an uncorrected chi-square test on the DBA safety analysis set. The null hypothesis was that no difference was seen in the proportion of patients with a clinical response at Week 8 when a high or low-dose multimatrix mesalamine was administered. The numbers and proportions of patients with a clinical response at Week 8 and the difference between treatment groups, together with the two-sided 95% confidence interval (CI) and the p-values, were calculated. The odds ratio (OR) comparing the two treatments and 95% CI were also calculated. In the DBM phase, the primary efficacy outcome was compared between treatment groups using a Cochran-Mantel-Haenszel test stratified for three levels of Week 8 responder status (entered DBM phase directly, responder at Week 8 of the DBA phase, or responder at Week 8 of the OLA phase) on the DBM phase safety analysis set. The null hypothesis was that no difference was seen in the proportion of patients with a clinical response at Week 26 when a high or low dose of multimatrix mesalamine was administered. The numbers and proportions of patients with a clinical response at Week 26 and the difference between treatment groups, together with the two-sided 95% CI and associated p-values were calculated. The OR comparing the two treatments, together with the 95% CI and p-value, was also calculated.

In the DBA phase, clinical and endoscopic responses at Week 8 were compared between treatment groups using a continuity-corrected chi-square test. The change in Daily Ulcerative Colitis Scale (DUCS) score from baseline to Week 8 was compared between treatment groups using an analysis of covariance, including treatment group as a factor and baseline DUCS score as a covariate. The percentage of patients with an improvement in Pediatric Ulcerative Colitis Activity Index (PUCAI) score from baseline to Week 8 was compared between treatment groups using a continuity-corrected chi-square test. In the DBM phase, clinical and endoscopic responses were compared between treatment groups using a Cochran-Mantel-Haenszel test adjusted for prior response status (entered the DBM phase directly, responder at Week 8 of the DBA phase, or responder at Week 8 of the OLA phase). The change in DUCS score from DBM phase Week 0 to Week 26 was compared between treatment groups using an analysis of covariance, including treatment group as a factor and DUCS score at DBM phase Week 0 and prior response status as covariates. The percentage of patients in remission at DBM phase Week 26 was compared between treatment groups using a Cochran-Mantel-Haenszel test adjusted for prior response status. Analyses were performed using SAS version 9·2 or higher (SAS Institute Inc., Cary, NC, USA).

Study outcome measures

The different outcome measures used in this study are detailed in Supplementary Table 2.

Supplementary Table 2. Outcome measures

Outcome measures	Definitions	Scoring system	Assessment during the study
**Physician's Global Assessment (PGA)**	The PGA is a physician-reported measure based on the daily record of abdominal discomfort and functional assessment and other observations such as physical findings, and the patient's performance status. The investigator should consider the following points:<br>• Rectal bleeding<br>• Stool frequency and consistency<br>• Night-time bowel movements<br>• Abdominal pain<br>• Impact to daily activities<br>• Physical findings<br>• Endoscopic findings (if endoscopy is performed)	PGA is scored on a scale from 0–3, where:<br>• 0: no active disease<br>• 1: mild disease<br>• 2: moderate disease<br>• 3: severe disease	DBA Phase: Weeks 0, 2, 4, and 8 or withdrawal<br>OLA Phase: Week 8<br>DBM Phase: Weeks 0 and 26 or withdrawal
**Partial Ulcerative Colitis Disease Activity Index (UC-DAI)**	The partial UC-DAI score includes:<br>• Stool frequency<br>• Rectal bleeding<br>• PGA	Each category will be assessed on a scale of 0–3, with maximum total partial UC-DAI score being 9·0<br>• Stool frequency and rectal bleeding was reported by the subjects/caregivers once a day before bedtime starting from the evening of screening visit to baseline visit<br>• For determination of the partial UC-DAI, the average of the symptom scores of the last available 3 days within the 5-day period immediately prior to the study visit was used<br><br>***Stool frequency:*** How many times did you poop than you normally do since you went to bed last night<br>• 0: I didn't poop more than I normally do<br>• 1: 1–2 times more than I normally do<br>• 2: 3–4 times more than I normally do<br>• 3: 5 or more times more than I normally do<br><br>***Rectal bleeding:*** Did you have blood in your poop?<br>• 0: I didn't see any blood in my poop<br>• 1: I saw streaks of blood (small amount) in my poop<br>• 2: I saw some blood (more than small amount) in my poop<br>• 3: I saw mostly blood (a lot) in my poop<br><br>Please refer to the PGA section to know about the scoring system	DBA Phase: Weeks 0 and 8<br>OLA Phase: Week 8<br>DBM Phase: Weeks 0 and 26<br><br>***Stool frequency and rectal bleeding***<br><br>DBA Phase: Weeks 0, 2, 4, and 8<br>OLA Phase: Weeks 2, 4, and 8<br>DBM Phase: Weeks 0, 13, and 26

Outcome measures	Definitions	Scoring system	Assessment during the study
**Modified Full UC-DAI Score**	The full UC-DAI is widely used to assess treatment efficacy in subjects with mild to moderate UC.* It consists of 4 individual parameters:<br>• Stool frequency<br>• Rectal bleeding<br>• Endoscopy score (mucosal appearance)<br>• PGA<br><br>In this study, a modified full UC-DAI scale is used, where an endoscopy score of mild disease does not include friability; instead, friability will be scored as 2: moderate disease	The total score was calculated by summing the individual scores for the 4 parameters (endoscopy score was based on the central reader's score). The maximum total modified full UC-DAI score being 12·0<br><br>Please refer to Partial UC-DAI score and PGA score section to know about the scoring system	DBA Phase: Weeks 0 and 8<br>OLA Phase: Week 8<br>DBM Phase: Weeks 0 and 26 (for patients who have had an endoscopy) or withdrawal
**Daily Ulcerative Colitis Scale (DUCS)**	DUCS is an electronic daily sign-and-symptom diary	Two versions have been developed: 1 patient-reported outcome (PRO) to be self-completed by children aged 8 to 17 years and 1 observer-reported outcome (ObsRO) to be completed by caregivers of children aged 5 to 10 years. Both the PRO and ObsRO were to be completed by children aged 8 to 10 years; however, the ObsRO was used for the secondary endpoint analysis	DBA Phase: Screening, weeks 0, 2, 4, and 8<br>OLA Phase: Weeks 2, 4, and 8<br>DBM Phase: Screening, weeks 0, 13, and 26
**Pediatric Ulcerative Colitis Activity Index (PUCAI) score**	The PUCAI is a physician-administered measure that focuses on 6 key signs and symptoms of UC and activity limitations	The PUCAI yields a total score ranging from 0–85, with higher scores being worse<br><br>• <10: Remission<br>• 11–30: Mild<br>• 31–64: Moderate<br>• >65: Severe	DBA Phase: Weeks 0 and 8<br>OLA Phase: Week 8<br>DBM Phase: Weeks 0 and 26 or withdrawal
**Clinical response**	Clinical and endoscopic response was defined as UC-DAI ≤1	Clinical response: UC-DAI ≤1<br><br>• Rectal bleeding=0<br>• Stool frequency≤1<br>• PGA=0	DBA Phase: Week 8<br>DBM Phase: Week 26
**Endoscopic response**	A flexible sigmoidoscopy or colonoscopy was performed. Endoscopies were centrally read and scored for this study, and endoscopic video or photographs were to be provided to the central reader for each endoscopy performed. Video images were preferred; however, photographic images were accepted when video images were not of sufficient quality, as determined by the central reader during individual site image evaluation prior to enrolment at the site.<br>All endoscopies were performed by the same investigator/endoscopist, if possible, to ensure consistency. Further details of requirements for video and photographic	Endoscopy score ranges from 0–3:<br><br>• 0: Normal (intact vascular pattern, no friability or granulation)<br>• 1: Mild (erythema, decreased vascular pattern, minimal granularity)<br>• 2: Moderate (marked erythema, granularity, friability, absent vascular pattern, bleeding with minimal trauma, no ulcerations)<br>• 3: Severe (ulceration, spontaneous bleeding)	DBA Phase: Week 8<br>OLA Phase: Week 8<br>DBM Phase: Week 26

Outcome measures	Definitions	Scoring system	Assessment during the study
	images were provided to study sites under separate cover by the central reader.		
**Clinical and endoscopic response**	Clinical and endoscopic response was defined as UC-DAI ≤2 with rectal bleeding=0, stool frequency ≤1, and PGA=0 and with mucosal healing (endoscopy score ≤1) based on central and local reading	Clinical and endoscopic response: UC-DAI ≤2<br>• Rectal bleeding=0<br>• Stool frequency ≤1<br>• PGA=0<br>• Endoscopy score ≤1	DBA Phase: Week 8<br>DBM Phase: Week 26

DBA=double-blind acute; DBM=double-blind maintenance; DUCS=Daily Ulcerative Colitis Scale; eCRF=electronic case report form; ObsRO=observer-reported outcome; OLA=open-label acute; PGA=Physician's Global Assessment; PRO=patient-reported outcome; PUCAI=Pediatric Ulcerative Colitis Activity Index; UC=ulcerative colitis; UC-DAI=Ulcerative Colitis Disease Activity Index.

*Schroeder KW, et al. N Engl J Med. 1987;317(26):1625-9; Sutherland LR, et al. Gastroenterology. 1987;92(6): 1894-8.

Supplementary Results

Supplementary Table 3. Patient disposition

	DBA phase			DBM phase			OLA phase	Total (N=107)
	Low-dose MMX (n=27)	High-dose MMX (n=26)	Overall (n=53)	Low-dose MMX (n=42)	High-dose MMX (n=45)	Overall (n=87)	High-dose MMX (n=18)	
**Completed the study/phase***	19 (70·4%)	22 (84·6%)	41 (77·4%)	32 (76·2%)	34 (75·6%)	66 (75·9%)	12 (66·7%)	65 (60·7%)
**Did not complete the study/phase†**	8 (29·6%)	4 (15·4%)	12 (22·6%)	‥	‥	‥	‥	42 (39·3%)
**Not continued in study**	6 (22·2%)	0	6 (11·3%)	‥	‥	‥	‥	2 (1·9%)
**Not enrolled in DBM phase**	‥	‥	‥	‥	‥	‥	‥	4 (3·7%)
**Did not have a follow-up**	‥	‥	‥	‥	‥	‥	‥	1 (0·9%)
**Discontinued from last phase**	‥	‥	‥	‥	‥	‥	‥	35 (32·7%)
**Primary reason for discontinuation from last phase‡**	‥	‥	‥	‥	‥	‥	‥	‥
**Adverse event**	1 (3·7%)	0	1 (1·9%)	3 (7·1%)	2 (4·4%)	5 (5·7%)	1 (5·6%)	7 (6·5%)
**Lack of efficacy**	5 (18·5%)	0	5 (9·4%)	6 (14·3%)	7 (15·6%)	13 (14·9%)	5 (27·8%)	23 (21·5%)
**Other**	0	0	0	1 (2·4%)	1 (2·2%)	2 (2·3%)	0	4 (3·7%)
**Missing**	0	0	0	0	1 (2·2%)	1 (1·1%)	0	1 (0·9%)

Data are presented as n (%)
DBA=double-blind acute; DBM=double-blind maintenance; MMX=multi-matrix system; OLA=open-label acute.
*'Completed the study' includes subjects who entered the DBM phase from one of the acute phases, completed the Week 26 visit, and had follow-up assessment within 7 days of the last dose of the investigational product, and subjects who entered directly into the DBM phase, completed the Week 26 visit, and had follow-up assessment within 7 days of the last dose of the investigational product.
†'Not continued in study' includes subjects who completed the DBA phase but did not continue into the OLA phase or the DBM phase. 'Not enrolled in double-blind maintenance phase' includes subjects who completed the OLA phase who did not enrol in the DBM phase.
‡Primary reason for discontinuation was the reason for discontinuation from the last phase that a subject was enrolled in, if the subject did not complete the phase. Subject 201-0017 withdrew from the DBM phase on 10 Oct 2018 but has missing reason for withdrawal. The subject had adverse events of diarrhoea and haematochezia on 23 Sep 2018.
Medication adherence was defined by use of 80%–120% of the study medication.
