# Supplementary material for: Safety and efficacy of multimatrix mesalamine in paediatric patients with mild-to-moderate ulcerative colitis: a phase 3, randomised, double-blind study
Source: eClinicalMedicine. 2023 Oct 6;65:102232. doi: 10.1016/j.eclinm.2023.102232 (PMC10579284; doi:10.1016/j.eclinm.2023.102232)
Supplement: Protocol [file mmc2.pdf]

PROTOCOL: SPD476-319

**TITLE:**	A Phase 3, Multicenter, Randomized, Double-blind Study to Determine the Safety and Efficacy of MMX Mesalamine/Mesalazine in Pediatric Subjects with Mild to Moderate Ulcerative Colitis, in both Acute and Maintenance Phases
**DRUG:**	SPD476, MMX® Mesalamine/mesalazine
**IND:**	66, 193
**EUDRACT NO.:**	2013-001744-65
**SPONSOR:**	Shire Development LLC<br>300 Shire Way, Lexington, MA 02421 USA
**PRINCIPAL/ COORDINATING INVESTIGATOR:**	[redacted], MD<br>[redacted]<br><br>[redacted], MD<br>[redacted]
**PROTOCOL HISTORY:**	Amendment 5: 10 Apr 2017, Version 6.0<br>Amendment 4: 28 Nov 2016, Version 5.0<br>Amendment 3: 09 Feb 2015, Version 4.0<br>Amendment 2: 14 Jul 2014, Version 3.0<br>Amendment 1: 17 Dec 2013, Version 2.0<br>Original Protocol: 11 Sep 2013, Version 1.0

This document contains confidential and proprietary information of Shire and is disclosed pursuant to confidentiality and non-disclosure obligations. This information should be used solely for the purposes for which it was provided and should not be copied, shared with, or disclosed to any third party without the express written consent of Shire.

PROTOCOL SIGNATURE PAGE

Sponsor's (Shir[redacted]	
Signature: [redacted]	Date: 11 April 2017
[redacted] , MD, Shire [redacted]	

Investigator's Acknowledgement

I have read this protocol for Shire Study SPD476-319.

Title: A Phase 3, Multicenter, Randomized, Double-blind Study to Determine the Safety and Efficacy of MMX Mesalamine/Mesalazine in Pediatric Subjects with Mild to Moderate Ulcerative Colitis, in both Acute and Maintenance Phases

I have fully discussed the objective(s) of this study and the contents of this protocol with the sponsor's representative.

I understand that the information in this protocol is confidential and should not be disclosed, other than to those directly involved in the execution or the scientific/ethical review of the study, without written authorization from the sponsor. It is, however, permissible to provide the information contained herein to a subject in order to obtain their consent to participate.

I agree to conduct this study according to this protocol and to comply with its requirements, subject to ethical and safety considerations and guidelines, and to conduct the study in accordance with International Council for Harmonisation guidelines on Good Clinical Practice and with the applicable regulatory requirements.

I understand that failure to comply with the requirements of the protocol may lead to the termination of my participation as an investigator for this study.

I understand that the sponsor may decide to suspend or prematurely terminate the study at any time for whatever reason; such a decision will be communicated to me in writing. Conversely, should I decide to withdraw from execution of the study I will communicate my intention immediately in writing to the sponsor.

Investigator Name and Address:<br>(please hand print or type)	

Signature: ______________________ **Date:** ______________

SUMMARY OF CHANGES FROM PREVIOUS VERSION

Protocol Amendments		
**Summary of Change(s) Since Last Version of Approved Protocol**		
**Amendment Number**<br>**5**	**Amendment Date**<br>**10 Apr 2017**	**Global/Country/Site Specific**<br>**Global**
**Description of Change**		**Section(s) Affected by Change**
Minor copyediting performed for consistency in style and presentation of the document.		Global
Modified approximate number of subjects to be screened to indicate that "up to" 80 subjects will be enrolled in the Double-blind Acute Phase of the study.<br>**Rationale: Edited this text to reflect the fact that less than 80 subjects may be enrolled in the acute phase of the study given the current recruitment challenges.**		Synopsis, Section 3.1
Modified to indicate that "at least" 80 subjects will be enrolled in the Double-blind Maintenance Phase of the study.		Synopsis, Section 3.1
Removed text indicating that enrollment in the Double-blind Maintenance Phase will be considered complete once 80 subjects have been randomized into this phase.<br>**Rationale: Capping enrollment in the Double-blind Maintenance Phase would prevent additional subjects from the Acute Phase of the study from rolling over into the Double-blind Maintenance Phase.**		Synopsis, Section 3.1
Removed text indicating that concomitant rectal corticosteroid use will be allowed following randomization.<br>**Rationale: Removed concomitant use of rectal corticosteroids in order to avoid confounding the efficacy analysis with use of another active compound.**		Synopsis, Section 4.2, Section 5.3
Added text indicating that subjects can be re-randomized if they initially fail randomization due to technical errors (eg, incorrect use of IVRS) or due to late or missing central laboratory or endoscopy results.		Synopsis, Section 4.3
Removed planned duration of enrollment period as this is not accurate for individual subject participation.		Synopsis
Removed text describing sample size calculations as this study is not powered to detect differences between treatment groups.		Synopsis, Section 9.6
Added urine pregnancy tests for all site visits and an explanatory footnote indicating that serum pregnancy tests will be performed at the screening and withdrawal visits for the Double-blind Acute, Open-label Acute, and Double-blind Maintenance Study Phases; urine pregnancy tests will be conducted at all other site visits.		Schedule of Assessments (Table 1, Table 2, Table 3)
Clarified that all pregnancy tests and will only be performed on females of childbearing potential.		Schedule of Assessments (Table 1, Table 2, Table 3), Section 4.4.1, Section 7.2.2.7
Added AE assessment during scheduled telephone calls and on Visit 5 (Table 3 only).		Schedule of Assessments (Table 1, Table 2, Table 3)

Protocol Amendments		
**Summary of Change(s) Since Last Version of Approved Protocol**		
**Amendment Number**<br>**5**	**Amendment Date**<br>**10 Apr 2017**	**Global/Country/Site Specific**<br>**Global**
**Description of Change**		**Section(s) Affected by Change**
Clarified footnote describing follow-up assessment to indicate that this will occur within 7 days of last dose of investigational product.		Schedule of Assessments (Table 1, Table 2, Table 3)
Expanded the window for acceptance of historical endoscopy results from 7 days to 21 days prior to the Screening Visit.		Schedule of Assessments (Table 1), Section 7.1.1.1, Section 7.1.1.3, Section 7.2.1.2

See Appendix 1 for protocol history, including all amendments.

EMERGENCY CONTACT INFORMATION

In the event of a serious adverse event, the investigator must fax or e-mail the Shire Clinical Trial Serious Adverse Event (SAE) Form within 24 hours to the Shire Pharmacovigilance Department. Applicable fax numbers and e-mail address can be found on the form (sent under separate cover) and below.

Shire Pharmacovigilance SAE Reporting:

United States (US) and Canadian sites: [redacted], or e-mail [redacted]

European Union (EU) /Rest of World (ROW) sites: [redacted], or e-mail [redacted]

Premier Research must also be notified of SAEs:

US and Canadian sites: fax [redacted], or e-mail [redacted]

EU/ROW sites: fax [redacted] or e-mail [redacted]

For all urgent protocol- or safety-related issues during and outside of business hours, the investigator must contact the Premier Research Medical Monitors:

North America (US and Canada) sites:
[redacted], MD, PhD, [redacted]
Central phone number: [redacted]

European and ROW sites
[redacted], MD, PhD, [redacted]
Central phone number: [redacted]

Backup Medical Monitor:
[redacted], MD; [redacted]
Central phone number: [redacted]

For non-emergencies, the Premier Research Medical Monitors may be contacted by e-mail:
[redacted]

PRODUCT QUALITY COMPLAINTS

Investigators are required to report investigational product quality complaints to Shire within 24 hours. This includes any instances wherein the quality or performance of a Shire product (marketed or investigational) does not meet expectations (eg, inadequate or faulty closure, product contamination) or that the product did not meet the specifications defined in the application for the product (eg, wrong product such that the label and contents are different products). For instructions on reporting adverse events (AEs) related to product complaints, see Section 8.

Please use the information below as applicable to report the Product Quality Complaint:

Origin of Product Quality Complaint	E-mail Address
North and South America	[redacted]
European Union and Rest of World	[redacted]

Telephone numbers (provided for reference if needed):

Shire, Lexington, MA (USA)

[redacted]

TABLE OF CONTENTS

PROTOCOL SIGNATURE PAGE 2
SUMMARY OF CHANGES FROM PREVIOUS VERSION 3
EMERGENCY CONTACT INFORMATION 5
PRODUCT QUALITY COMPLAINTS 6
TABLE OF CONTENTS 7
LIST OF TABLES 12
LIST OF FIGURES 12
LIST OF APPENDICES 12
ABBREVIATIONS 13
DEFINITIONS 14
STUDY SYNOPSIS 15
STUDY SCHEDULES 22
1. BACKGROUND INFORMATION 31
1.1 Indication and Current Treatment Options 31
1.2 Product Background and Clinical Information 31
2. STUDY OBJECTIVES AND PURPOSE 32
2.1 Rationale for the Study 32
2.2 Study Objectives 33
2.2.1 Primary Objectives 33
2.2.2 Secondary Objectives 33
2.2.3 Exploratory Objectives 34
3. STUDY DESIGN 34
3.1 Study Design and Flow Chart 34
3.2 Duration and Study Completion Definition 37
3.3 Sites and Regions 37
4. STUDY POPULATION 37
4.1 Inclusion Criteria 37
4.2 Exclusion Criteria 38
4.3 Re-randomization 40
4.4 Reproductive Potential 40
4.4.1 Female Contraception 40
4.5 Discontinuation of Subjects 41
4.5.1 Reasons for Discontinuation 41
4.5.2 Subjects ‘Lost to Follow-up’ Prior to Last Scheduled Visit 42
5. PRIOR AND CONCOMITANT TREATMENT 42

5.1 Prior Treatment 42
5.2 Concomitant Treatment 42
5.3 Prohibited Treatment 43
6. INVESTIGATIONAL PRODUCT 44
6.1 Identity of Investigational Product 44
6.1.1 Blinding the Treatment Assignment 44
6.2 Administration of Investigational Product(s) 44
6.2.1 Interactive Response Technology for Investigational Product Management 44
6.2.2 Allocation of Subjects to Treatment 44
6.2.3 Dosing 45
6.2.4 Unblinding the Treatment Assignment 46
6.3 Labeling, Packaging, Storage, and Handling 46
6.3.1 Labeling 46
6.3.2 Packaging 47
6.3.3 Storage 47
6.4 Drug Accountability 48
6.5 Subject Compliance 49
7. STUDY PROCEDURES 49
7.1 Study Schedule 49
7.1.1 Screening Period 49
7.1.1.1 Screening Visit (Visit 1) 49
7.1.1.2 Recommended Telephone Call (Day –7 to –4) 52
7.1.1.3 Baseline Visit (Visit 2) 52
7.1.1.4 Subject Visit Schedule Summary Post-baseline 54
7.1.2 Double-blind Acute Phase 54
7.1.2.1 Recommended Telephone Call (Day –7 to –4) 54
7.1.2.2 Double-blind Acute Phase Weeks 2 and 4 (Visits 3 and 3.1, respectively) 54
7.1.2.3 Double-blind Acute Phase Week 8/Withdrawal (Visit 3.2) 55
7.1.3 Open-label Acute Phase 56
7.1.3.1 Recommended Telephone Call (Days –7 to –4) 56
7.1.3.2 Open-label Acute Phase Weeks 2 and 4 (Visits 4 and 4.1, respectively) 56
7.1.3.3 Open-label Acute Phase Week 8/Withdrawal (Visit 4.2) 56
7.1.4 Double-blind Maintenance Phase 57
7.1.4.1 Recommended Telephone Call (Days –7 to –4) 57
7.1.4.2 Double-blind Maintenance Phase Weeks 2-4 (Visit 5) 57
7.1.4.3 Double-blind Maintenance Phase Week 13 (Visit 5.1) 58

7.1.4.4 Double-blind Maintenance Phase Week 26/Withdrawal (Visit 5.2)......58
7.1.5 Follow-up Period (Visit 6)......59
7.1.6 Additional Care of Subjects After the Study......59
7.2 Study Evaluations and Procedures......59
7.2.1 Efficacy......59
7.2.1.1 Partial UC-DAI Score......60
7.2.1.2 Modified Full UC-DAI Score......62
7.2.2 Safety......63
7.2.2.1 Medical and Medication History......64
7.2.2.2 Physical Examination......64
7.2.2.3 Height and Weight......64
7.2.2.4 Adverse Event Collection......64
7.2.2.5 Vital Signs......64
7.2.2.6 Clinical Laboratory Evaluations......65
7.2.2.7 Pregnancy Test......66
7.2.2.8 Stool Assessment......66
7.2.3 Pharmacokinetic Procedures......67
7.2.3.1 Blood Sample Collection and Handling Procedures......67
7.2.3.2 Shipment of Plasma Pharmacokinetic Samples......68
7.2.3.3 Plasma Drug Assay Methodology......68
[redacted] [redacted]......68
7.2.4.1 Health-related Quality-of-Life Assessments......69
7.2.5 Volume of Blood to be Drawn from Each Subject......71
8. ADVERSE AND SERIOUS ADVERSE EVENTS ASSESSMENT......71
8.1 Definition of Adverse Events, Period of Observation, Recording of Adverse Events......71
8.1.1 Severity Categorization......72
8.1.2 Relationship Categorization......72
8.1.3 Outcome Categorization......73
8.1.4 Symptoms of the Disease Under Study......73
8.1.5 Clinical Laboratory and Other Safety Evaluations......73
8.1.6 Pregnancy......74
8.1.7 Abuse, Misuse, Overdose, and Medication Error......74
8.2 Serious Adverse Event Procedures......75
8.2.1 Reference Safety Information......75
8.2.2 Reporting Procedures......75
8.2.3 Serious Adverse Event Definition......76
8.2.4 Serious Adverse Event Collection Timeframe......76

8.2.5 Serious Adverse Event Onset and Resolution Dates 76
8.2.6 Fatal Outcome 77
8.2.7 Regulatory Agency, Institutional Review Board, Ethics Committee, and Site Reporting....77
9. DATA MANAGEMENT AND STATISTICAL METHODS 77
9.1 Data Collection....77
9.2 Clinical Data Management....77
9.3 Data Handling Considerations....78
9.4 Statistical Analysis Process 78
9.5 Planned Interim Analysis, Adaptive Design, and Data Monitoring Committee 78
9.6 Sample Size Calculation and Power Considerations....79
9.7 Study Population 79
9.8 Efficacy Analyses....79
9.8.1 Primary Efficacy Endpoint 79
9.8.1.1 Double-blind Acute Phase....79
9.8.1.2 Double-blind Maintenance Phase....80
9.8.2 Sensitivity Analyses of the Primary Endpoint 80
9.8.3 Secondary Efficacy Endpoints....80
9.8.3.1 Double-blind Acute Phase....81
9.8.3.2 Double-blind Maintenance Phase....81
9.8.4 Exploratory Efficacy Endpoints....81
9.8.5 Exploratory Pharmacokinetic Endpoint....82
9.9 Safety Analyses 82
9.10 Other Analyses 83
9.10.1 Health-related Quality-of-Life Analyses 83
10. SPONSOR'S AND INVESTIGATOR'S RESPONSIBILITIES 83
10.1 Sponsor's Responsibilities 83
10.1.1 Good Clinical Practice Compliance....83
10.1.2 Indemnity/Liability and Insurance....83
10.1.3 Public Posting of Study Information....84
10.1.4 Submission of Summary of Clinical Study Report to Competent Authorities of Member States Concerned and Ethics Committees....84
10.1.5 Study Suspension, Termination, and Completion....84
10.2 Investigator's Responsibilities 84
10.2.1 Good Clinical Practice Compliance....84
10.2.2 Protocol Adherence and Investigator Agreement....85
10.2.3 Documentation and Retention of Records 85
10.2.3.1 Electronic Case Report Forms....85

10.2.3.2 Recording, Access, and Retention of Source Data and Study Documents 85
10.2.3.3 Audit/Inspection 86
10.2.3.4 Financial Disclosure 86
10.3 Ethical Considerations....86
10.3.1 Informed Consent/Assent....86
10.3.2 Institutional Review Board or Ethics Committee 87
10.4 Privacy and Confidentiality....88
10.5 Study Results/Publication Policy 88
11. REFERENCES 90
12. APPENDICES 91

LIST OF TABLES

Table 1: Double-blind Acute Phase 22
Table 2: Open-label Acute Phase 26
Table 3: Double-blind Maintenance Phase 28
Table 4: Minimally Required Number of Subjects per Weight Group 35
Table 5: Common Prior Excluded Treatments 43
Table 6: Volume of Blood to be Drawn From Each Subject 71

LIST OF FIGURES

Figure 1: Study Design Flow Chart 36

LIST OF APPENDICES

Appendix 1 Protocol History 92
Appendix 2 The Modified Full Ulcerative Colitis Disease Activity Index (UC-DAI) 100
Appendix 3 Subject Questionnaires 101
Appendix 4 Pediatric Ulcerative Colitis Activity Index 102
[redacted] [redacted] 103

ABBREVIATIONS

5-ASA	5-aminosalicylic acid
Ac-5-ASA	acetyl-5-aminosalicylic acid
AE	adverse event
CMH	Cochran-Mantel-Haenszel
CRO	contract research organization
DMC	Data Monitoring Committee
DUCS	Daily Ulcerative Colitis Scale
EC	Ethics Committee
eCRF	electronic case report form
e-diary	electronic diary
EU	European Union
FDA	Food and Drug Administration
GCP	Good Clinical Practice
β-HCG	beta-human chorionic gonadotropin
ICH	International Council for Harmonisation
IRB	Institutional Review Board
IRT	interactive response technology
LAR	legally authorized representative
MMX®	Multi-Matrix System
PGA	Physician's Global Assessment
PRO	patient-reported outcome
PUCAI	Pediatric Ulcerative Colitis Activity Index
ObsRO	observer-reported outcome
SAE	serious adverse event
SAP	statistical analysis plan
TEAE	treatment-emergent adverse event
UC	ulcerative colitis
UC-DAI	Ulcerative Colitis Disease Activity Index
US	United States

DEFINITIONS

Clinical response: partial Ulcerative Colitis Disease Activity Index (UC-DAI) ≤1 (with rectal bleeding=0 ***and*** stool frequency ≤1 ***and*** Physician's Global Assessment [PGA]=0)

Clinical and endoscopic response: UC-DAI ≤2 (with rectal bleeding=0 ***and*** stool frequency ≤1 ***and*** PGA=0) ***and*** with mucosal healing (endoscopy score ≤1) based on central reading

STUDY SYNOPSIS

**Protocol number:** SPD476-319	**Drug:** MMX Mesalamine/mesalazine
**Title of the study:** A Phase 3, Multicenter, Randomized, Double-blind Study to Determine the Safety and Efficacy of MMX Mesalamine/Mesalazine in Pediatric Subjects with Mild to Moderate Ulcerative Colitis, in both Acute and Maintenance Phases	
**Number of subjects (total and for each treatment arm):**<br>More than 100 subjects will be screened, and up to 80 subjects will be enrolled in the Double-blind Acute Phase of the study. More than 65 subjects will be screened (in addition to an expected 28 subjects who will enter the Double-blind Maintenance Phase from one of the Acute Phases) and at least 80 subjects will be enrolled in the Double-blind Maintenance Phase of the study.	
**Investigators:** Multicenter study	
**Sites and Regions:**<br>It is anticipated that sites in the following regions will participate: North America, Europe, and the Middle East. Approximately 48 active study sites are planned.	
**Study period (planned):**<br>2013-2018	**Clinical phase:** 3

Objectives:

Primary:

The primary objective of the Double-blind Acute Phase of the study is to assess clinical response to Multi-Matrix System (MMX®) mesalamine/mesalazine between a low and high dose in children and adolescents aged 5-17 years with mild to moderate ulcerative colitis (UC).

The primary objective of the Double-blind Maintenance Phase of the study is to assess maintenance of clinical response to MMX mesalamine/mesalazine between a low and high dose in children and adolescents aged 5-17 years who are in remission.

Secondary:

Double-blind Acute Phase

The secondary objectives of the Double-blind Acute Phase are to:

- Assess clinical and endoscopic response to treatment with MMX mesalamine/mesalazine between a low and high dose in children and adolescents aged 5-17 years with mild to moderate UC in the Double-blind Acute Phase
- Assess changes in the Daily Ulcerative Colitis Scale (DUCS) for children and caregivers between a low and high dose of MMX mesalamine/mesalazine in children and adolescents aged 5-17 years with mild to moderate UC in the Double-blind Acute Phase
- Assess improvement in Pediatric Ulcerative Colitis Activity Index (PUCAI) score between a low and high dose of MMX mesalamine/mesalazine in children and adolescents aged 5-17 years with mild to moderate UC in the Double-blind Acute Phase.

Double-blind Maintenance Phase

The secondary objectives of the Double-blind Maintenance Phase are to:

- Assess clinical and endoscopic response to treatment with MMX mesalamine/mesalazine between a low and high dose in children and adolescents aged 5-17 years who are in remission in the Double-blind Maintenance Phase

- Assess changes in the DUCS for children and caregivers between a low and high dose of MMX mesalamine/mesalazine in children and adolescents aged 5-17 years who are in remission in the Double-blind Maintenance Phase
- Assess remission using the PUCAI score between a low and high dose of MMX mesalamine/mesalazine in children and adolescents aged 5-17 years who are in remission in the Double-blind Maintenance Phase.

Safety

The safety objectives include the evaluation of the safety and tolerability of a low and high dose MMX mesalamine/mesalazine in children and adolescents aged 5-17 years with mild to moderate UC, in the Double-blind Acute Phase, the Open-label Acute Phase, and the Double-blind Maintenance Phase.

Rationale:

MMX mesalamine/mesalazine is approved for both the induction of remission in adult patients with mild to moderate UC and for maintenance of remission of UC. No data are available on the use of MMX mesalamine/mesalazine in children and adolescents; this study aims to evaluate the effect of MMX mesalamine/mesalazine in children and adolescents with mild to moderate UC or who are in remission aged 5-17 years.

Investigational product, dose, and mode of administration:

- MMX mesalamine/mesalazine, administered orally, randomized in a 1:1 ratio stratified by body weight group to the following doses:
  - 900mg/day or 1800mg/day for subjects weighing 18 to ≤23kg
  - 1200mg/day or 2400mg/day for subjects weighing >23 to ≤35kg
  - 1800mg/day or 3600mg/day for subjects weighing >35 to ≤50kg
  - 2400mg/day or 4800mg/day for subjects weighing >50 to ≤90kg
- MMX mesalamine/mesalazine tablet strengths 300, 600, and 1200mg with matching placebos to maintain the blind between low and high dose groups

Methodology:

This is a prospective study with a Screening Period of 3 to 21 days, an 8-week Double-blind Acute Phase, and a 26-week Double-blind Maintenance Phase. Each phase includes 2 arms, and subjects will be randomized to 1 of 2 doses (low or high) of MMX mesalamine/mesalazine (900-4800mg/day, given once daily) at the beginning of each phase. Randomization will be in a 1:1 ratio stratified by body weight group. There is an additional 8-week, Open-label Acute Phase for subjects who do not achieve a clinical response or who have withdrawn from the Double-blind Acute Phase after a minimum of 2 weeks and, in the investigator's opinion, have not benefited from treatment in the Double-blind Acute Phase. Clinical response is defined as partial Ulcerative Colitis Disease Activity Index (UC-DAI) ≤1 (with rectal bleeding=0 ***and*** stool frequency ≤1 ***and*** Physician's Global Assessment [PGA]=0) at the end of the Double-blind Acute Phase. In this Open-label Acute Phase, subjects are treated with the high dose of MMX mesalamine/mesalazine for their weight group.

At the Baseline Visit (Visit 2):

- Subjects with partial UC-DAI ≥2 (with a combined rectal bleeding ***and*** stool frequency score ≥1 ***and*** PGA=1 or 2) ***and*** with mucosal appearance (endoscopy score)=2 or 3 will be eligible to enter the Double-blind Acute Phase.
- Subjects with partial UC-DAI ≤1 (with rectal bleeding=0 ***and*** stool frequency ≤1 and PGA=0) ***and*** with mucosal appearance (endoscopy score)=0 or 1 will be eligible to enter the Double-blind Maintenance Phase.

Subjects with partial UC-DAI score between 1 and 2 will not be eligible to enter the Double-blind Acute Phase or the Double-blind Maintenance Phase.

A minimum of 1 score each for stool frequency and rectal bleeding must be available in order to randomize subjects into either the Double-blind Acute Phase or the Double-blind Maintenance Phase. Subjects with no symptom score data available will be considered screen failures as these symptom score data are used to calculate the partial UC-DAI score needed for enrollment.

Subjects who do not meet all the eligibility criteria for either the Double-blind Acute Phase or the Double-blind Maintenance Phase will be considered screen failures. Subjects may be rescreened after consultation with the medical monitor.

Subjects with a clinical response after completion of treatment in either the Double-blind Acute Phase or the Open-label Acute Phase will be eligible to enter the Double-blind Maintenance Phase based on partial UC-DAI score (ie, without additional endoscopy).

Subjects without a clinical response after completion of acute treatment in the Open-label Acute Phase must be withdrawn.

All subjects will have a Screening Visit (Visit 1), and screened subjects who are eligible will proceed to the Baseline Visit (Visit 2). Study visits occur at the following time points after the Baseline Visit (Visit 2), dependent on the subject's partial UC-DAI score:

Double-blind Acute Phase: Week 2, Week 4, Week 8 (Double-blind Acute Phase Withdrawal)

Open-label Acute Phase: Week 2, Week 4, Week 8 (Open-label Acute Phase Withdrawal)

Double-blind Maintenance Phase: Weeks 2-4 (investigational product dispensation only), Week 13, Week 26 (Double-blind Maintenance Phase Withdrawal)

Inclusion and exclusion criteria:

Inclusion Criteria:

General:

1. Ability to voluntarily provide written, signed, and dated (personally or via a legally authorized representative [LAR]) informed consent or assent as applicable to participate in the study.
2. Subject's parent/LAR demonstrates an understanding, ability, and willingness to fully comply with study procedures and restrictions.
3. Male and female children and adolescents aged 5-17 years, inclusive, at the Baseline Visit (Visit 2).
4. Body weight 18-90kg at the Screening Visit (Visit 1) and the Baseline Visit (Visit 2).
5. Male, or non-pregnant, non-lactating female who agrees to comply with any applicable contraceptive requirements of the protocol or females of non-childbearing potential.
6. Diagnosed with mild to moderate UC, established by sigmoidoscopy or colonoscopy with compatible histology. Screened subjects may also have an unconfirmed diagnosis of mild to moderate UC; however, the diagnosis of mild to moderate UC must have been established by sigmoidoscopy or colonoscopy with compatible histology prior to the Baseline Visit (Visit 2).
7. Subject is able to swallow the investigational product whole.

Double-blind Acute Phase:

8. Partial UC-DAI score ≥2 (with a combined rectal bleeding ***and*** stool frequency score ≥1 ***and*** PGA=1 or 2) ***and*** with mucosal appearance (endoscopy score)=2 or 3 at the Baseline Visit (Visit 2), for which 5-aminosalicylic acid (5-ASA) would be used as part of normal treatment.

9. If the subject is on 5-ASA treatment prior to study entry, then the dose must be stable. Stable therapy is defined as no change in dose, or no initiation of 5-ASA, from the onset of the current acute flare through discontinuation of therapy (required at the Baseline Visit [Visit 2]). Please see exclusion criterion 28 for an additional 5-ASA dose-related requirement.

Double-blind Maintenance Phase:

10. Partial UC-DAI score ≤1 (with rectal bleeding=0 ***and*** stool frequency ≤1 ***and*** PGA=0) ***and*** with mucosal appearance (endoscopy score)=0 or 1 at the Baseline Visit (Visit 2).

Exclusion Criteria:

General:

1. Severe UC (defined by PGA=3) at the Baseline Visit (Visit 2).
2. Crohn's disease, bleeding disorders, and/or active peptic ulcer disease.
3. Asthma, only if known to be 5-ASA sensitive.
4. Positive stool culture for enteric pathogens (including *Salmonella*, *Shigella*, *Yersinia*, *Aeromonas*, *Plesiomonas*, or *Campylobacter*). *Clostridium difficile* toxin, ova, or parasites present.
5. Previous colonic surgery.
6. History of hepatic impairment that, in the opinion of the investigator, may be incompatible with mesalamine treatment.
7. Moderate to severe renal impairment, in the opinion of the investigator.
8. Immediate or significant risk of toxic megacolon, in the opinion of the investigator.
9. History of pancreatitis.
10. History of Reye's syndrome.
11. Systemic or rectal corticosteroid use within 4 weeks prior to the Screening Visit (Visit 1). Topical, intranasal, or inhaled use is not exclusionary.
12. Immunomodulator (6-mercaptopurine, azathioprine) use within 6 weeks prior to the Screening Visit (Visit 1).
13. History of biologic (eg, anti-tumor necrosis factor agents, integrin receptor antagonists) within 1 year prior to the Screening Visit (Visit 1).
14. Antibiotic use within 7 days prior to the Screening Visit (Visit 1).
15. Any anti-inflammatory drugs, not including 5-ASA treatment but including non-steroidal anti-inflammatory drugs such as aspirin, cyclooxygenase-2 inhibitors or ibuprofen, within 7 days prior to the Screening Visit (Visit 1) unless used at over-the-counter levels for <3 days. However, prophylactic use of a stable dose of aspirin up to 325mg/day for cardiac disease is permitted.
16. Oral anticoagulant use (with the exception of subjects who have been on a stable dose of vitamin K antagonists such as warfarin for at least 90 days prior to the Screening Visit [Visit 1] and who are medically stable).
17. Treatment with anti-diarrheals and/or anti-spasmodics within 3 days prior to the Screening Visit (Visit 1).
18. Vaccination/immunization within 21 days prior to the Screening Visit (Visit 1).
19. Predisposed to the development of myo- or pericarditis.
20. Previously been randomized into this study and withdrawn.

21. Current or recurrent disease that could affect the action, absorption, or disposition of the investigational product, or could affect clinical or laboratory assessments.
22. Current or relevant history of physical or psychiatric illness, any medical disorder that may require treatment, including surgery, or make the subject unlikely to fully complete the study, or any condition that presents undue risk from the investigational product or procedures.
23. Current use of any medication (including over-the-counter, herbal, or homeopathic preparations) that could affect (improve or worsen) the condition being studied, or could affect the action, absorption, or disposition of the investigational product(s), or clinical or laboratory assessment. (Current use is defined as use within 21 days of the Screening Visit [Visit 1], or pharmacokinetic equivalent of 5 half-lives, whichever is longer.)
24. Known or suspected intolerance or hypersensitivity to the investigational product(s) (aminosalicylates [5-ASA]), closely related compounds (including but not limited to salicylates), or any of the stated ingredients.
25. Known history of alcohol or other substance abuse within the last year.
26. Within 30 days prior to the first dose of investigational product:

    - Have used an investigational product

    - Have been enrolled in a clinical study (including vaccine studies) that, in the investigator's opinion, may impact this study.

Double-blind Acute Phase:

27. Mucosal appearance (defined by endoscopic score=0 or 1) based on central reading or local reading (if central reading is not available) at the Screening Visit (Visit 1) or Baseline Visit (Visit 2).
28. Current relapse on a 5-ASA dose higher than the low dose tested in the study (900mg for subjects weighing 18 to ≤23kg, 1200mg for subjects weighing >23 to ≤35kg, 1800mg for subjects weighing >35 to ≤50kg, and 2400mg for subjects weighing >50 to ≤90kg).
29. Acute flare with onset >6 weeks prior to the Baseline Visit (Visit 2) if being treated with 5-ASA for the flare. There is no limit to the onset of flare prior to the Baseline Visit (Visit 2) if the flare is untreated.

Double-blind Maintenance Phase:

30. Mucosal appearance (endoscopic score)=2 or 3 based on central reading or local reading (if central reading is not available) at the Screening Visit (Visit 1) or Baseline Visit (Visit 2).

Re-randomization: Subjects with a clinical response, ie, partial UC-DAI ≤1 (defined as rectal bleeding=0 ***and*** stool frequency ≤1 ***and*** PGA=0) after completion of treatment in either the Double-blind or the Open-label Acute Phases will be eligible for re-randomization into the Double-blind Maintenance Phase provided they still meet all Baseline (Visit 2) inclusion and exclusion criteria (where re-assessed).

Note that an additional eligibility endoscopy is not required for entry into the Double-blind Maintenance Phase for subjects who achieve a clinical response after completion of treatment in either the Double-blind Acute Phase or the Open-label Acute Phase.

Subjects eligible for either the Double-blind Acute or the Double-blind Maintenance Phase can be re-randomized if they initially failed randomization for the following reasons: incorrect use of IVRS, late or missing central laboratory or endoscopy results, or other reasons of this type as long as they meet inclusion and exclusion criteria. Re-randomization can only take place after consultation with the medical monitor.

Subjects may be re-randomized into the Double-blind Maintenance Phase if they turned 18 during participation in either Acute Phase of the study.

Maximum duration of subject involvement in the study:

- Planned duration of screening period: 3 to 21 days
- Planned duration of treatment period: up to 42 weeks
- Planned duration of follow-up: 7 days

Endpoints and statistical analysis:

Primary Efficacy Endpoints

Double-blind Acute Phase:
The primary efficacy endpoint for the Double-blind Acute Phase is defined as the proportion of subjects with a clinical response (defined as partial UC-DAI ≤1 with rectal bleeding=0 ***and*** stool frequency ≤1 ***and*** PGA=0) at Week 8. This endpoint will be compared between treatment arms using a continuity-corrected chi-squared test.

Double-blind Maintenance Phase:
The primary efficacy endpoint for the Double-blind Maintenance Phase is defined as the proportion of subjects who have maintained a clinical response (defined as partial UC-DAI ≤1 with rectal bleeding=0 ***and*** stool frequency ≤1 ***and*** PGA=0) at Week 26. This endpoint will be compared between treatment arms using a Cochran-Mantel-Haenszel (CMH) test stratifying by Week 8 responder status.

Secondary Efficacy Endpoints

Double-blind Acute Phase:

- The proportion of subjects with a clinical and endoscopic response at Week 8, defined as UC-DAI ≤2 with rectal bleeding=0 ***and*** stool frequency ≤1 ***and*** PGA=0, ***and*** with mucosal healing (endoscopy score ≤1) based on central reading. In addition, there must be at least a 1-point reduction in endoscopy score from baseline. This endpoint will be compared between treatment arms using a continuity-corrected chi-squared test.
- The proportion of subjects with a clinical and endoscopic response at Week 8, defined as UC-DAI ≤2 with rectal bleeding=0 ***and*** stool frequency ≤1 ***and*** PGA=0, ***and*** with mucosal healing (endoscopy score ≤1) based on local reading. In addition, there must be at least a 1-point reduction in endoscopy score from baseline. This endpoint will be compared between treatment arms using a continuity-corrected chi-squared test.
- The change in the DUCS score from baseline to Week 8 of the Double-blind Acute Phase. This endpoint will be compared between treatment arms using an analysis of covariance, including the baseline DUCS score as a covariate in the model.
- The percentage of subjects with an improvement (change of ≥20 points) in PUCAI score from baseline to Week 8 of the Double-blind Acute Phase. This endpoint will be compared between treatment arms using a continuity-corrected chi-squared test.

Double-blind Maintenance Phase:

- The proportion of subjects who have maintained a clinical and endoscopic response at Week 26, defined as UC-DAI ≤2 with rectal bleeding=0 ***and*** stool frequency ≤1 ***and*** PGA=0, ***and*** with mucosal healing (endoscopy score ≤1) based on central reading. This endpoint will be compared between treatment arms using a CMH test stratifying by Week 8 responder status.
- The proportion of subjects who have maintained a clinical and endoscopic response at Week 26, defined as UC-DAI ≤2 with rectal bleeding=0 ***and*** stool frequency ≤1 ***and*** PGA=0, ***and*** with mucosal healing (endoscopy score ≤1) based on local reading. This endpoint will be compared between treatment arms using a CMH test stratifying by Week 8 responder status.
- The change in the DUCS score from Double-blind Maintenance Phase Week 0 to Week 26. This endpoint will be compared between treatment arms using an analysis of covariance, including the DUCS score at Double-blind Maintenance Phase Week 0 and Week 8 responder status as covariates in the model.

- The percentage of subjects in remission (PUCAI <10) at Double-blind Maintenance Phase Week 26. This endpoint will be compared between treatment arms using a CMH test stratifying by Week 8 responder status.

Sensitivity Analyses of the Primary Endpoint

The following sensitivity analyses will be performed for both the Double-blind Acute and the Double-blind Maintenance Phase primary endpoints:

- A modified clinical response, defined as partial UC-DAI≤1 with rectal bleeding=0 ***and*** stool frequency ≤1 will be analyzed in a similar way to the primary endpoint analysis in order to investigate the effect of removing the PGA component from the responder definition.
- An observed-case analysis will be performed, where data from those who completed Week 26 and those who withdrew from the study early and are presented and analyzed separately, using a similar analysis to the primary endpoint.
- A complete-case analysis, where subjects who withdraw early from the study will be excluded, will be performed in a similar way to the primary endpoint analysis.
- A last observation carried forward analysis, using data from the final on-treatment assessment, will be performed in a similar way to the primary endpoint analysis.
- Logistic modeling will be conducted to investigate the effect of doses in mg/kg on the clinical response in the Double-blind Acute and Double-blind Maintenance Phases.

Safety Endpoints

Adverse events (AEs) will be coded using the Medical Dictionary for Regulatory Activities. Treatment-emergent AEs (TEAEs) will be defined in the statistical analysis plan. The number of events, incidence, and percentage of TEAEs will be presented by treatment arm (ie, low or high dose), and overall, by system organ class, and by preferred term. Treatment-emergent AEs will be further summarized by treatment arm for severity and relationship to investigational product. Treatment-emergent AEs related to investigational product, TEAEs leading to withdrawal, serious AEs, and deaths will be summarized by treatment arm.

Clinical laboratory tests and vital signs, and their changes from baseline will be summarized by treatment arm and visit.

Sample Size Justification

The sample size of 80 subjects originally selected for each of the Double-blind Acute and Double-blind Maintenance Phases was chosen based on both practical considerations and on agreement with the United States Food and Drug Administration.

STUDY SCHEDULES

Table 1: Double-blind Acute Phase

Visit	1 (Screening)	Recommended Telephone call[a]	2 (Baseline)	3[b]	3.1	3.2[c, d, e, f]	6[g]
**Week/Month[h]**	**Day −21 to −3**	**Day −7 to −4**	**Week 0**	**Week 2**	**Week 4**	**Week 8/Withdrawal**	**Follow-up**
Informed consent	✔						
Inclusion/exclusion criteria	✔		✔				
Demographics	✔						
Medical and medication history	✔						
Physical examination	✔					✔	
Height	✔					✔	
Weight	✔		✔			✔	
Vital signs	✔		✔	✔	✔	✔	
Biochemistry (includes C-reactive protein) and hematology	✔					✔	
Pregnancy test (for females of childbearing potential only)[i]	✔		✔	✔	✔	✔	
Pharmacokinetic blood sampling (at participating sites only)						✔	
[redacted]			✔				
Urinalysis	✔					✔[j]	
[redacted]	✔						
Stool sample (culture)[k, l]	✔						

Table 1: Double-blind Acute Phase

Visit	1 (Screening)	Recommended Telephone call[a]	2 (Baseline)	3[b]	3.1	3.2[c, d, e, f]	6[g]
Week/Month[h]	Day –21 to –3	Day –7 to –4	Week 0	Week 2	Week 4	Week 8/Withdrawal	Follow-up
[redacted]	✓					✓	
Review DUCS instructions, ability to read and understand e-diary with subject/caregiver	✓						
Subject/caregiver e-diary entry: rectal bleeding, stool frequency, DUCS, and Overall Current Health[m]	✓		✓	✓	✓	✓	
Assessment of e-diary compliance[a]		✓					
Global Change in Health[l]				✓	✓	✓	
IMPACT III[n]			✓			✓	
PUCAI (where applicable)			✓			✓	
Average stool frequency[o, p, q]			✓	✓	✓	✓	
Average rectal bleeding[o, p, q]			✓	✓	✓	✓	
PGA[p, q]			✓	✓	✓	✓	
Endoscopy (flexible sigmoidoscopy or colonoscopy) for study eligibility[h, q, r]	✓		✓				
Endoscopy (flexible sigmoidoscopy or colonoscopy), where possible for subjects who are not entering into the Open-label Acute Phase)[q]						✓	
Calculate partial UC-DAI			✓			✓	
IRT entry	✓		✓	✓	✓	✓	

Table 1: Double-blind Acute Phase

Visit	1 (Screening)	Recommended Telephone call[a]	2 (Baseline)	3[b]	3.1	3.2[c, d, e, f]	6[g]
**Week/Month[h]**	**Day –21 to –3**	**Day –7 to –4**	**Week 0**	**Week 2**	**Week 4**	**Week 8/Withdrawal**	**Follow-up**
Investigational product dispensed			✔	✔	✔	✔[s]	
Investigational product collected and compliance calculated				✔	✔	✔	
Adverse events	✔	✔	✔	✔	✔	✔	✔
Concomitant medication	✔		✔	✔	✔	✔	✔
Re-randomization (subjects entering Double-blind Maintenance Phase only)						✔	

Abbreviations: DUCS=Daily Ulcerative Colitis Scale for children and caregivers; eCRF=electronic case report form; e-diary=electronic diary; IRT= interactive response technology; PGA=Physician's Global Assessment; PUCAI=Pediatric Ulcerative Colitis Activity Index; UC-DAI=Ulcerative Colitis Disease Activity Index.

[a] Approximately 4 to 7 days prior to the Baseline Visit (Visit 2) and prior to each subsequent visit (Visits 3, 3.1, and 3.2), it is recommended that site staff telephone the subject or the subject's caregiver to remind them to enter UC-DAI symptoms (rectal bleeding and stool frequency) into the e-diary every night, even if the subject has no symptoms.

[b] Subjects may enter into the Open-label Acute Phase beginning at Visit 3 (after a minimum of 2 weeks double-blind treatment) and at any time until the end of the Double-blind Acute Phase provided all criteria have been met.

[c] Subjects will continue into the Open-label Acute Phase if they have not met the criteria to enter the Double-blind Maintenance Phase and are eligible to continue into the Open-label Acute Phase.

[d] Subjects will continue into the Double-blind Maintenance Phase if partial UC-DAI ≤1 (with rectal bleeding=0 ***and*** stool frequency ≤1 ***and*** PGA=0) ***and*** with mucosal healing (endoscopy score)=0 or 1, and are eligible to continue into the Double-blind Maintenance Phase.

[e] This will be the final visit for subjects not entering the Open-label Acute Phase or the Double-blind Maintenance Phase.

[f] The Baseline Visit (Visit 2) does not need to be repeated for subjects entering the Double-blind Maintenance Phase at the end of the Double-blind Acute Phase. Visit 3.2 will be considered the Week 0 visit for the next phase into which subjects are continuing.

[g] Follow-up assessment in this phase applies only to subjects who are discontinuing the study; may be performed via telephone call within 7 days of the last dose of investigational product.

[h] A visit window of ±3 days is permitted for all study visits except for the Screening Visit (Visit 1) and the Baseline Visit (Visit 2), which must occur 3 to 21 days following the Screening Visit (Visit 1). If the initial endoscopy is not adequate for mucosal healing score assignment due to inadequate bowel preparation, to allow for a repeat endoscopy to be conducted, the period of the Screening Visit (Visit 1) can be extended by an additional 14 days from the date of the initial endoscopy.

[i] The serum pregnancy test will be performed for all female subjects of childbearing potential at the screening visit (Visit 1) and Week 8/Withdrawal (Visit 3.2). Urine pregnancy tests will be performed at all other visits.

[j] Urinalysis performed at this visit only for subjects who are not continuing into the Open-label Acute Phase.

[k] [redacted].

[l] Stool samples collected for culture may be processed by local laboratories; however, results from the central laboratory analysis will prevail and will be entered in the eCRF.

Table 1: Double-blind Acute Phase

Visit	1 (Screening)	Recommended Telephone call[a]	2 (Baseline)	3[b]	3.1	3.2[c, d, e, f]	6[g]
**Week/Month[h]**	**Day –21 to –3**	**Day –7 to –4**	**Week 0**	**Week 2**	**Week 4**	**Week 8/Withdrawal**	**Follow-up**

[m] Rectal bleeding, stool frequency, DUCS, and Overall Current Health e-diary entry is completed once a day before bedtime every day from the evening of the Screening Visit (Visit 1), preferably for at least the 3 days immediately prior to the Baseline Visit (Visit 2). Rectal bleeding, stool frequency, DUCS and Overall Current Health e-diary entry is completed once a day before bedtime for the 5 days immediately prior to Visits 3, 3.1, and 3.2 of the Double-blind Acute Phase.

[n] Global Change in Health to be completed by caregivers of children aged 5-10 years and by children aged 11-17 years; IMPACT III to be completed by children aged 8-17 years only (see Section 7.2.4.1).

[o] The average stool frequency and rectal bleeding scores will be calculated and available to the investigator through a report from the e-diary provider.

[p] Components of the partial UC-DAI.

[q] Components of the modified full UC-DAI.

[r] For subjects entering the Double-Blind Acute Phase only: Subjects with an unconfirmed diagnosis of UC must have endoscopy performed during the Screening Visit (Visit 1). Subjects with a previously confirmed diagnosis must have endoscopy at the Screening Visit (Visit 1) or at the Baseline Visit (Visit 2) if endoscopy has not been performed within 21 days prior to the Screening Visit (Visit 1).

[s] Only for subjects continuing into the Open- label Acute or Double- blind Maintenance Phase.

Table 2: Open-label Acute Phase

Visit	Recommended Telephone call[a]	4	4.1	4.2[b, c, d, e]	6[f]
Week[g]	Day –7 to –4	Week 2	Week 4	Week 8/Withdrawal	Follow-up
Physical examination				✓	
Height				✓	
Weight				✓	
Vital signs		✓	✓	✓	
Biochemistry (includes C-reactive protein) and hematology				✓	
Pregnancy test (for females of childbearing potential only)[h]		✓	✓	✓	
Pharmacokinetic blood sampling (at participating sites only)				✓	
Urinalysis				✓	
[redacted]				✓	
Subject e-diary entry: rectal bleeding, stool frequency, DUCS, and Overall Current Health[i]		✓	✓	✓	
Assessment of e-diary compliance[a]	✓				
PUCAI (where applicable)				✓	
Average stool frequency[j,k, l]		✓	✓	✓	
Average rectal bleeding[j, k, l]		✓	✓	✓	
PGA[k, l]				✓	
Endoscopy (flexible sigmoidoscopy or colonoscopy), where possible[k]				✓	
IRT entry		✓	✓	✓	
Calculate partial UC-DAI				✓	

Table 2: Open-label Acute Phase

Visit	Recommended Telephone call[a]	4	4.1	4.2[b, c, d, e]	6[f]
Week[g]	Day –7 to –4	Week 2	Week 4	Week 8/Withdrawal	Follow-up
Investigational product dispensed		✔	✔	✔ [m]	
Investigational product collected and compliance calculated		✔	✔	✔	
Adverse events	✔	✔	✔	✔	✔
Concomitant medication		✔	✔	✔	✔
Re-randomization (subjects entering Double-blind Maintenance Phase only)				✔	

Abbreviations: DUCS=Daily Ulcerative Colitis Scale for children and caregivers; e-diary=electronic diary; IRT= interactive response technology; PGA=Physician's Global Assessment; PUCAI=Pediatric Ulcerative Colitis Activity Index; UC-DAI=Ulcerative Colitis Disease Activity Index.

[a] Approximately 4 to 7 days prior to Visit 4 and prior to each subsequent visit (Visits 4.1 and 4.2), it is recommended that site staff telephone the subject or the subject's caregiver to remind them to enter UC-DAI symptoms (rectal bleeding and stool frequency) into the e-diary every night, even if the subject has no symptoms.

[b] Subjects will continue into the Double-blind Maintenance Phase if partial UC-DAI ≤1 (with rectal bleeding=0 ***and*** stool frequency ≤1 ***and*** PGA=0) and are eligible to continue into the Double-blind Maintenance Phase.

[c] Subjects will be withdrawn from the study if they have not achieved a partial UC-DAI ≤1 (with rectal bleeding=0 ***and*** stool frequency ≤1 ***and*** PGA=0).

[d] This will be the final visit for subjects not continuing into the Double-blind Maintenance Phase.

[e] The Baseline Visit (Visit 2) does not need to be repeated for subjects entering the Double-blind Maintenance Phase at the end of the Open-label Acute Phase. Visit 4.2 will be considered the Week 0 visit for the Double-blind Maintenance Phase.

[f] Follow-up assessment in this phase applies only to subjects who are discontinuing the study; may be performed via telephone call within 7 days of the last dose of investigational product.

[g] A visit window of ±3 days is permitted for all study visits.

[h] The serum pregnancy test will be performed for all female subjects of childbearing potential at Week 8/Withdrawal (Visit 4.2). Urine pregnancy tests will be performed at all other visits.

[i] Rectal bleeding, stool frequency, DUCS, and Overall Current Health e-diary entry is completed once a day before bedtime every day for 5 days immediately prior to Visits 4, 4.1 and 4.2 of the Open-label Acute Phase.

[j] The average stool frequency and rectal bleeding scores will be calculated and available to the investigator through a report from the e-diary provider.

[k] Components of the partial UC-DAI.

[l] Components of the modified full UC-DAI.

[m] Only for subjects continuing into the Double-blind Maintenance Phase.

Table 3: Double-blind Maintenance Phase

Visit	1 (Screening)[a]	Recommended Telephone call[b]	2 (Baseline)[a]	5	5.1	5.2	6[c]
Day/Month[d]	Day –21 to –3	Day –7 to –4	Week 0	Weeks 2-4	Week 13	Week 26/ Withdrawal	Follow-up
Informed consent	✔						
Inclusion/exclusion criteria	✔		✔				
Demographics	✔						
Medical and medication history	✔						
Physical examination	✔				✔	✔	
Height	✔					✔	
Weight	✔		✔			✔	
Vital signs	✔		✔		✔	✔	
Biochemistry (includes C-reactive protein) and hematology	✔					✔	
Pregnancy test (for females of childbearing potential only)[e]	✔		✔	✔	✔	✔	
Pharmacokinetic blood sampling (at participating sites only)						✔	
Urinalysis	✔					✔	
[redacted]			✔				
[redacted]	✔						
Stool sample (culture)[f g]	✔						
[redacted]	✔					✔	

Table 3: Double-blind Maintenance Phase

Visit	1 (Screening)[a]	Recommended Telephone call[b]	2 (Baseline)[a]	5	5.1	5.2	6[c]
Day/Month[d]	Day –21 to –3	Day –7 to –4	Week 0	Weeks 2-4	Week 13	Week 26/ Withdrawal	Follow-up
Review DUCS instructions, ability to read and understand e-diary with subject/caregiver	✔						
Subject/caregiver e-diary entry: rectal bleeding, stool frequency, DUCS, and Overall Current Health[h]	✔		✔		✔	✔	
Assessment of e-diary compliance[a]		✔					
Global Change in Health[i]						✔	
IMPACT III[i]			✔			✔	
PUCAI (where applicable)			✔			✔	
Average stool frequency [j, k, l]			✔		✔	✔	
Average rectal bleeding [j, k, l]			✔		✔	✔	
PGA [k, l]			✔			✔	
Endoscopy (flexible sigmoidoscopy or colonoscopy) for study eligibility[d, l, m]	✔		✔				
Endoscopy (flexible sigmoidoscopy or colonoscopy), where possible[l]						✔	
IRT entry	✔		✔	✔	✔	✔	
Calculate partial UC-DAI			✔			✔	
Investigational product dispensed			✔	✔	✔		
Investigational product collected and compliance calculated				✔	✔	✔	
Adverse events	✔	✔	✔	✔	✔	✔	✔
Concomitant medication	✔		✔		✔	✔	✔

Table 3: Double-blind Maintenance Phase

Visit	1 (Screening)[a]	Recommended Telephone call[b]	2 (Baseline)[a]	5	5.1	5.2	6[c]
Day/Month[d]	Day –21 to –3	Day –7 to –4	Week 0	Weeks 2-4	Week 13	Week 26/ Withdrawal	Follow-up

Abbreviations: DUCS=Daily Ulcerative Colitis Scale for children and caregivers; eCRF=electronic case report form; e-diary=electronic diary; IRT= interactive response technology; PGA=Physician's Global Assessment; PUCAI=Pediatric Ulcerative Colitis Activity Index; UC-DAI=Ulcerative Colitis Disease Activity Index.

[a] The Screening Visit (Visit 1) and the Baseline Visit (Visit 2) in Table 3 apply only to subjects entering directly into the Double-blind Maintenance Phase.

[b] Approximately 4 to 7 days prior to the Baseline Visit (Visit 2) and prior to each subsequent visit (Visits 5, 5.1, and 5.2), it is recommended that site staff telephone the subject or the subject's caregiver to remind them to enter UC-DAI symptoms (rectal bleeding and stool frequency) into the e-diary every night, even if the subject has no symptoms.

[c] Follow-up assessment may be performed via telephone call within 7 days of the last dose of investigational product.

[d] A visit window of ±3 days is permitted for all study visits except for the Screening Visit (Visit 1) (must be 3 to 21 days) and the Baseline Visit (Visit 2), which must occur 3 to 21 days following the Screening Visit (Visit 1). If the initial endoscopy is not adequate for mucosal healing score assignment due to inadequate bowel preparation, to allow for a repeat endoscopy to be conducted, the period of the Screening Visit (Visit 1) can be extended by an additional 14 days from the date of the initial endoscopy.

[e] The serum pregnancy test will be performed for all female subjects of childbearing potential at the screening visit (Visit 1) and Week 26/Withdrawal (Visit 5.2). Urine pregnancy tests will be performed at all other visits.

[f] [redacted]

Stool samples collected for culture may be processed by local laboratories in addition to the assessment by the central laboratory. Central laboratory results will prevail and, as such, they will be the data entered into the eCRF.

[h] Rectal bleeding, stool frequency, DUCS and Overall Current Health e-diary entry is completed once a day before bedtime every day during the Screening Visit (Visit 1), preferably for at least the 3 days immediately prior to the Baseline Visit (Visit 2). Rectal bleeding, stool frequency, DUCS and Overall Current Health e-diary entry is completed once a day before bedtime for the 5 days immediately prior to Visit 5.1 and Visit 5.2.

[i] Global Change in Health to be completed by caregivers of children aged 5-10 years and by children aged 11-17 years; IMPACT III to be completed by children aged 8-17 years only.

[j] The average stool frequency and rectal bleeding scores will be calculated and available to the investigator through a report from the e-diary provider.

[k] Components of the partial UC-DAI.

[l] Components of the modified full UC-DAI.

[m] For subjects entering the Double-blind Maintenance Phase directly: Subjects with a previously confirmed diagnosis must have endoscopy at the Screening Visit (Visit 1) or at the Baseline Visit (Visit 2). If the initial endoscopy is not adequate for mucosal healing score assignment due to inadequate bowel preparation, to allow for a repeat endoscopy to be conducted, the period of the Screening Visit (Visit 1) can be extended by an additional 14 days from the date of the initial endoscopy.

1. BACKGROUND INFORMATION

1.1 Indication and Current Treatment Options

Ulcerative colitis (UC) is a serious chronic inflammatory disease of the colon and rectum. The major clinical feature is bloody diarrhea. While an acute attack can occasionally be fatal, the characteristic course for most patients is one of remissions and exacerbations over a number of years. Life expectancy after recovery from a first attack is unchanged; however, morbidity can be long-lasting and may be associated with various extra-intestinal and late complications.

Extensive disease is observed in 60-80% of all cases when UC onset is in childhood, which is twice as often as in adults. Extensive disease results in a worse disease course, since disease course is correlated to disease severity. In children, 30-40% colectomy rates are seen at 10 years compared to 20% in adults with UC (Turner et al. 2012). Children may also develop unique and troublesome complications, including growth failure and delayed puberty. Although infrequent and geographically dispersed, UC has been reported as early as infancy. Most children with UC, however, are diagnosed in late childhood and adolescence. The peak incidence of inflammatory bowel disease occurs in patients between the ages of 15 and 25 years. Approximately 20% of patients with UC present before the age of 20 years (Mendeloff and Calkins 1988). There are approximately 50,000 children currently with UC in the US, primarily post-pubertal.

Clinical pediatricians agree that mesalamine has a role in therapy of UC in children, and pediatric gastroenterology guidelines contain reference to mesalamine therapy in UC. The side effect profile of mesalamine is broadly similar to placebo, and there are no indications from the literature that use of mesalamine in children is associated with a higher incidence of side effects.

The only acetyl-5-aminosalicylic acid (5-ASA)/mesalamine product that is currently licensed in the US for use in children/adolescents (aged 5 years and older with UC) and that is available in non-tablet formulation is balsalazide (COLAZAL®, Salix Pharmaceuticals, Inc) capsules. Balsalazide is a prodrug that is enzymatically cleaved in the colon to produce mesalamine. Capsule contents can be sprinkled on apple sauce if the capsules are too big to swallow.

The approval of 5-ASA products in other countries for use on children/adolescents varies.

1.2 Product Background and Clinical Information

Commercially available Multi-Matrix System (MMX®) mesalamine/mesalazine is a novel, high-strength formulation of 5-ASA (1.2g of mesalamine/mesalazine per tablet), which uses MMX technology designed to release 5-ASA throughout the colon. For the purpose of this study, 2 pediatric formulations (300mg and 600mg tablets) were developed, which are smaller than the commercially available product.

The patented MMX delivery system utilizes hydrophilic and lipophilic matrices enclosed within a gastro-resistant, pH-dependent coating to facilitate prolonged exposure of the colonic mucosa to 5-ASA. The gastro-resistant coating that covers the multi-matrix core delays release of 5-ASA until the tablet is exposed to approximately pH of 7.0 or higher, normally in the terminal ileum, at which time the coating disintegrates.

Due to the presence of the hydrophilic matrix, the tablet core is designed to swell upon exposure to intestinal fluid (in a manner similar to that of a sponge exposed to water) to form a viscous gel mass. This viscous gel mass is expected to slow the diffusion of the active drug from the tablet core into the colonic lumen. As the tablet and its surrounding gel mass progress through the colon, pieces of the gel mass are expected to gradually break away from the core. This allows release of some 5-ASA in proximity to the colonic mucosa. In addition, literature suggests that the hydrophilic matrix may also adhere to the colonic mucosa, thus delivering 5-ASA to its site of action.

The lipophilic matrix, together with 5-ASA, is interspersed within the hydrophilic matrix creating a partially hydrophobic environment, which slows the penetration of aqueous fluids into the tablet core. This is expected to slow the drug dissolution and provide a further extended release of the active medication, potentially prolonging therapeutic activity.

Always refer to the latest version of the MMX Mesalazine (Mesalamine) Investigator's Brochure for the overall risk/benefit assessment and the most accurate and current information regarding the drug metabolism, pharmacokinetics, efficacy and safety of MMX mesalamine/mesalazine.

2. STUDY OBJECTIVES AND PURPOSE

2.1 Rationale for the Study

MMX mesalamine/mesalazine is approved for both the induction of remission in adult patients with mild to moderate UC and for maintenance of remission of UC. Currently, limited data are available on the safety and efficacy of MMX mesalamine/mesalazine in children and adolescents with UC.

This study, a Pediatric Research Equity Act post-approval commitment with United States (US) Food and Drug Administration (FDA), is designed to find the appropriate dosage of MMX mesalamine/mesalazine for once-daily dosing across a range of weight groups, in children and adolescents aged 5-17 years with mild to moderate UC or who are in remission. As improved compliance may be achieved with once-daily dosing, a better overall treatment benefit may be obtained compared to existing alternatives for pediatric patients.

The data from this study will:

- Determine whether MMX mesalamine/mesalazine is safe and effective for use in children and adolescents (aged 5-17 years) with mild to moderate UC or who are in remission.
- Aid in selection of appropriate doses in the pediatric population.

The study allows all subjects to receive active MMX mesalamine/mesalazine. The doses for the study were determined from pharmacokinetic modeling using data from the SPD476-112 study, a pharmacokinetic study in children and adolescents with mild to moderate UC.

The inclusion of placebo and standard of care arms were considered in the design of this study. The use of placebo was considered impractical given the alternative treatments currently available to children and adolescents with UC.

As standard of care varies and there is no standard formulation or dose of 5-ASA licensed and approved across regions, a standard of care/comparator arm was not included.

2.2 Study Objectives

2.2.1 Primary Objectives

The primary objective of the Double-blind Acute Phase of the study is to assess clinical response to MMX mesalamine/mesalazine between a low and high dose in children and adolescents aged 5-17 years with mild to moderate UC.

The primary objective of the Double-blind Maintenance Phase of the study is to assess maintenance of clinical response to MMX mesalamine/mesalazine between a low and high dose in children and adolescents aged 5-17 years who are in remission.

2.2.2 Secondary Objectives

Double-blind Acute Phase

The secondary objectives of the Double-blind Acute Phase are to:

- Assess clinical and endoscopic response to treatment with MMX mesalamine/mesalazine between a low and high dose in children and adolescents aged 5-17 years with mild to moderate UC in the Double-blind Acute Phase
- Assess changes in the Daily Ulcerative Colitis Scale (DUCS) for children and caregivers between a low and high dose of MMX mesalamine/mesalazine in children and adolescents aged 5-17 years with mild to moderate UC in the Double-blind Acute Phase
- Assess improvement in the Pediatric Ulcerative Colitis Activity Index (PUCAI) score between a low and high dose of MMX mesalamine/mesalazine in children and adolescents aged 5-17 years with mild to moderate UC in the Double-blind Acute Phase.

Double-blind Maintenance Phase

The secondary objectives of the Double-blind Maintenance Phase are to:

- Assess clinical and endoscopic response to treatment with MMX mesalamine/mesalazine between a low and high dose in children and adolescents aged 5-17 years who are in remission in the Double-blind Maintenance Phase
- Assess changes in the DUCS for children and caregivers between a low and high dose of MMX mesalamine/mesalazine in children and adolescents aged 5-17 years who are in remission in the Double-blind Maintenance Phase
- Assess remission using the PUCAI score between a low and high dose of MMX mesalamine/mesalazine in children and adolescents aged 5-17 years who are in remission in the Double-blind Maintenance Phase.

Safety

The safety objectives include the evaluation of the safety and tolerability of a low and high dose of MMX mesalamine/mesalazine in children and adolescents aged 5-17 years with mild to moderate UC, in the Double-blind Acute Phase, the Open-label Acute Phase, and the Double-blind Maintenance Phase.

2.2.3 Exploratory Objectives

[redacted]

- [redacted]
- [redacted]

3. STUDY DESIGN

3.1 Study Design and Flow Chart

This study will enroll male and female children and adolescents aged 5-17 years. This is a prospective, parallel-group study with a Screening Period of 3 to 21 days, an 8-week Double-blind Acute Phase, and a 26-week Double-blind Maintenance Phase. More than 100 subjects will be screened, and up to 80 subjects will be enrolled in the Double-blind Acute Phase of the study. More than 65 subjects will be screened (in addition to an expected 28 subjects who will enter the Double-blind Maintenance Phase from one of the Acute Phases) and at least 80 subjects will be enrolled in the Double-blind Maintenance Phase of the study.

Subjects who do not meet the all the eligibility criteria for either the Double-blind Acute Phase or the Double-blind Maintenance Phase will be considered screen failures. Subjects may be rescreened after consultation with the medical monitor.

A minimum of 1 score each for stool frequency and rectal bleeding must be available in order to randomize subjects into either the Double-blind Acute Phase or the Double-blind Maintenance Phase (see Section 7.2.1.1 for details). Subjects with no symptom score data available will be considered screen failures as these symptom score data are used to calculate the partial UC-DAI score needed for enrollment.

Each of these phases includes 2 treatment arms, and subjects will be randomized at the beginning of each phase to 1 of 2 doses (low and high dose) of MMX mesalamine/mesalazine (900-4800mg/day, given once daily), stratified by weight group. There is an additional 8-week, Open-label Acute Phase for subjects who do not achieve a clinical response or who have withdrawn from the Double-blind Acute Phase and have met certain criteria (see Section 7.1.3). Clinical response is defined as partial UC-DAI ≤ 1 (with rectal bleeding=0 ***and*** stool frequency ≤ 1 ***and*** Physician's Global Assessment [PGA]=0) at the end of the Double-blind Acute Phase.

In this Open-label Acute Phase, subjects are treated with the high dose of MMX mesalamine/mesalazine for their weight group.

Subjects with a clinical response after completion of treatment in either the Double-blind Acute Phase or the Open-label Acute Phase will be eligible to enter the Double-blind Maintenance Phase based on partial UC-DAI score (ie, without additional endoscopy).

Subjects without a clinical response after completion of treatment in both the Double-blind Acute Phase and the Open-label Acute Phase will not be eligible to enter the Double-blind Maintenance Phase and must be withdrawn.

Subjects may enter the Double-blind Maintenance Phase directly following the Baseline Visit (Visit 2) if they have a partial UC-DAI ≤1 (with rectal bleeding=0 ***and*** stool frequency ≤1 ***and*** PGA=0) ***and*** with mucosal appearance (endoscopy score)=0 or 1 at that visit.

MMX mesalamine/mesalazine is dosed once daily by mouth. The selection of doses in this study is based on an approximate average of 43mg/kg for the low dose and 85mg/kg for the high dose in the 3 lower weight groups (18 to ≤23kg, >23 to ≤35kg and >35 to ≤50kg). These doses were selected based on standard of care dosing for other 5-ASA products in the pediatric population and pharmacokinetic dosing results. The dosing for the highest weight group (>50 to ≤90kg) is based on adult UC experience.

This study will be conducted in children and adolescents (aged 5-17 years) to collect data on the safety and efficacy of MMX mesalazine/mesalamine in this population. This study is a Pediatric Research Equity Act post-approval commitment with the US FDA.

Minimum enrollment criteria have been agreed with FDA as follows, based on subject weight group; these are summarized in Table 4.

Table 4: Minimally Required Number of Subjects per Weight Group

**Weight Group**	**Number of Subjects in Combined Low and High Dose** [a]
18 to ≤23kg	2
>23 to ≤35kg	12
>35 to ≤50kg	18
>50 to ≤90kg	36

[a] Randomization will be stratified by weight group with the aim of creating balance between the 2 dose groups within a weight group.

Figure 1: Study Design Flow Chart

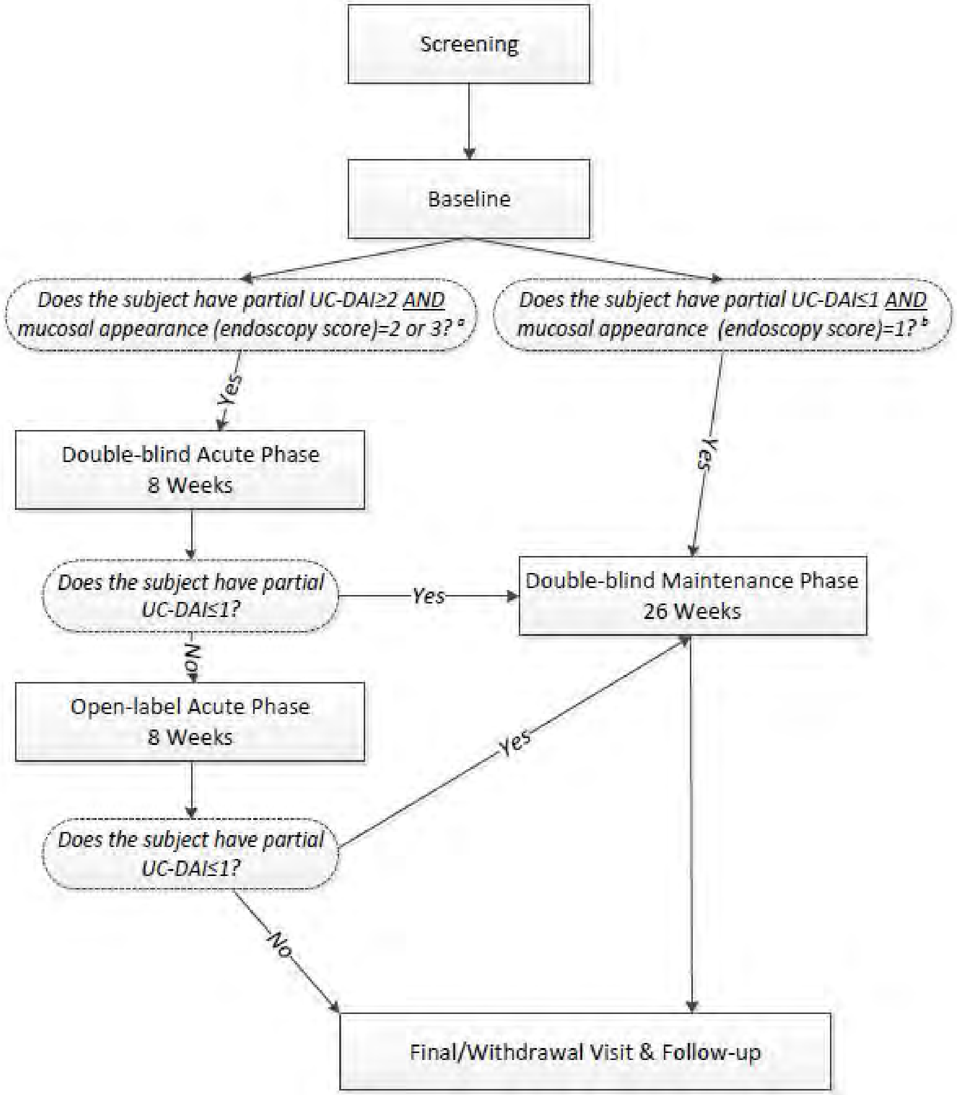

PGA=Physician's Global Assessment; UC-DAI=Ulcerative Colitis Disease Activity Index

[a] Subjects with partial UC-DAI ≥2 (with a combined rectal bleeding ***and*** stool frequency score ≥1 ***and*** PGA=1 or 2) ***and*** with mucosal appearance (endoscopy score)=2 or 3.

[b] Subjects with partial UC-DAI ≤1 (with rectal bleeding=0 ***and*** stool frequency ≤1 ***and*** PGA=0) ***and*** with mucosal appearance (endoscopy score)=0 or 1.

Notes: Solid boxes indicate study phases. Dashed ellipses indicate decision points.
Subjects will initially be randomized into either the 8-week Double-blind Acute Phase or the 26-week Double-blind Maintenance Phase based on partial UC-DAI and mucosal appearance scores as defined in Section 7.1.1.2. Subjects who do not achieve a clinical response or who have withdrawn from the Double-blind Acute Phase after a minimum of 2 weeks and, in the investigator's opinion, have not benefited from treatment in the Double-blind Acute Phase may enter the Open-label Phase, see Section 7.1.3. Subjects without a clinical response after completion of acute treatment in the Open-label Acute Phase must be withdrawn.

3.2 Duration and Study Completion Definition

The last subject contact for this study is the follow-up assessment (Visit 6).

The maximum duration of participation for a subject following randomization is expected to be approximately 42 weeks. The study will be completed in approximately 5 years.

The Study Completion Date is defined as the date the final subject, across all sites, completes their final protocol-defined assessment. Please note that this includes the follow-up visit or contact, whichever is later. The Study Completion Date is used to ascertain timing for study results posting and reporting.

3.3 Sites and Regions

It is anticipated that sites in approximately 8 countries will participate in this study. These countries are located in North America, Europe, and the Middle East. Approximately 48 sites are expected to enroll subjects into the study.

4. STUDY POPULATION

Each subject and/or caregiver must participate in the informed consent process and provide written informed consent/assent before any procedures specified in the protocol are performed.

4.1 Inclusion Criteria

The subject will not be considered eligible for the study without meeting all of the criteria below.

General:

1. Ability to voluntarily provide written, signed, and dated (personally or via a legally authorized representative [LAR]) informed consent or assent as applicable to participate in the study.
2. Subject's parent/LAR demonstrates an understanding, ability, and willingness to fully comply with study procedures and restrictions.
3. Male and female children and adolescents aged 5-17 years, inclusive, at the Baseline Visit (Visit 2).
4. Body weight 18-90 kg at the Screening Visit (Visit 1) and the Baseline Visit (Visit 2).
5. Male, or non-pregnant, non-lactating female who agrees to comply with any applicable contraceptive requirements of the protocol or females of non-childbearing potential.
6. Diagnosed with mild to moderate UC, established by sigmoidoscopy or colonoscopy with compatible histology. Screened subjects may also have an unconfirmed diagnosis of mild to moderate UC; however, the diagnosis of mild to moderate UC must have been established by sigmoidoscopy or colonoscopy with compatible histology prior to the Baseline Visit (Visit 2).

7. Subject is able to swallow the investigational product whole.

Double-blind Acute Phase:

8. Partial UC-DAI score ≥2 (with a combined rectal bleeding ***and*** stool frequency score ≥1 ***and*** PGA=1 or 2) ***and*** with mucosal appearance (endoscopy score)=2 or 3 at the Baseline Visit (Visit 2), for which 5-ASA would be used as part of normal treatment.
9. If the subject is on 5-ASA treatment prior to study entry, then the dose must be stable. Stable therapy is defined as no change in dose, or no initiation of 5-ASA, from the onset of the current acute flare through discontinuation of therapy (required at the Baseline Visit; Visit 2). See exclusion criterion 28 for an additional 5-ASA dose-related requirement.

Double-blind Maintenance Phase:

10. Partial UC-DAI ≤1 (with rectal bleeding=0 ***and*** stool frequency ≤1 ***and*** PGA=0) ***and*** with mucosal appearance (endoscopy score)=0 or 1 at the Baseline Visit (Visit 2).

4.2 Exclusion Criteria

Subjects are excluded from the study if any of the following exclusion criteria are met.

General:

1. Severe UC (defined by PGA=3) at the Baseline Visit (Visit 2).
2. Crohn's disease, bleeding disorders, and/or active peptic ulcer disease.
3. Asthma, only if known to be 5-ASA sensitive.
4. Positive stool culture for enteric pathogens (including *Salmonella*, *Shigella*, *Yersinia*, *Aeromonas*, *Plesiomonas*, or *Campylobacter*). *Clostridium difficile* toxin, ova, or parasites present.
5. Previous colonic surgery.
6. History of hepatic impairment that, in the opinion of the investigator, may be incompatible with mesalamine treatment.
7. Moderate to severe renal impairment, in the opinion of the investigator.
8. Immediate or significant risk of toxic megacolon, in the opinion of the investigator.
9. History of pancreatitis.
10. History of Reye's syndrome.
11. Systemic or rectal corticosteroid use within 4 weeks prior to the Screening Visit (Visit 1). Topical, intranasal, or inhaled use is not exclusionary.
12. Immunomodulator (eg, 6-mercaptopurine, azathioprine) use within 6 weeks prior to the Screening Visit (Visit 1).
13. History of biologic (eg, anti-tumor necrosis factor agents, integrin receptor antagonists) within 1 year prior to the Screening Visit (Visit 1).

14. Antibiotic use within 7 days prior to the Screening Visit (Visit 1).
15. Any anti-inflammatory drugs, not including 5-ASA treatment but including non-steroidal anti-inflammatory drugs such as aspirin, cyclooxygenase-2 inhibitors or ibuprofen, within 7 days prior to the Screening Visit (Visit 1) unless used at over-the-counter levels for <3 days. However, prophylactic use of a stable dose of aspirin up to 325mg/day for cardiac disease is permitted.
16. Oral anticoagulant use (with the exception of subjects who have been on a stable dose of vitamin K antagonists such as warfarin for at least 90 days prior to the Screening Visit [Visit 1] and who are medically stable).
17. Treatment with anti-diarrheals and/or anti-spasmodics within 3 days prior to the Screening Visit (Visit 1).
18. Vaccination/immunization within 21 days prior to the Screening Visit (Visit 1).
19. Predisposed to the development of myo- or pericarditis.
20. Previously been randomized into this study and withdrawn.
21. Current or recurrent disease that could affect the action, absorption, or disposition of the investigational product, or could affect clinical or laboratory assessments.
22. Current or relevant history of physical or psychiatric illness, any medical disorder that may require treatment, including surgery, or make the subject unlikely to fully complete the study, or any condition that presents undue risk from the investigational product or procedures.
23. Current use of any medication (including over-the-counter, herbal, or homeopathic preparations) that could affect (improve or worsen) the condition being studied, or could affect the action, absorption, or disposition of the investigational product(s), or clinical or laboratory assessment. (Current use is defined as use within 21 days of the Screening Visit [Visit 1], or pharmacokinetic equivalent of 5 half-lives, whichever is longer.) See Section 5 (Prior and Concomitant Treatment) for a list of prohibited and restricted medications.
24. Known or suspected intolerance or hypersensitivity to the investigational product(s) (aminosalicylates eg, 5-ASA), closely related compounds (including but not limited to salicylates), or any of the stated ingredients.
25. Known history of alcohol or other substance abuse within the last year.
26. Within 30 days prior to the first dose of investigational product:
    - Have used an investigational product
    - Have been enrolled in a clinical study (including vaccine studies) that, in the investigator’s opinion, may impact this study.

Double-blind Acute Phase:

27. Mucosal appearance (defined by endoscopic score=0 or 1) based on central reading or local reading (if central reading is not available) at the Screening Visit (Visit 1) or Baseline Visit (Visit 2).
28. Current relapse on a 5-ASA dose higher than the low dose tested in the study (900mg for subjects weighing 18 to ≤23kg, 1200mg for subjects weighing >23 to ≤35kg, 1800mg for subjects weighing >35 to ≤50kg, and 2400mg for subjects weighing >50 to ≤90kg).
29. Acute flare with onset >6 weeks prior to the Baseline Visit (Visit 2) if being treated with 5-ASA for the flare. There is no limit to the onset of flare prior to the Baseline Visit (Visit 2) if the flare is untreated.

Double-blind Maintenance Phase:

30. Mucosal appearance (endoscopic score)=2 or 3 based on central reading or local reading (if central reading is not available) at the Screening Visit (Visit 1) or Baseline Visit (Visit 2).

4.3 Re-randomization

Subjects with a clinical response, ie, partial UC-DAI ≤1 (with rectal bleeding=0 *and* stool frequency ≤1 *and* PGA=0) after completion of treatment in either the Double-blind or the Open-label Acute Phases will be eligible for re-randomization into the Double-blind Maintenance Phase provided they still meet all Baseline Visit (Visit 2) inclusion and exclusion criteria (where re-assessed).

Note that an additional eligibility endoscopy is not required for entry into the Double-blind Maintenance Phase for subjects who achieve a clinical response after completion of treatment in either the Double-blind Acute Phase or the Open-label Acute Phase.

Subjects eligible for either the Double-blind Acute or the Double-blind Maintenance Phase can be re-randomized if they initially failed randomization for the following reasons: incorrect use of IVRS, late or missing central laboratory or endoscopy results, or other reasons of this type as long as they meet inclusion and exclusion criteria. Re-randomization can only take place after consultation with the medical monitor.

Subjects may be re-randomized into the Double-blind Maintenance Phase if they turned 18 years of age during participation in either Acute Phase of the study.

4.4 Reproductive Potential

4.4.1 Female Contraception

Sexually active females of childbearing potential must be using an acceptable form of contraception. Females of childbearing potential must be advised to use acceptable contraceptives throughout the study period and for 30 days following the last dose of investigational product. If hormonal contraceptives are used they should be administered according to the package insert.

Females of childbearing potential who are not currently sexually active must agree to use acceptable contraception, as defined below, if they become sexually active during the period of the study and 30 days following the last dose of investigational product.

All female subjects of childbearing potential must have a negative serum beta-human chorionic gonadotropin (β-HCG) pregnancy test at the Screening Visit (Visit 1) and prior to randomization. Female subjects of childbearing potential must abstain from sexual activity that could result in pregnancy or agree to use acceptable methods of contraception. Abstinence can only be accepted if this is true abstinence in line with the preferred and usual lifestyle of the subject.

Acceptable methods of contraception are:

- Intrauterine devices plus condoms
- Double-barrier methods (eg, condoms and diaphragms with spermicidal gel or foam)
- Hormonal contraceptives (oral, depot, patch, injectable, or vaginal ring), stabilized for at least 30 days prior to the Screening Visit (Visit 1), plus condoms. Note: if subject becomes sexually active during the study, they must use 1 of the other acceptable methods noted above in addition to the hormonal contraceptive until it has been stabilized for 30 days.

4.5 Discontinuation of Subjects

A subject may withdraw from the study at any time for any reason without prejudice to their future medical care by the physician or at the institution. The investigator or sponsor may withdraw the subject at any time (eg, in the interest of subject safety). The investigator is encouraged to discuss withdrawal of a subject from investigational product with the medical monitor when possible.

If investigational product is discontinued, regardless of the reason, the evaluations listed for the Withdrawal Visit (Visits 3.2, 4.2, or 5.2, depending on the phase) are to be performed as completely as possible. Whenever possible, all discontinued subjects should also undergo the protocol-specified follow-up. Comments (spontaneous or elicited) or complaints made by the subject must be recorded in the source documents. The reason for termination, date of stopping investigational product, and the total amount of investigational product taken must be recorded in source documents.

Subjects who discontinue will not be replaced.

4.5.1 Reasons for Discontinuation

The reason for withdrawal must be determined by the investigator and recorded in the subject’s medical record and in the electronic case report form (eCRF). If a subject is withdrawn for more than 1 reason, each reason should be documented in the source document and the most clinically relevant reason should be entered in the eCRF.

Reasons for discontinuation include but are not limited to:

- Adverse event
- Protocol violation
- Withdrawal by subject
- Lost to follow-up
- Lack of efficacy
- Other (if "Other" is selected, the investigator must provide further details).

4.5.2 Subjects 'Lost to Follow-up' Prior to Last Scheduled Visit

A minimum of 3 documented attempts must be made to contact any subject lost to follow-up at any time point prior to the last scheduled contact (office visit or telephone contact). At least 1 of these documented attempts must include a written communication sent to the subject's last known address via courier or mail (with an acknowledgement of receipt request) asking that they return to the site for final safety evaluations and return any unused investigational product.

In addition to the 3 attempts made to prevent subjects from being lost to follow-up during the conduct of the study, Shire's plan is to regularly evaluate each site for missing data. Any trends will be identified and addressed with specific sites should they emerge. Issues leading to missing data will be identified and retraining of the site and support for the site will be implemented as appropriate.

5. PRIOR AND CONCOMITANT TREATMENT

All non-study treatment including but not limited to herbal treatments and vitamins received within 21 days prior to the Screening Visit (Visit 1) (or pharmacokinetic equivalent of 5 half-lives, whichever is longer) and through the final study contact (including protocol-defined follow-up period) must be recorded in the appropriate eCRF page.

5.1 Prior Treatment

Prior treatment given for the subject's UC should be recorded for the year prior to the Screening Visit (Visit 1), where available. All other prior treatment, including but not limited to herbal treatments and vitamins, received within 21 days of the date of first dose of investigational product must be recorded.

5.2 Concomitant Treatment

Concomitant treatment refers to all treatment taken between the dates of the first dose of investigational product and the end of the follow-up period, inclusive. Concomitant treatment information must be recorded in the appropriate eCRF page.

During the Screening Visit (Visit 1), the subject must remain on their current 5-ASA medication for UC (if applicable) at a stable dose until the Baseline Visit (Visit 2), at which time current treatment must be discontinued.

5.3 Prohibited Treatment

Table 5 details the common prior treatments that are excluded medications for this study.

Table 5: Common Prior Excluded Treatments

	Minimum Time Before Screening					
**Treatment**	**3 days**	**7 days**	**21 days**	**4 weeks**	**6 weeks**	**No Use Permitted**
Anti-diarrheals and/or anti-spasmodics[a]	X					
Antibiotics[a]		X				
Anti-inflammatory drugs[a, b, c]		X				
Vaccination/immunization			X			
Systemic or rectal corticosteroids				X		
Immunomodulators					X	
Oral anticoagulants[b]						X
Biologics						1 year

[a] Non-steroidal anti-inflammatory drugs, anti-diarrheals, laxatives, and antibiotics are permitted for up to 10 consecutive days if taken for a condition unrelated to UC
[b] See Section 4.2 (Exclusion Criteria) for exceptions to these excluded prior medications.
[c] Does not include current 5-aminosalicylic acid treatment

Administration of the following medication during the study is not permitted and will result in withdrawal of the subject:

- Systemic or rectal corticosteroids.
- Other medications containing 5-ASA (eg, sulfasalazine or mesalamine/mesalazine), including topical administration
- Immunomodulators (eg, 6-mercaptopurine, azathioprine, cyclosporine, methotrexate)
- Biologics (eg, anti-tumor necrosis factor agents, such as infliximab).

The following are also prohibited: nicotine patches, any products containing fish oils (fish oils are prohibited unless on a stable dose 4 weeks prior to the Screening Visit [Visit 1] and throughout study participation), or any investigational or marketed drug that may interfere with the evaluation of the investigational product.

In the event of any prohibited UC medication use during the Double-blind Maintenance Phase, the subject should notify the investigator who will assess if the subject is experiencing a possible acute flare.

Non-steroidal anti-inflammatory drugs, anti-diarrheals, laxatives, antibiotics, and drugs that cause constipation are permitted for up to 10 consecutive days if taken for a condition unrelated to UC. Prophylactic use of a stable dose of aspirin up to 325mg/day for cardiac disease is permitted. For mild, acute pain, acetaminophen is recommended.

Administration of seasonal vaccines is permitted during the study; however, routine vaccinations should be deferred until after study completion where possible.

6. INVESTIGATIONAL PRODUCT

6.1 Identity of Investigational Product

The test product is MMX mesalamine/mesalazine, which will be provided in 300, 600, and 1200mg tablet form. Matching placebo will also be provided in tablet form. Additional information is provided in the current MMX Mesalazine (Mesalamine) Investigator's Brochure.

6.1.1 Blinding the Treatment Assignment

This study includes double-blind treatment in the Double-blind Acute Phase and Double-blind Maintenance Phase. The actual double-blind treatment given to individual subjects is determined by a randomization schedule which will be automatically assigned by the interactive response technology (IRT). Placebo, which exactly matches the investigational product, will be used in the blister packs to provide the same number and size tablets for each of the doses within a weight group and maintain the blind between the low and high dose groups.

6.2 Administration of Investigational Product(s)

6.2.1 Interactive Response Technology for Investigational Product Management

Interactive response technology will be used for the following investigational product tasks:

- Randomization
- Supply management
- Inventory management and supply ordering
- Expiration tracking
- Returns
- Emergency unblinding.

6.2.2 Allocation of Subjects to Treatment

This study includes 2 double-blind phases with low and high-dose MMX mesalamine/mesalazine. The actual double-blind treatment given to individual subjects is determined by a randomization schedule stratified by weight group. Randomization will be a 1:1 ratio between low and high doses.

Subject numbers are assigned to all subjects as they consent to take part in the study. Within each site (numbered uniquely within a protocol), the subject number is assigned to subjects according to the sequence of presentation for study participation.

The randomization number represents a unique number corresponding to investigational product allocated to the subject, once eligibility has been determined.

Individual subject doses in the Double-blind Acute Phase are automatically assigned by the IRT based on subject weight group at the Baseline Visit (Visit 2). If the subject is directly entering the Double-blind Maintenance Phase, individual doses are also automatically assigned by the IRT based on subject weight group at the Baseline Visit (Visit 2).

Individual subject open-label treatment is automatically assigned by the IRT and will be the high dose based on the subject's weight group at Visit 3.2 (Week 8/Withdrawal of the Double-blind Acute Phase).

Individual subject double-blind doses for subjects being re-randomized to the Double-blind Maintenance Phase after completion of treatment at Visit 3.2 (Week 8 of the Double-blind Acute Phase) or Visit 4.2 (Week 8 of the Open-label Acute Phase) are automatically assigned by the IRT based on subject weight group at the time of re-randomization.

6.2.3 Dosing

Subjects entering the Double-blind Acute Phase or entering the Double-blind Maintenance Phase directly will be randomized to low- or high-dose MMX mesalamine/mesalazine stratified by their weight group at the Baseline Visit (Visit 2).

Subjects entering the Double-blind Maintenance Phase after completing 1 or both of the Acute Phases will be re-randomized to low- or high-dose MMX mesalamine/mesalazine at the start of the Double-blind Maintenance Phase at Visit 3.2 (Week 8 of the Double-blind Acute Phase) or 4.2 (Week 8 of the Open-label Acute Phase), as applicable, based on their weight at the time of re-randomization.

Doses will be as follows:

- 900 or 1800mg/day for subjects weighing 18 to ≤23kg
- 1200 or 2400mg/day for subjects weighing >23 to ≤35kg
- 1800 or 3600mg/day for subjects weighing >35 to ≤50kg
- 2400 or 4800mg/day for subjects weighing >50 to ≤90kg.

Subjects participating in the Open-label Acute Phase will receive the high dose for their weight group. The subject's weight at Visit 3.2 (Week 8/Withdrawal of the Double-blind Acute Phase) will be used to determine the high dose assigned to the subject.

Investigational product will be administered orally, once daily, in accordance with the MMX Mesalazine (Mesalamine) Investigator's Brochure. No titration or dose adjustment is allowed.

Dosing with investigational product will begin the day of the Baseline Visit (Visit 2) if the subject's currently prescribed 5-ASA treatment (if applicable) has not yet been taken that day. If the subject's currently prescribed 5-ASA treatment (if applicable) has been taken the day of the Baseline Visit (Visit 2), dosing with investigational product will begin the day after the Baseline Visit (Visit 2).

When proceeding through study phases, dosing with newly prescribed investigational product will begin the day of the study visit if the subject's currently prescribed investigational product from the current phase has not yet been taken that day. If the subject's currently prescribed investigational treatment has been taken the day of the study visit, dosing with newly prescribed investigational product will begin the day after the first study visit of the new phase.

6.2.4 Unblinding the Treatment Assignment

The treatment assignment must not be broken during the study except in emergency situations where the identification of the investigational product is required for further treatment of the subject. Prior to unblinding, and if the situation allows, the investigator should first contact the medical monitor.

In the event that the treatment assignment is broken, the date, the signature of the person who broke the code and the reason for breaking the code are recorded in the IRT and the subject's medical records. Any code breaks that occur must be reported to the medical monitor/sponsor as soon as possible.

Code break IRT access is held by the pharmacist/designated person at the site and by the medical monitor, or designee, for the study. There will be a provision for unblinding to ensure adequate treatment of the subject in the case of an emergency.

6.3 Labeling, Packaging, Storage, and Handling

6.3.1 Labeling

Labels containing study information and pack identification are applied to the investigational product(s) packaging.

All investigational product is labeled with a minimum of the protocol number, medication identification number (open-label supplies only), dosage form (including product name and quantity in pack), directions for use, storage conditions, expiry), batch number and/or packaging reference, the statements ‘For clinical trial use only’, and/or ‘CAUTION: New Drug - Limited by Federal (or US) Law to Investigational Use’, and ‘Keep out of reach of children’, and the sponsor's name and address. Any additional labeling requirements for participating countries will also be included on the label.

Space is allocated on the label so that the site representative can record a unique subject identifier and initials (where permitted by local laws).

Additional labels may, on a case-by-case basis, be applied to the investigational product in order to satisfy local or institutional requirements, but must not:

- Contradict the clinical study label
- Obscure the clinical study label

Additional labels may not be added without the sponsor's prior full agreement.

If an investigator site requires a subject's name to be included on the additional label in order for the subject to receive investigational product, the investigator site must ensure that the additional label is removed or de-identified before returning investigational product to the sponsor nominated contractor for destruction. By removing the label with the subject's name, the sponsor will not have access to the subject's identity and hence confidentiality will be preserved.

6.3.2 Packaging

Double-blind investigational product is packaged in blister pack wallets containing a 1-week supply of product (tablet quantity dependent on weight group). Placebo, which exactly matches the investigational product, will be used in the blister packs to provide the same number and size tablets for each of the doses within a weight group and maintain the blind between the low and high dose groups.

Open-label investigational product is packaged in 100cc high-density polyethylene bottles in the following counts:

- 600mg=60 tablets/bottle
- 1200mg=40 tablets/bottle

Changes to sponsor-supplied packaging prior to dosing may not occur without full agreement in advance by the sponsor.

6.3.3 Storage

The investigator has overall responsibility for ensuring that investigational product is stored in a secure, limited-access location. Limited responsibility may be delegated to the pharmacy or member of the study team, but this delegation must be documented. Investigational products are distributed by the pharmacy or nominated member of the study team. The pharmacist/nominated team member will enter the unique subject identifier and initials (where permitted by local law) on the investigational product blister pack wallets/bottles as they are distributed.

Investigational product must be stored in accordance with labeled storage conditions. Temperature monitoring is required at the storage location to ensure that the investigational product is maintained within an established temperature range. The investigator is responsible for ensuring that the temperature is monitored throughout the duration of the study and that records are maintained; the temperature should be monitored continuously by using either an in-house system, a mechanical recording device such as a calibrated chart recorder, or by manual means, such that both minimum and maximum thermometric values over a specific time period can be recorded and retrieved as required.

Such a device (ie, certified min/max thermometer) would require manual resetting upon each recording. The sponsor must be notified immediately upon discovery of any excursion from the established range. Temperature excursions will require site investigation as to cause and remediation. The sponsor will determine the ultimate impact of excursions on the investigational product and will provide supportive documentation as necessary. Under no circumstances should the product be dispensed to subjects until the impact has been determined and the product is deemed appropriate for use by the sponsor.

The sponsor should be notified immediately if there are any changes to the storage area of the investigational product that could affect the integrity of the product(s), eg, fumigation of a storage room.

6.4 Drug Accountability

Investigators will be provided with sufficient amounts of the investigational product to carry out this protocol for the agreed number of subjects. The investigator or designee will acknowledge receipt of the investigational product, documenting shipment content and condition. Accurate records of all investigational product dispensed, used, returned, and/or destroyed must be maintained as detailed further in this section.

The investigator has overall responsibility for administering/dispensing investigational product. Where permissible, tasks may be delegated to a qualified designee (eg, a pharmacist) who is adequately trained in the protocol and who works under the direct supervision of the investigator. This delegation must be documented in the applicable study delegation of authority form.

The investigator or his/her designee (as documented by the investigator in the applicable study delegation of authority form) will dispense the investigational product only to subjects included in this study following the procedures set out in the study protocol. Each subject will be given only the investigational product carrying his/her treatment assignment. All dispensed medication will be documented in the eCRFs and/or other investigational product record. The investigator is responsible for assuring the retrieval of all study supplies from subjects. No investigational product stock or returned inventory from a Shire-sponsored study may be removed from the site where originally shipped without prior knowledge and consent by the sponsor. If such transfer is authorized by the sponsor, all applicable local, state, and national laws must be adhered to for the transfer.

The sponsor or its representatives must be permitted access to review the supplies storage and distribution procedures and records provided that the blind of the study is not compromised.

At the end of the study, or as instructed by the sponsor, all unused stock, subject-returned investigational product, and empty/used investigational product packaging are to be sent to a nominated contractor on behalf of the sponsor. Investigational product being returned to the sponsor's designated contractors must be counted and verified by clinical site personnel and the sponsor (or designated contract research organization [CRO]). For unused supplies where the original supplied tamper-evident feature is verified as intact, the tamper-evident feature must not be broken and the labeled amount is to be documented in lieu of counting. Shipment return forms, when used, must be signed prior to shipment from the site.

Validated electronic return systems (ie, IRT) do not require a shipment form. Returned investigational product must be packed in a tamper-evident manner to ensure product integrity. Contact the sponsor for authorization to return any investigational product prior to shipment. Shipment of all returned investigational product must comply with local, state, and national laws.

Based on entries in the site drug accountability forms, it must be possible to reconcile investigational products delivered with those used and returned. All investigational products must be accounted for and all discrepancies investigated and documented to the sponsor's satisfaction.

6.5 Subject Compliance

Subjects must be instructed to bring their unused investigational product and empty/used investigational product packaging to every visit. Investigational product accountability must be assessed at the container/packaging level for unused investigational product that is contained within the original tamper-evident sealed container (eg, bottles) or at the individual count level for opened containers/packaging. The pharmacist/nominated person will record details on the investigational product accountability form.

If investigational product accountability determines that the subject has not taken investigational product at the dose prescribed, the subject and/or caregiver will be re-educated as to the protocol dosing requirements.

7. STUDY PROCEDURES

7.1 Study Schedule

See Table 1, Table 2, and Table 3 for study procedures.

7.1.1 Screening Period

7.1.1.1 Screening Visit (Visit 1)

The Screening Visit (Visit 1) for this study is a period of 3 to 21 days prior to the Baseline Visit (Visit 2). The Screening Visit (Visit 1) date will be considered the date of the first screening procedure (eg, informed consent/assent signature, during the Screening Period). Individual screening procedures may be performed at any time during the period of the Screening Visit (Visit 1). All procedures must be completed within this 3- to 21-day period before the Baseline Visit (Visit 2).

The Screening Visit (Visit 1) period of 3 to 21 days may be extended if the eligibility endoscopy is not adequate for assigning a mucosal appearance score due to inadequate bowel preparation as determined by the principal investigator or central endoscopy reader. In this case, if endoscopy is to be repeated during the period of the Screening Visit (Visit 1), the Baseline Visit (Visit 2) may occur up to 14 days following initial endoscopy performed during the Screening Visit (Visit 1). The repeat endoscopy must be planned in adequate time to allow the Baseline Visit (Visit 2) to occur at a maximum of 14 days following the initial endoscopy.

Each subject and/or caregiver will be instructed to continue completion of the electronic-diary (e-diary) every night before bedtime during the period of the extended Screening Visit (Visit 1).

During the period of the Screening Visit (Visit 1), the subject must remain on their current 5-ASA medication for UC (if applicable) at a stable dose until the Baseline Visit (Visit 2), at which time current treatment must be discontinued. If being screened for entry into the Double-blind Acute Phase, the current 5-ASA dose must not be higher than the MMX mesalamine/mesalazine low dose for the subject's weight group.

Subjects should not use any other concomitant medication for their UC during the period of the Screening Visit (Visit 1). Weight will be measured to ensure that this parameter is within protocol-defined limits during the period of the Screening Visit (Visit 1). Weight will be re-assessed at the Baseline Visit (Visit 2) for eligible subjects to determine randomization stratification.

Collection of stool samples for all assessments must occur before administration of any bowel preparations for endoscopy if samples are collected on the same day as bowel preparation administration. Stool samples collected for culture may be processed by local laboratories; however, results from the central laboratory analysis will prevail and will be entered into the eCRF.

Subjects without a confirmed diagnosis of UC:

Endoscopy (flexible sigmoidoscopy or colonoscopy), including biopsies taken for confirmatory histology, must be performed during the period of the Screening Visit (Visit 1). The biopsies taken for confirmation of UC diagnosis will be sent to the site's local laboratory. Confirmatory results to verify the subject's UC diagnosis must be received prior to the Baseline Visit (Visit 2) for randomization.

Subjects with a historical diagnosis of UC:

Subjects who have been diagnosed with UC (endoscopic and histologic reports of diagnosis are available) prior to the Screening Visit (Visit 1) will be considered to have a historical diagnosis of UC. For subjects who have had endoscopy within the 21 days prior to screening, if the endoscopy is consistent with UC, the subject may proceed to the Screening Visit (Visit 1) with a historical diagnosis of UC. The subject can be randomized once the endoscopy confirms the diagnosis of UC. The histologic confirmation of UC can be available after the Baseline Visit (Visit 2). (See next paragraph below for additional details.)

Subjects who enter the Double-blind Acute Phase:

If a standard of care endoscopy (flexible sigmoidoscopy or colonoscopy, including biopsy) has been performed within 21 days prior to the Screening Visit (Visit 1) and appropriate video or photographs of the procedure were obtained, these assessments may be used in lieu of performing an endoscopy during the period of the Screening Visit (Visit 1) or at the Baseline Visit (Visit 2). The video or photographs of this endoscopy must be de-identified and submitted to the study's central reader for confirmation of study eligibility after informed consent for the study is obtained.

The local laboratory histology report confirming the diagnosis of UC may be pending upon entry into the study at the Screening Visit (Visit 1); however, the confirmatory histology report must be available before randomization at the Baseline Visit (Visit 2). All other protocol-required assessments (including all laboratory assessments, ie, blood, urine and stool collection) must be performed for the Screening Visit (Visit 1) even if recent results are available.

For subject's whose historical diagnosis of UC was made more than 21 days prior to the Screening Visit (Visit 1), then a study specific endoscopy must be performed during the period of the Screening Visit (Visit 1) or at the Baseline Visit (Visit 2). Biopsies are not required for this endoscopy provided that a histology report with results that are compatible with UC is present in the subject's source documents. The confirmatory histology may have been at any time in the subject's history. If histologic confirmation of the previous UC diagnosis is not available in source documents, the entry endoscopy and confirmatory histology must be performed during the period of the Screening Visit (Visit 1) as for those subjects without a confirmed diagnosis of UC.

Subjects who enter the Double-blind Maintenance Phase directly:

All subjects must have a study endoscopy (flexible sigmoidoscopy or colonoscopy) performed during the period of the Screening Visit (Visit 1) or at the Baseline Visit (Visit 2). Biopsies are not required for this endoscopy provided that a histology report with results that are compatible with UC is present in the subject's source documents. The confirmatory histology may have been at any time in the subject's history. If histologic confirmation of the previous UC diagnosis is not available in source documents, the entry endoscopy and confirmatory histology must be performed during the period of the Screening Visit (Visit 1) as for those subjects without a confirmed diagnosis of UC.

All subjects:

Endoscopic video or photographs will be collected and sent to the study's central endoscopy reader for evaluation of mucosal appearance.

Mucosal appearance (endoscopy score) entry criterion should be assessed based on the central reader score, if available. Only in the event that the central reader score is not available at the time of the Baseline Visit (Visit 2) should the local reading be used. However, the central reader's score will be used for the calculation of the full UC-DAI score.

Please see a description of criteria used for endoscopic scoring in Appendix 2.

Stool collection (for eligibility assessments, [redacted]) may occur at any time during the period of the Screening Visit (Visit 1), provided that written informed consent/assent has been collected before this procedure. If a standard of care stool sample has been collected within 24 hours prior to the Screening Visit (Visit 1), this sample may be used for screening assessments and evaluations provided that written informed consent/assent has been collected before the stool is sent for laboratory analysis. Stool samples collected for culture may be processed by local laboratories; however, results from the central laboratory analysis will prevail and will be entered into the eCRF.

Subjects with positive stool cultures for any enteric pathogen (see Section 4.2) will not be eligible for the study.

Results for eligibility assessments must be available and evaluated prior to the end of the period of the Screening Visit (Visit 1) and prior to randomization at the Baseline Visit (Visit 2).

Instructions relative to the use of the e-diary for UC-DAI symptoms (rectal bleeding and stool frequency), the DUCS, and Overall Current Health entry will be reviewed with the subject and/or caregiver, and a training diary entry must be completed prior to live data entry. Starting from the first evening of the period of the Screening Visit (Visit 1), each subject and/or caregiver will be instructed to complete the e-diary every night before bedtime during the period of the Screening Visit (Visit 1). It is recommended that site staff telephone the subject or the subject's caregiver approximately 4 to 7 days prior to each visit to remind them to answer the questions in the e-diary every night, even if they have no symptoms. It is of particular importance to remind the subject at least 3 days prior to the Baseline Visit (Visit 2) to complete the questions in the e-diary as this information is required to assess the eligibility of the subject.

A screen failure is a subject who has given informed consent and failed to meet the inclusion and/or met at least 1 of the exclusion criteria and has not been randomized or administered investigational product(s). Subjects who do not meet all the eligibility criteria for either the Double-blind Acute Phase or the Double-blind Maintenance Phase or who do not have symptom score data available (stool frequency and rectal bleeding), (see Section 7.2.1.1 for details), will be considered screen failures. Subjects may be rescreened after consultation with the medical monitor.

7.1.1.2 Recommended Telephone Call (Day –7 to –4)

Approximately 4 to 7 days prior to the Baseline Visit (Visit 2), it is recommended that site staff telephone the subject or the subject's caregiver to remind them to enter UC-DAI symptoms (rectal bleeding and stool frequency) into the e-diary every night, even if the subject has no symptoms.

7.1.1.3 Baseline Visit (Visit 2)

The Baseline visit will take place 3 to 21 days after the first day of the Screening Visit (Visit 1) to allow blood and stool tests to be analyzed, and for collection of e-diary data. An extension to this 3- to 21-day period is allowed only in the case where a repeat endoscopy will be performed during the period of the Screening Visit (Visit 1) (see Section 7.1.1.1).

The ±3-day visit window which is applicable to other study visits is not applicable to this visit.

The partial UC-DAI score will be calculated by the site, and the mucosal appearance (endoscopy score) will be evaluated separately to determine eligibility for either the Double-blind Acute Phase or the Double-blind Maintenance Phase. The following criteria will determine the study phase a subject can be eligible to enter:

- Subjects with partial UC-DAI ≥2 (with a combined rectal bleeding ***and*** stool frequency score ≥1 ***and*** PGA=1 or 2) ***and*** with mucosal appearance (endoscopy score)=2 or 3 will be eligible to enter the Double-blind Acute Phase.

- Subjects with partial UC-DAI ≤1 (with rectal bleeding=0 ***and*** stool frequency ≤1 ***and*** PGA=0) ***and*** with mucosal appearance (endoscopy score)=0 or 1 will be eligible to enter the Double-blind Maintenance Phase.

Subjects with partial UC-DAI score between 1 and 2 will not be eligible to enter the Double-blind Acute Phase or the Double-blind Maintenance Phase.

Subjects who do not meet all the eligibility criteria for either the Double-blind Acute Phase or the Double-blind Maintenance Phase will be considered screen failures. Subjects may be rescreened after consultation with the medical monitor.

A minimum of 1 score each for stool frequency and rectal bleeding must be available in order to randomize subjects into either the Double-blind Acute Phase or the Double-blind Maintenance Phase (see Section 7.2.1.1 for details). Subjects with no symptom score data available will be considered screen failures as these symptom score data are used to calculate the partial UC-DAI score needed for enrollment.

Subjects with a historical diagnosis of UC:

Endoscopy (flexible sigmoidoscopy or colonoscopy) must be performed at the Baseline Visit (Visit 2) if not performed at the Screening Visit (Visit 1) or within 21 days prior to the Screening Visit (Visit 1) (21 days prior applicable only to subjects entering into the Double-blind Acute Phase directly). Endoscopic video or photographs will be collected and sent to the study's central endoscopy reader for evaluation of mucosal appearance.

Please see a description of criteria used for endoscopic scoring in Appendix 2.

If the results of blood and stool tests or partial UC-DAI score show that the subject is not eligible for the study, he/she will not continue in the study and will be considered a screen failure. No additional data will be captured for these subjects.

Eligible subjects will be randomized to low- or high-dose MMX mesalamine/mesalazine stratified by their weight group at the Baseline Visit (Visit 2). If a subject's weight changes during a study phase, their dose will not change.

Weight groups have the same absolute low and high doses for both the Double-blind Acute Phase and the Double-blind Maintenance Phase (see Section 6.2.3).

Double-blind investigational product will be dispensed to eligible subjects per the procedures detailed in Section 6.

Subjects and/or caregivers will be instructed to collect UC-DAI symptoms (rectal bleeding and stool frequency) via e-diary once a day before bedtime for 5 days prior to each study visit in the Double-blind Acute Phase and/or Double-blind Maintenance Phase as applicable. The average stool frequency and rectal bleeding scores will be calculated and be made available to the investigator through a report from the e-diary provider. The average stool frequency and rectal bleeding scores will be added to the PGA score determined by the investigator to calculate the partial UC-DAI. Please see a description of the PGA in Section 7.2.1.1 and the UC-DAI in Sections 7.2.1.1 and 7.2.1.2.

Subjects and/or caregivers will be instructed to complete the DUCS and the Overall Current Health e-diary once a day before bedtime for 5 days prior to each study visit in the Double-blind Acute Phase or Double-blind Maintenance Phase as applicable.

The IMPACT III questionnaire will be completed by children aged 8-17 years via paper at the Baseline Visit (Visit 2). Please see a description of the IMPACT III questionnaire in Section 7.2.4.1.

The PUCAI score will be calculated by a physician at the site, where applicable. Please see a description of the PUCAI in Section 7.2.4.1.

See Table 1 and Table 3 for a listing of all Baseline Visit (Visit 2) procedures.

7.1.1.4 Subject Visit Schedule Summary Post-baseline

Following the Baseline Visit (Visit 2), subjects will proceed through the study in 1 of 3 ways.

1. Double-blind Acute Phase → Open-label Acute Phase → Double-blind Maintenance Phase.

Visits completed post-Baseline Visit (Visit 2) are as follows: Visit 3, Visit 3.1, Visit 3.2, Visit 4, Visit 4.1, Visit 4.2, Visit 5, Visit 5.1, Visit 5.2, and Visit 6.

Note that Visit 3.1 and/or Visit 3.2 may not be completed in the case that a subject enters into the Open-label Acute Phase without completing the Double-blind Acute Phase. See Section 7.1.3 for the criteria for subject eligibility to enter the Open-label Acute Phase.

2. Double-blind Acute Phase → Double-blind Maintenance Phase.

Visits completed post-Baseline Visit (Visit 2) are as follows: Visit 3, Visit 3.1, Visit 3.2, Visit 5, Visit 5.1, Visit 5.2, and Visit 6.

3. Double-blind Maintenance Phase only.

Visits completed post-Baseline Visit (Visit 2) are as follows: Visit 5, Visit 5.1, Visit 5.2, and Visit 6.

7.1.2 Double-blind Acute Phase

7.1.2.1 Recommended Telephone Call (Day –7 to –4)

Approximately 4 to 7 days prior to the Baseline Visit (Visit 2) and prior to each subsequent visit (Visits 3, 3.1, and 3.2), it is recommended that site staff telephone the subject or the subject's caregiver to remind them to enter UC-DAI symptoms (rectal bleeding and stool frequency) into the e-diary every night, even if the subject has no symptoms.

7.1.2.2 Double-blind Acute Phase Weeks 2 and 4 (Visits 3 and 3.1, respectively)

Subjects and/or caregivers will collect UC-DAI symptoms (rectal bleeding and stool frequency) via e-diary and will complete the DUCS and the Overall Current Health e-diary once a day before bedtime for 5 days prior to each study visit in the Double-blind Acute Phase.

The PGA score will be determined by the investigator. Please see a description of the PGA in Section 7.2.1.1.

The Global Change in Health questionnaire will be completed via paper at Visits 3 and 3.1. Please see a description of the Global Change in Health questionnaire in Section 7.2.4.1.

See Table 1 for a listing of all Visit 3 and Visit 3.1 procedures.

7.1.2.3 Double-blind Acute Phase Week 8/Withdrawal (Visit 3.2)

The Double-blind Acute Phase Withdrawal Visit may occur at any time the subject discontinues participation in the Double-blind Acute Phase, or at completion of the study at Week 8 of the Double-blind Acute Phase. No additional Baseline Visit is required for subjects continuing into the Open-label Acute Phase or the Double-blind Maintenance Phase from this visit.

Subjects with a clinical response ie, partial UC-DAI ≤1 (with rectal bleeding=0 ***and*** stool frequency ≤1 ***and*** PGA=0) will be weighed and re-randomized into the Double-blind Maintenance Phase for treatment with low- or high-dose MMX mesalamine/mesalazine stratified by weight group based on current weight. Please see Section 6.2.3 for weight-stratified randomization groups. Subjects without a clinical response may continue into the Open-label Acute Phase and receive MMX mesalamine/mesalazine at the high dose for their weight group, based on their weight at Visit 3.2 (Week 8/Withdrawal of the Double-blind Acute Phase).

Collection of stool samples for all assessments must occur before administration of any bowel preparations for endoscopy if samples are collected on the same day as bowel preparation administration.

Endoscopy (flexible sigmoidoscopy or colonoscopy) should be performed at Visit 3.2 for subjects who are not entering into the Open-label Acute Phase. If performed, endoscopic video or photographs will be collected and sent to the study's central endoscopy reader for evaluation of mucosal healing. Please see a description of criteria used for endoscopic scoring in Appendix 2. If endoscopy is not performed at Visit 3.2 and the subject is otherwise eligible, the subject may remain in the study.

Subjects and/or caregivers will have collected UC-DAI symptoms (rectal bleeding and stool frequency) via e-diary once a day before bedtime for 5 days prior to each study visit in the Double-blind Acute Phase. The average stool frequency and rectal bleeding scores will be calculated and be made available to the investigator through a report from the e-diary provider. The average stool frequency and rectal bleeding scores will be added to the PGA score determined by the investigator to calculate the partial UC-DAI. Please see a description of the PGA in Section 7.2.1.1 and the UC-DAI in Sections 7.2.1.1 and 7.2.1.2.

The IMPACT III (for children aged 8-17 years) and Global Change in Health questionnaires will be completed via paper at Visit 3.2. Please see a description IMPACT III and Global Change in Health questionnaires in Section 7.2.4.1.

The PUCAI score will be calculated by a physician at the site, where applicable. Please see a description of the PUCAI in Section 7.2.4.1.

A pharmacokinetic sample will be taken with the biochemistry and hematology sample from subjects at sites qualified for and participating in the pharmacokinetic sampling. Please see a description of the pharmacokinetic sampling in Section 7.2.3.

See Table 1 for a listing of all Visit 3.2 procedures.

7.1.3 Open-label Acute Phase

Subjects may enter the Open-label Acute Phase after completion of the Double-blind Acute Phase if they do not meet the requirements for entry into the Double-blind Maintenance Phase ie, they do not achieve a clinical response defined as a partial UC-DAI ≤1 (with rectal bleeding=0 ***and*** stool frequency ≤1 ***and*** PGA=0). This phase is intended to allow those subjects without clinical response in the Double-blind Acute Phase an additional 8 weeks to achieve clinical response at the high dose for their weight group. The subject's weight at Visit 3.2 (Week 8/Withdrawal of the Double-blind Acute Phase) will be used to determine the high dose assigned to the subject in the Open-label Acute Phase.

Subjects are also eligible for the Open-label Acute Phase provided they have participated in the Double-blind Acute Phase for a minimum of 2 weeks and, in the investigator's opinion, have not benefited from treatment in the Double-blind Acute Phase. In addition, they should not have experienced a possibly or probably related severe adverse event (AE) or a serious AE (SAE). Due to the delay in onset of action of 5-ASA preparations, investigators should make subjects/caregivers aware that they may not benefit from the investigational product within the first 2 weeks. Subjects who withdraw before Visit 3 will not be eligible for the Open-label Acute Phase.

7.1.3.1 Recommended Telephone Call (Days –7 to –4)

Approximately 4 to 7 days prior to Visit 4 and prior to each subsequent visit (Visits 4.1 and 4.2), it is recommended that site staff telephone the subject or the subject's caregiver to remind them to enter UC-DAI symptoms (rectal bleeding and stool frequency) into the e-diary every night, even if the subject has no symptoms.

7.1.3.2 Open-label Acute Phase Weeks 2 and 4 (Visits 4 and 4.1, respectively)

Subjects and/or caregivers will collect UC-DAI symptoms (rectal bleeding and stool frequency) via e-diary and will complete the DUCS and the Overall Current Health e-diary once a day before bedtime for 5 days prior to each study visit in the Open-label Acute Phase.

See Table 2 for a listing of all Visit 4 and 4.1 procedures.

7.1.3.3 Open-label Acute Phase Week 8/Withdrawal (Visit 4.2)

The Open-label Acute Phase Withdrawal Visit may occur at any time the subject discontinues participation in the Open-label Acute Phase, or at completion of the study at Week 8 of the Open-label Acute Phase. No additional Baseline Visit is required for subjects continuing into the Double-blind Maintenance Phase from this visit.

Subjects with a clinical response, ie, partial UC-DAI ≤1 (with rectal bleeding=0 ***and*** stool frequency ≤1 ***and*** PGA=0) will be weighed and re-randomized into the Double-blind Maintenance Phase of the study for treatment with low- or high-dose MMX mesalamine/mesalazine stratified by the weight group based on current weight. Please see Section 6.2.3 for weight-stratified randomization groups.

Subjects without a clinical response after completion of the Open-label Acute Phase will be withdrawn from the study at Visit 4.2. The Follow-up Visit 6 must be conducted.

Collection of stool samples for all assessments must occur before administration of any bowel preparations for endoscopy if samples are collected on the same day as bowel preparation administration.

Endoscopy (flexible sigmoidoscopy or colonoscopy) should be performed at Visit 4.2. If performed, endoscopic video or photographs will be collected and sent to the study's central endoscopy reader for evaluation of mucosal healing. Please see a description of criteria used for endoscopic scoring in Appendix 2. If endoscopy is not performed and the subject is otherwise eligible at Visit 4.2, the subject may remain in the study.

Subjects and/or caregivers will have collected UC-DAI symptoms (rectal bleeding and stool frequency) via e-diary once a day before bedtime for 5 days prior to each study visit in the Open-label Acute Phase. The average stool frequency and rectal bleeding scores will be calculated and be made available to the investigator through a report from the e-diary provider. The average stool frequency and rectal bleeding scores will be added to the PGA score determined by the investigator to calculate the partial UC-DAI. Please see a description of the PGA in Section 7.2.1.1 and the UC-DAI in Sections 7.2.1.1 and 7.2.1.2.

The PUCAI score will be calculated by a physician at the site, where applicable. Please see a description of the PUCAI in Section 7.2.4.1.

A pharmacokinetic sample will be taken with the biochemistry and hematology sample from subjects at sites qualified for and participating in the pharmacokinetic sampling. Please see a description of the pharmacokinetic sampling in Section 7.2.3.

See Table 2 for a listing of all Visit 4.2 procedures.

7.1.4 Double-blind Maintenance Phase

7.1.4.1 Recommended Telephone Call (Days –7 to –4)

Approximately 4 to 7 days prior to the Baseline Visit (Visit 2) and prior to each subsequent visit (Visits 5, 5.1, and 5.2), it is recommended that site staff telephone the subject or the subject's caregiver to remind them to enter UC-DAI symptoms (rectal bleeding and stool frequency) into the e-diary every night, even if the subject has no symptoms.

7.1.4.2 Double-blind Maintenance Phase Weeks 2-4 (Visit 5)

Subjects and/or caregivers will return to the study site once between 2-4 weeks after randomization into the Double-blind Maintenance Phase.

The purpose of this visit is to dispense additional investigational product. Unused investigational product and empty/used investigational product packaging will be collected.

The subject does not need to be present for this visit; it is sufficient for the caregiver only to return to the study site for investigational product dispensation and collection procedures.

No study procedures other than investigational production dispensation and collection are performed at Visit 5; however, if information is offered by the subject or caregiver that indicates an AE has occurred, the relevant information will be collected and followed up as described in Section 8.

See Table 3 for a listing of all Visit 5 procedures.

7.1.4.3 Double-blind Maintenance Phase Week 13 (Visit 5.1)

Subjects and/or caregivers will collect UC-DAI symptoms (rectal bleeding and stool frequency) via e-diary and will complete the DUCS and the Overall Current Health e-diary once a day before bedtime for 5 days prior to each study visit in the Double-blind Maintenance Phase except for Visit 5, which includes collection and dispensation of investigational product only.

See Table 3 for a listing of all Visit 5.1 procedures.

7.1.4.4 Double-blind Maintenance Phase Week 26/Withdrawal (Visit 5.2)

The Double-blind Maintenance Phase Withdrawal Visit may occur at any time the subject discontinues participation in the Double-blind Maintenance Phase, or at completion of the study at Week 26 of the Double-blind Maintenance Phase.

Collection of stool samples for all assessments must occur before administration of any bowel preparations for endoscopy if samples are collected on the same day as bowel preparation administration.

Endoscopy (flexible sigmoidoscopy or colonoscopy) should be performed at Visit 5.2 where possible. If performed, endoscopic videos or photographs will be collected and sent to the study's central endoscopy reader for evaluation of mucosal healing. Please see a description of criteria used for endoscopic scoring in Appendix 2.

Subjects and/or caregivers will have collected UC-DAI symptoms (rectal bleeding and stool frequency) via e-diary once a day before bedtime for 5 days prior to each study visit in the Double-blind Maintenance Phase except for Visit 5, which includes collection and dispensation of investigational product only. The average stool frequency and rectal bleeding scores will be calculated and made available to the investigator through a report from the e-diary provider. The average stool frequency and rectal bleeding scores will be added to the PGA score determined by the investigator to calculate the partial UC-DAI. Please see a description of the PGA in Section 7.2.1.1 and the UC-DAI in Sections 7.2.1.1 and 7.2.1.2.

The IMPACT III (for children aged 8-17 years) and Global Change in Health questionnaires will be completed via paper at Visit 5.2. Please see a description IMPACT III and Global Change in Health questionnaires in Section 7.2.4.1.

The PUCAI score will be calculated by a physician at the site, where applicable. Please see a description of the PUCAI in Section 7.2.4.1.

A pharmacokinetic sample will be taken with the biochemistry and hematology sample from subjects at sites qualified for and participating in the pharmacokinetic sampling. Please see a description of the pharmacokinetic sampling in Section 7.2.3.

See Table 3 for a listing of all Visit 5.2 procedures.

7.1.5 Follow-up Period (Visit 6)

The follow-up period for this protocol is 7 days following last dose of investigational product. All subjects, whether they have completed the study or withdrawn early, must have a follow-up contact.

At the end of this period there will be a visit or telephone call initiated by the site staff to query for SAEs, AEs, and concomitant treatments. All AEs and SAEs that are not resolved at the time of this contact will be followed to closure (see Section 8).

7.1.6 Additional Care of Subjects After the Study

No additional care is planned after a subject's participation in this study unless required by local laws, ethics committees or competent authorities. The Principal Investigator at each site will be responsible for oversight of his or her study participant who continues on MMX mesalamine/mesalazine after study completion as a part of the aftercare program.

7.2 Study Evaluations and Procedures

7.2.1 Efficacy

The primary efficacy assessment of the Double-blind Acute Phase is the proportion of subjects with a clinical response (defined as partial UC-DAI ≤1 with rectal bleeding=0 ***and*** stool frequency ≤1 ***and*** PGA=0) at Week 8. The primary efficacy assessment of the Double-blind Maintenance Phase is the proportion of subjects with a clinical response at Week 26.

A secondary efficacy assessment of the Double-blind Acute Phase is the proportion of subjects with a clinical and endoscopic response at Week 8 defined as full UC-DAI ≤2 with rectal bleeding=0 ***and*** stool frequency ≤1 ***and*** PGA=0, ***and*** with mucosal healing (endoscopy score ≤1) based on central reading. In addition, there must be at least a 1-point reduction in endoscopy score from baseline. A secondary efficacy assessment of the Double-blind Maintenance Phase is the proportion of subjects with a clinical and endoscopic response at Week 26.

Further secondary assessments will explore the change in the DUCS and the percentage of subjects with an improvement in PUCAI from baseline to the end of the Double-blind Acute Phase. The change in DUCS will be explored from Week 0 to Week 26 of the Double-blind Maintenance Phase, and the percentage of subjects in remission using the PUCAI score at Week 26 of the Double-blind Maintenance Phase will also be explored.

[redacted]

7.2.1.1 Partial UC-DAI Score

The partial UC-DAI score is based on the individual parameters of the modified full UC-DAI without the endoscopic component. (Please see Section 7.2.1.2 for a description of the modified full UC-DAI.) It includes stool frequency, rectal bleeding, and PGA only. All 3 parameters will be assessed individually on a scale from 0-3; the maximum total partial UC-DAI score is 9.0.

Subjects' symptoms (rectal bleeding and stool frequency) will be reported by the subjects/caregivers in the e-diary once a day before bedtime starting from the evening of the Screening Visit (Visit 1) through the Baseline Visit (Visit 2). Subjects' symptoms are completed once a day before bedtime for the 5 days immediately prior to Visits 3, 3.1, and 3.2 of the Double-blind Acute Phase, Visits 4, 4.1, 4.2 of the Open-label Acute Phase and Visits 5.1 and 5.2 of the Double-blind Maintenance Phase. Symptom data should also be reported as soon as a subject's symptoms suggest that they might be experiencing an acute flare during the Double-blind Maintenance Phase, and they should contact the site to arrange for an unscheduled visit. In this case, symptoms should be entered for 5 days immediately prior to the unscheduled visit, if possible.

Stool frequency and rectal bleeding will be assessed on a scale from 0-3. Scores will be based on 1 of 2 versions of e-diary questions. One version is for children and adolescents aged 11-17 years and the other is for caregivers of children aged 5-10 years. Subject age at the beginning of their current study phase will determine which version of the e-diary will be completed for the entire phase; the version being completed will not change from caregiver to subject completion during a phase. However, the version should change from caregiver completion to subject completion for the next phase, if applicable based on a subject's age change.

Questions and responses for children and adolescents aged 11-17 years:

How many more times did you poop than you normally do since you went to bed last night?

- I didn't poop more than I normally do
- I pooped 1 or 2 more times than normal
- I pooped 3 or 4 more times than normal
- I pooped 5 or more times than normal.

Did you have blood in your poop?

- I didn't see any blood in my poop
- I saw streaks of blood (a small amount) in my poop
- I saw some blood (more than a small amount) in my poop
- I saw mostly blood (a lot) in my poop.

Questions and responses for caregivers of children aged 5-10 years:

Based on what you observed, how many more times did your child have a bowel movement than they normally do since they went to bed last night?

- They didn't have more bowel movements than they normally do
- They had 1 or 2 more bowel movements than normal
- They had 3 or 4 more bowel movements than normal
- They had 5 or more bowel movements than normal.

Based on what you observed, did your child have blood in their stool?

- I didn't see any blood in my child's stool
- I saw streaks of blood (a small amount) in my child's stool
- I saw some blood (more than a small amount) in my child's stool
- I saw mostly blood (a lot) in my child's stool.

For determination of the partial UC-DAI, the average of the symptom scores of the last available 3 days within the 5-day period immediately prior to the study visit will be used. No symptom data older than 5 days prior to the study visit will be used. Data collected on days when bowel preparations have been administered or when endoscopy has been performed will be excluded.

Approximately 4 to 7 days prior to the Baseline Visit (Visit 2) and prior to each subsequent visit, it is recommended that site staff telephone the subject or the subject's caregiver to remind them to enter UC-DAI symptoms (rectal bleeding and stool frequency) into the e-diary every night, even if the subject has no symptoms. A missed day's data may be entered until 12:00 hours the following day.

A minimum of 1 score each for stool frequency and rectal bleeding must be available in order to randomize subjects into either the Double-blind Acute Phase or the Double-blind Maintenance Phase. If no data are available, the average value will be considered missing and the subject will be considered a screen failure.

Calculations of average stool frequency and rectal bleeding scores will proceed as follows:

- If symptom scores from the last 3 days are available, average = (x1+x2+x3)/3; the average value should be rounded up to 1 decimal place:
  - For example: (0+1+0)/3 = 0.3333; value = 0.3; (1+0+1)/3=0.666666; value=0.7; (2+2+2)/3=2.0
- If symptom scores from the last 2 days are available, average = (x1+x2)/2; the average value should be rounded up to 1 decimal place.
- If symptom scores from only 1 day are available, average = (x1/1) = x1.

The PGA will be performed at all visits where the partial UC-DAI score is calculated by the study site, and is scored on a scale from 0-3, where 0=no active disease, 1=mild disease, 2=moderate disease, and 3=severe disease. The PGA should be performed by the same investigator at all visits, if possible, for consistency in evaluation.

Scoring of the PGA by the investigator should take into account the following parameters:

- Rectal bleeding
- Stool frequency and consistency
- Night-time bowel movements
- Abdominal pain
- Impact to daily activities
- Physical findings
- Endoscopic findings (if endoscopy is performed).

The PGA is to be recorded in source documentation and added to the average stool frequency and average rectal bleeding scores to calculate the partial UC-DAI score. Sites should not perform rounding of partial UC-DAI scores. The average stool frequency and rectal bleeding scores will be calculated and available to the investigator through a report from the e-diary provider.

7.2.1.2 Modified Full UC-DAI Score

The full UC-DAI is widely used to assess treatment efficacy in subjects with mild to moderate UC (Schroeder et al. 1987; Sutherland et al. 1987). It consists of 4 individual parameters: stool frequency, rectal bleeding, endoscopy score (mucosal appearance), and PGA. For the purpose of this study, the standard UC-DAI scale has been modified so that an endoscopy score of mild disease does not include friability; instead, friability will be scored as 2: moderate disease.

Please see a description of stool frequency, rectal bleeding and PGA in Section 7.2.1.1.

All 4 parameters will be assessed individually on a scale from 0-3; the maximum total modified full UC-DAI score is 12.0. The modified full UC-DAI score will be calculated at Baseline (Visit 2), Week 8 of the Double-blind Acute Phase (Withdrawal) for subjects not entering into the Open-label Acute Phase, Week 8 of the Open-label Acute Phase (Withdrawal) for subjects who have not had endoscopy at the end of the Double-blind Acute Phase, and Week 26 of the Double-blind Maintenance Phase (Withdrawal) visits. The total score will be calculated by summing the individual scores for the 4 parameters (endoscopy score will be based on the central reader's score). The site is not required to calculate the full UC-DAI; this will be calculated centrally using e-diary and eCRF data.

For determination of the modified full UC-DAI, the average of the symptom scores of the last available 3 days within the 5-day period immediately prior to study visits where endoscopic data are available, will be used. No symptom data older than 5 days prior to the study visit will be used. Data collected on days when bowel preparations have been administered or when endoscopy has been performed will be excluded.

An endoscopy must be performed and mucosal appearance scored within 21 days prior to the Screening Visit (Visit 1), during the Screening period or at the Baseline Visit (Visit 2) depending on the subject's UC history and phase of the study into which they are entering. All efforts should be made to perform endoscopy at Week 8 of the Double-blind Acute Phase (or Week 8 of the Open-label Acute Phase, as applicable), Week 26 of the Double-blind Maintenance Phase, or the subject's withdrawal if different from these visits.

During this study, an attempt will be made to collect endoscopy data in approximately 30% of subjects at the end of the Acute Phase (combined Double-blind and Open-label) and approximately 30% at the end of the Double-blind Maintenance Phase. In addition, data from endoscopies performed as a part of routine clinical care during a subject's participation in the study will be collected.

Endoscopies will be centrally read and scored for this study, and endoscopic video or photographs must be provided to the central reader for each endoscopy performed. Video images are preferred; however, photographic images will be accepted when video images are not of sufficient quality, as determined by the central reader during individual site image evaluation prior to enrollment at the site. Further details of requirements for video and photographic images will be provided to study sites under separate cover by the central reader. All endoscopies should be performed by the same investigator/endoscopist, if possible, to ensure consistency in endoscopy.

Please see Appendix 2 for the modified full UC-DAI.

7.2.2 Safety

The name and address of each third party vendor (eg, clinical laboratory) used in this study will be maintained in the investigator's and sponsor's files.

7.2.2.1 Medical and Medication History

At screening, the subject's medical history (including UC history) and concomitant medications will be reviewed. Investigator site staff will conduct this review via interview with the subject and/or caregiver to determine eligibility. Any new medical events or changes in intensity from the Screening Visit (Visit 1) noted during the study should be evaluated for report as an AE (see Section 8). New medications or changes to existing medications must be added or updated as appropriate.

7.2.2.2 Physical Examination

Abnormalities identified at the Screening Visit (Visit 1) will be documented in the subject's source documents. Changes after the Screening Visit (Visit 1) will be captured as AEs, as deemed appropriate by the investigator.

7.2.2.3 Height and Weight

Height will be assessed at the Screening Visit (Visit 1) and at the final visit of each phase. Weight will be assessed at the Screening Visit (Visit 1), the Baseline Visit (Visit 2) and at the final visit of each phase.

The collection of height and weight data should be precise. A calibrated stadiometer must be used for all height measurements. Height should be measured in stocking feet (without shoes) with the subject standing on a flat surface and with chin parallel to the floor. The body should be straight but not rigid. The subject's height should be recorded to the nearest 0.5 inch or 1 cm.

The same calibrated scale must be used for all weight measurements. Weight should be measured in stocking feet (without shoes) and recorded to the nearest 0.2 kg.

7.2.2.4 Adverse Event Collection

At each study visit, subjects will be questioned in a general way to ascertain if AEs have occurred since the previous visit (eg, "Have you had any health problems since your last visit?"). Adverse events are collected from the time informed consent is signed. (Please refer to Section 8, Adverse and Serious Adverse Events Assessment.)

In addition, any symptoms/conditions reported during assessments or collected via structured data collection will be evaluated by the investigator at each study visit to determine whether these are AEs.

7.2.2.5 Vital Signs

Vital signs include blood pressure and pulse. Blood pressure should be determined by cuff (using the same method, the same arm, and in the same position throughout the study). Any clinically significant deviations from the Baseline Visit (Visit 2) vital signs which are deemed clinically significant in the opinion of the investigator are to be recorded as an AE.

Measurements of vital signs (blood pressure and pulse) will be performed at all study visits. Blood pressure and pulse will be determined in the sitting position (after 5 minutes).

7.2.2.6 Clinical Laboratory Evaluations

All clinical laboratory assays will be performed according to the laboratory's normal procedures. Reference ranges are to be supplied by the laboratory and will be used to assess the clinical laboratory data for clinical significance and out-of-range pathological changes. The investigator should assess out-of-range clinical laboratory values for clinical significance, indicating if the value(s) is/are not clinically significant or clinically significant. Abnormal clinical laboratory values, which are unexpected or not explained by the subject's clinical condition, may, at the discretion of the investigator or sponsor, be repeated as soon as possible until confirmed, explained, or resolved.

The following routine clinical laboratory assessments will be performed (see Section 8.1.5 for additional clinical laboratory assessments that may be performed in the case of elevated liver enzymes):

**Biochemistry (approximately 5mL sample)**	
Alanine aminotransferase	Direct bilirubin
Albumin	Gamma-glutamyl transferase
Alkaline phosphatase	Glucose random, serum
Aspartate aminotransferase	Lactate dehydrogenase
Bilirubin, total	Magnesium
Bicarbonate	Phosphate
Calcium	Potassium
Chloride	Sodium
Cholesterol, total	Total protein
Creatinine, enzymatic	Triglycerides
Creatine kinase	Urea (blood urea nitrogen)
C-reactive protein standard	Uric acid
**Hematology (approximately 3mL sample)**	
Complete blood count (red blood cells, white blood cells, platelets, hemoglobin)	White blood cell differential
Hematocrit	
**Urinalysis**	
pH	Ketones
Specific gravity	Bilirubin
Protein	Blood
Glucose	

Microscopic examination will be conducted if protein and/or blood is/are detected during urinalysis. The microscopic examination will consist of red blood cells, white blood cells, casts, and bacteria.

Please see Table 1, Table 2, and Table 3 for visits requiring biochemistry, hematology and urinalysis collection.

7.2.2.7 Pregnancy Test

A serum β-HCG pregnancy test is performed for all females of childbearing potential at the following visits as applicable to the subject's participation in the study:

- Screening Visit (Visit 1)
- Double-blind Acute Phase: Week 8 (Visit 3.2)
- Open-label Acute Phase: Week 8 (Visit 4.2)
- Double-blind Maintenance Phase: Week 26 (Visit 5.2)
- If pregnancy is suspected
- On withdrawal of the subject from the study.

A urine pregnancy test is performed for all females of childbearing potential at all other visits as indicated in Table 1, Table 2, and Table 3.

7.2.2.8 Stool Assessment

Collection of stool samples for all assessments must occur before administration of any bowel preparations for endoscopy if samples are collected on the same day as bowel preparation administration.

If a standard of care stool sample has been collected within 24 hours prior to the Screening Visit (Visit 1), this sample may be used for screening assessments and evaluations provided that written informed consent/assent has been collected before the stool is sent for laboratory analysis.

Screening Assessments

A stool sample will be collected during the Screening Visit (Visit 1), which is a period of 3 to 21 days. If a standard of care stool sample was collected within 24 hours prior to the Screening Visit (Visit1), this sample may be used for screening assessments and evaluations. The sample must be collected in sufficient time to ensure results reporting within the Screening Visit (Visit 1) period Stool samples collected for culture may be processed by local laboratories; however, results from the central laboratory analysis will prevail and will be entered into the eCRF. The following parameters will be assessed:

- Culture for enteric pathogens including *Salmonella*, *Shigella*, *Yersinia*, *Aeromonas*, *Plesiomonas*, and *Campylobacter*

- Presence of *Clostridium difficile* toxin
- Microscopic examination of stool for ova and parasites.

If the stool sample is positive for any of the above parameters, the subject will not be eligible for the study (see Section 4.2).

[redacted]

7.2.3 Pharmacokinetic Procedures

7.2.3.1 Blood Sample Collection and Handling Procedures

Blood samples will be collected at the times specified in Table 1, Table 2 and Table 3 to allow measurement of plasma concentrations of 5-ASA and its major metabolite Ac-5-ASA. Pharmacokinetic sampling will occur only at participating sites that have been appropriately assessed and qualified for pharmacokinetic sampling.

The date and time of last dose of investigational product and date and time of pharmacokinetic sampling will be collected.

Venous blood samples (2mL) will be drawn by direct venipuncture into lithium heparin Vacutainer® tubes, capped and mixed by inversion (x3) and chilled immediately on crushed ice.

Blood samples (2mL/sample) may be collected at Visits 3.2/Withdrawal, 4.2/Withdrawal and 5.2/Withdrawal.

Within 15 minutes following each sample collection, the blood tubes will be centrifuged at approximately 2000 x g (15 minutes, 4ºC). The separated plasma will be decanted into 2 clearly labeled polypropylene tubes (0.4mL of plasma in the primary tube and in the backup tube) via a plastic pipette. All samples must be stored nominally at –80ºC and the freezer temperature will be controlled, monitored and recorded during the storage period until the samples are shipped to the central laboratory. The study monitor must be notified of any temperature excursions.

Plasma sample tubes for bioanalysis must be freezer safe and identified with freezer safe labels provided by the Sponsor. The labels will contain the following information:

- Study Number
- Visit (Visit 3.2/Withdrawal, Visit 4.2/Withdrawal, 5.2/Withdrawal)
- Subject Identifier
- Matrix Identifier (plasma)
- Split (primary or backup tube).

There will be up to 3 blood samples collected for pharmacokinetic bioanalysis per subject during the study.

7.2.3.2 Shipment of Plasma Pharmacokinetic Samples

All pharmacokinetic samples should be double-bagged to contain leaks and packed with a sufficient quantity of dry ice to ensure that they remain frozen for at least 48 hours to allow for delays in shipment. All applicable shipping regulations must be followed. Shipments will be sent to the central laboratory.

7.2.3.3 Plasma Drug Assay Methodology

Plasma sample analysis will be performed at York Bioanalytical Solutions according to York Bioanalytical Solutions standard operating procedures.

Pharmacokinetic samples will be analyzed at York Bioanalytical Solutions for 5-ASA and Ac-5-ASA using validated high performance liquid chromatography methods with tandem mass spectrometric detection.

Raw data will be stored in the archives at York Bioanalytical Solutions.

Some of the plasma samples may be selected and re-analyzed and the results compared for incurred sample reproducibility. Details of the incurred sample reproducibility experiments will be provided in the Bioanalytical Study Plan.

[redacted] [redacted]

[redacted]

[redacted]

[redacted]

[redacted]

[redacted]

[redacted]

7.2.4.1 Health-related Quality-of-Life Assessments

Daily Ulcerative Colitis Scale for Children and Caregivers

Shire has developed an electronic daily sign and symptom diary, the DUCS. Two versions have been developed: 1 patient-reported outcome (PRO) to be self-completed by children aged 8-17 years and 1 observer-reported outcome (ObsRO) to be completed by caregivers of children aged 5-10 years. Both the PRO and ObsRO will be completed by children aged 8-10 years; however, the ObsRO will be used for the secondary endpoint analysis. The data obtained from the PRO in this age group will provide information for future use to determine if children/adolescents can reliably complete the PRO.

The concept elicitation phase has informed the content of the diaries and provided for development of a draft item pool and draft measures. Cognitive debriefing interviews have been completed on these draft measures. This phase involved conducting interviews with UC patients and caregivers to evaluate the clarity, understanding, relevance, and comprehension of the draft PRO and ObsRO daily diaries as well as usability of the electronic device and feasibility of daily use at home.

Overall Current Health

An Overall Current Health item will be included in the e-diary. The Overall Current Health item has been tested in cognitive debriefing interviews with the DUCS.

PUCAI

The PUCAI is a physician-administered measure that focuses on 6 key signs and symptoms of UC and activity limitations (Turner et al. 2007). The PUCAI yields a total score ranging from 0-85 with higher scores being worse. Recommended cut-off scores to differentiate disease activity are <10 (remission); 11-30 (mild); 31-64 (moderate) and >65 (severe) (Turner et al. 2009). Evaluation of the construct validity of the PUCAI revealed that it was highly correlated with physician's global assessment of disease activity and PUCAI scores were highly predictive of the need for escalating medical therapy (Turner et al. 2009). See Appendix 4.

Only sites who have executed a sublicense with Shire for PUCAI use will be able to collect PUCAI data for the study.

IMPACT III

The IMPACT questionnaire is a disease-specific health-related quality-of-life questionnaire developed for use in pediatric inflammatory bowel disease through a process of patient interviews and analysis of patient responses to an item-reduction questionnaire. This is a self-administered questionnaire, and data will be collected via paper in this study.

The IMPACT questionnaire has been studied to determine its feasibility, reliability, and validity. It was found to be a valid and reliable reflection of health-related quality-of-life of older children and adolescents with both UC and Crohn's disease (Otley et al. 2002).

In this study, the IMPACT III will be used. This is the current version of the questionnaire, adapted from IMPACT II, which was developed in 2002. The original version of IMPACT was developed in 1999. IMPACT III contains 35 items in 6 domains. The Bowel Symptoms domain and the 4 matching symptom items between the IMPACT III and the DUCS will be used to evaluate convergent validity of the DUCS.

The IMPACT is valid for children and adolescents aged 9-17 years old and is completed by the subject. Children/adolescent aged 9-17 years who cannot complete the questionnaire independently, may be assisted by an adult who is not the child's guardian or relative. Children who are 8 years old may complete the IMPACT with assistance from an adult who is not the child's guardian or relative. Children under the age of 8 years old will not complete the IMPACT.

Global Change in Health

The Global Change in Health item assesses the subject's (aged 11-17 years) or caregiver's (for children aged 5-10 years) perception of change in health since starting the study and will be administered via paper in this study. This item has been tested in cognitive debriefing interviews with the DUCS.

7.2.5 Volume of Blood to be Drawn from Each Subject

Table 6: Volume of Blood to be Drawn From Each Subject

Assessment		Sample Volume (mL)	Number of Samples: Screening	Number of Samples: Double-blind Acute Phase	Number of Samples: Open-label Acute Phase	Number of Samples: Double-blind Maintenance Phase	Total Volume (mL)
Safety	Biochemistry and β-HCG[a]	5	1	1	1	1	20
	Hematology	3	1	1	1	1	12
Pharmacokinetic samples (at participating sites only)		2	0	1	1	1	6
Total mL			8	8	8	8	32-38

[a] β-HCG testing for females only.
β-HCG=beta-human chorionic gonadotropin

It is expected that approximately 32mL of blood will be drawn from subjects who participate in every phase of the study, regardless of sex and approximately an additional 6mL will be drawn from subjects participating in the pharmacokinetic section of the study. Blood sampling and volume drawn will be less for subjects who do not participate in every phase of the study, in accordance with Table 6.

Note: The amount of blood to be drawn for each assessment is an estimate. The amount of blood to be drawn may vary according to the instructions provided by the manufacturer or laboratory for an individual assessment; however, the total volume drawn over the course of the study should be approximately 32-38mL. When more than 1 blood assessment is to be done at the time point/period, if they require the same type of tube, the assessments may be combined.

8. ADVERSE AND SERIOUS ADVERSE EVENTS ASSESSMENT

8.1 Definition of Adverse Events, Period of Observation, Recording of Adverse Events

An AE is any untoward medical occurrence in a clinical investigation subject administered a pharmaceutical product and that does not necessarily have a causal relationship with this treatment. An AE can therefore be any unfavorable and unintended sign (including an abnormal laboratory finding), symptom, or disease temporally associated with the use of a medicinal (investigational) product, whether or not related to the medicinal (investigational) product (International Council for Harmonisation [ICH] Guidance E2A 1995).

All AEs are collected from the time the informed consent is signed until the defined follow-up period stated in Section 7.1.5. This includes events occurring during the screening phase of the study, regardless of whether or not investigational product is administered. Where possible, a diagnosis rather than a list of symptoms should be recorded. If a diagnosis has not been made, then each symptom should be listed individually. All AEs should be captured on the appropriate AE pages in the eCRF and in source documents. In addition to untoward AEs, unexpected benefits outside the investigational product indication should also be captured in the AE eCRF.

All AEs must be followed to closure (the subject's health has returned to his/her baseline status or all variables have returned to normal), regardless of whether the subject is still participating in the study. Closure indicates that an outcome is reached, stabilization achieved (the investigator does not expect any further improvement or worsening of the event), or the event is otherwise explained. When appropriate, medical tests and examinations are performed so that resolution of event(s) can be documented.

8.1.1 Severity Categorization

The severity of AEs must be recorded during the course of the event including the start and stop dates for each change in severity. An event that changes in severity should be captured as a new event. Worsening of pre-treatment events, after initiation of investigational product, must be recorded as new AEs (for example, if a subject experiences mild intermittent dyspepsia prior to dosing of investigational product, but the dyspepsia becomes severe and more frequent after first dose of investigational product has been administered, a new AE of severe dyspepsia [with the appropriate date of onset] is recorded in the appropriate eCRF).

The medical assessment of severity is determined by using the following definitions:

Mild: A type of AE that is usually transient and may require only minimal treatment or therapeutic intervention. The event does not generally interfere with usual activities of daily living.

Moderate: A type of AE that is usually alleviated with specific therapeutic intervention. The event interferes with usual activities of daily living, causing discomfort but poses no significant or permanent risk of harm to the research subject.

Severe: A type of AE that interrupts usual activities of daily living, or significantly affects clinical status, or may require intensive therapeutic intervention.

8.1.2 Relationship Categorization

A physician/investigator must make the assessment of relationship to investigational product for each AE. The investigator should decide whether, in his or her medical judgment, there is a reasonable possibility that the event may have been caused by the investigational product. If there is no valid reason for suggesting a relationship, then the AE should be classified as "not related". Otherwise, if there is any valid reason, even if undetermined or untested, for suspecting a possible cause-and-effect relationship between the investigational product and the occurrence of the AE, then the AE should be considered "related". The causality assessment must be documented in the source document.

The following additional guidance may be helpful:

Term	Relationship Definition
Related	The temporal relationship between the event and the administration of the investigational product is compelling and/or follows a known or suspected response pattern to that product, and the event cannot be explained by the subject's medical condition, other therapies, or accident.
Not Related	The event can be readily explained by other factors such as the subject's underlying medical condition, concomitant therapy, or accident and no plausible temporal or biologic relationship exists between the investigational product and the event.

8.1.3 Outcome Categorization

The outcome of AEs must be recorded during the course of the study in the eCRF. Outcomes are as follows:

- Fatal
- Not Recovered/Not Resolved
- Recovered/Resolved
- Recovered/Resolved With Sequelae
- Recovering/Resolving
- Unknown

8.1.4 Symptoms of the Disease Under Study

Symptoms of the disease under study (eg, episodes of diarrhea with blood and mucus, abdominal pain, and urgency) should not be classed as AEs as long as they are within the normal day-to-day fluctuation or expected progression of the disease and are part of the efficacy data to be collected in the study; however, significant worsening of the symptoms should be recorded as an AE.

8.1.5 Clinical Laboratory and Other Safety Evaluations

A change in the value of a clinical laboratory, or vital sign, assessment can represent an AE if the change is clinically relevant or if, during treatment with the investigational product, a shift of a parameter is observed from a normal value to an abnormal value, or a further worsening of an already abnormal value. When evaluating such changes, the extent of deviation from the reference range, the duration until return to the reference range, either while continuing treatment or after the end of treatment with the investigational product, and the range of variation of the respective parameter within its reference range, must be taken into consideration.

If, at the end of the treatment phase, there are abnormal clinical laboratory, or vital sign, values which were not present at the pretreatment value observed closest to the start of study treatment, further investigations should be performed until the values return to within the reference range or until a plausible explanation (eg, concomitant disease) is found for the abnormal values.

The investigator should decide, based on the above criteria and the clinical condition of a subject, whether a change in a clinical laboratory, or vital sign, parameter is clinically significant and therefore represents an AE.

Subjects with liver enzymes (alanine aminotransferase, aspartate aminotransferase, gamma-glutamyl transferase) elevated beyond twice the upper limit of normal will be further evaluated with a panel of tests assessing markers of hepatic injury, inflammation, and function. At a minimum, this panel should include the following: repeat alanine aminotransferase, aspartate aminotransferase, gamma-glutamyl transferase, total and direct bilirubin, serum albumin, lactate dehydrogenase, and prothrombin time. Consideration should be given as to whether an arterial blood sample for serum ammonia should also be obtained based on the magnitude of transaminase elevation and the subject's clinical status (such as presence of mental status changes or other findings that might suggest Reye's syndrome; the Shire Medical Monitor should be consulted prior to collection of this specimen). Subjects should be followed until their hepatic enzyme values return to normal ranges. If abnormal values persist for greater than 1 week, the Shire Medical Monitor should be contacted for discussion and agreement of additional etiology investigation.

8.1.6 Pregnancy

All pregnancies are to be reported from the time informed consent is signed until the defined follow-up period stated in Section 7.1.5.

Any report of pregnancy for any female study participant must be reported within 24 hours to the Shire Pharmacovigilance Department using the Shire Investigational and Marketed Products Pregnancy Report Form. The pregnant female study participant must be withdrawn from the study.

Every effort should be made to gather information regarding the pregnancy outcome and condition of the infant. It is the responsibility of the investigator to obtain this information within 30 calendar days after the initial notification and approximately 30 calendar days post-partum.

Pregnancy complications such as spontaneous abortion/miscarriage or congenital abnormality are considered SAEs and must be reported using the Shire Clinical Trial Serious Adverse Event Form. Note: An elective abortion is not considered an SAE.

In addition to the above, if the investigator determines that the pregnancy meets serious criteria, it must be reported as an SAE using the Shire Clinical Trial Serious Adverse Event Form as well as the Shire Investigational and Marketed Products Pregnancy Report Form. The test date of the first positive serum/urine β-HCG test or ultrasound result will determine the pregnancy onset date.

8.1.7 Abuse, Misuse, Overdose, and Medication Error

Abuse, misuse, overdose, or medication error (as defined below) must be reported to the sponsor according to the SAE reporting procedure whether or not they result in an AE/SAE as described in Section 8.2. Note: The 24-hour reporting requirement for SAEs does not apply to reports of abuse, misuse, overdose, or medication errors unless these result in an SAE.

The categories below are not mutually exclusive; the event can meet more than 1 category.

- **Abuse** – Persistent or sporadic intentional intake of investigational product when used for a non-medical purpose (eg, to alter one's state of consciousness or get high) in a manner that may be detrimental to the individual and/or society
- **Misuse** – Intentional use of investigational product other than as directed or indicated at any dose (Note: this includes a situation where the investigational product is not used as directed at the dose prescribed by the protocol)
- **Overdose** – Intentional or unintentional intake of a dose of an investigational product exceeding a pre-specified total daily dose of 4.8g of the product
- **Medication Error** – An error made in prescribing, dispensing, administration, and/or use of an investigational product. For studies, medication errors are reportable to the sponsor only as defined below.

Cases of subjects missing doses of the investigational product are not considered reportable as medication errors.

Medication errors should be collected/reported for all products under investigation.

The administration and/or use of the unassigned treatment is/are always reportable as a medication error.

The administration and/or use of an expired investigational product should be considered as a reportable medication error.

All investigational product provided to pediatric subjects should be supervised by the parent/LAR/caregiver.

8.2 Serious Adverse Event Procedures

8.2.1 Reference Safety Information

The reference for safety information for this study is the current MMX Mesalazine (Mesalamine) Investigator's Brochure which the sponsor has provided under separate cover to all investigators.

8.2.2 Reporting Procedures

All initial and follow-up SAE reports must be reported by the investigator to the Shire Pharmacovigilance Department and the CRO Pharmacovigilance Department within 24 hours of the first awareness of the event. Note: The 24-hour reporting requirement for SAEs does not apply to reports of abuse, misuse, overdose, or medication errors (see Section 8.1.7) unless they result in an SAE.

The investigator must complete, sign, and date the Shire Clinical Trial Serious Adverse Event Form and verify the accuracy of the information recorded on the form with the corresponding source documents (Note: Source documents are not to be sent unless requested) and fax or e-mail the form to the Shire Pharmacovigilance Department.

A copy of the Shire Clinical Trial Serious Adverse Event Form (and any applicable follow-up reports) must also be sent to the CRO Pharmacovigilance Department using the details specified in the emergency contact information section of the protocol.

8.2.3 Serious Adverse Event Definition

An SAE is any untoward medical occurrence (whether considered to be related to investigational product or not) that at any dose:

- Results in death.
- Is life-threatening. Note: The term "life-threatening" in the definition of "serious" refers to an event in which the subject was at risk of death at the time of the event; it does not refer to an event which hypothetically might have caused death if it was more severe.
- Requires inpatient hospitalization or prolongation of existing hospitalization. Note: Hospitalizations, which are the result of elective or previously scheduled surgery for pre-existing conditions, which have not worsened after initiation of treatment, should not be classified as SAEs. For example, an admission for a previously scheduled ventral hernia repair would not be classified as an SAE; however, complication(s) resulting from a hospitalization for an elective or previously scheduled surgery that meet(s) serious criteria must be reported as SAE(s).
- Results in persistent or significant disability/incapacity.
- Is a congenital abnormality/birth defect.
- Is an important medical event. Note: Important medical events that may not result in death, be life-threatening, or require hospitalization may be considered an SAE when, based upon appropriate medical judgment, they may jeopardize the subject and may require medical or surgical intervention to prevent 1 of the outcomes listed in this definition. Examples of such medical events include allergic bronchospasm requiring intensive treatment in an emergency room or at home; blood dyscrasias or convulsions that do not result in inpatient hospitalization; or the development of drug dependency or drug abuse.

8.2.4 Serious Adverse Event Collection Timeframe

All SAEs (regardless of relationship to study) are collected from the time the subject signs the informed consent until the defined follow-up period stated in Section 7.1.5, and must be reported to the Shire Pharmacovigilance Department within 24 hours of the first awareness of the event.

In addition, any SAE(s) considered "related" to the investigational product and discovered by the investigator at any interval after the study has completed must be reported to the Shire Pharmacovigilance Department within 24 hours of the first awareness of the event.

8.2.5 Serious Adverse Event Onset and Resolution Dates

The onset date of the SAE is defined as the date the event meets serious criteria. The resolution date is the date the event no longer meets serious criteria, the date the symptoms resolve, or the event is considered chronic. In the case of hospitalizations, the hospital admission and discharge dates are considered the onset and resolution dates, respectively.

In addition, any signs or symptoms experienced by the subject after signing the informed consent form, or leading up to the onset date of the SAE, or following the resolution date of the SAE, must be recorded as an AE, if appropriate.

8.2.6 Fatal Outcome

Any SAE that results in the subject's death (ie, the SAE was noted as the primary cause of death) must have fatal checked as an outcome with the date of death recorded as the resolution date. For all other events ongoing at the time of death that did not contribute to the subject's death, the outcome should be considered not resolved, without a resolution date recorded.

For any SAEs that result in the subject's death or any ongoing events at the time of death, the action taken with the investigational product should be recorded as "dose not changed" or "not applicable" (if the subject never received investigational product).

8.2.7 Regulatory Agency, Institutional Review Board, Ethics Committee, and Site Reporting

The sponsor and the clinical CRO are responsible for notifying the relevant regulatory authorities/US central institutional review boards (IRBs)/European Union (EU) central ethics committees (ECs) of related, unexpected SAEs.

In addition the clinical CRO is responsible for notifying active sites of all related, unexpected SAEs occurring during all interventional studies across the SPD476 program.

The investigator is responsible for notifying the local IRB, local EC, or the relevant local regulatory authority of all SAEs that occur at his or her site as required.

9. DATA MANAGEMENT AND STATISTICAL METHODS

9.1 Data Collection

The investigators' authorized site personnel must enter the information required by the protocol in the eCRF. A study monitor will visit each site in accordance with the monitoring plan and review the eCRF data against the source data for completeness and accuracy. Discrepancies between source data and data entered in the eCRF will be addressed by qualified site personnel. When a data discrepancy warrants correction, the correction will be made by authorized site personnel. Data collection procedures will be discussed with the site at the site initiation visit and/or at the investigator's meeting. Once a subject is randomized, it is expected that site personnel will complete the eCRF entry within approximately 3 business days of the subject's visit.

9.2 Clinical Data Management

Data are to be entered into a clinical database as specified in the CRO's data management plan. Quality control and data validation procedures are applied to ensure the validity and accuracy of the clinical database.

Data are to be reviewed and checked for omissions, errors, and values requiring further clarification using computerized and manual procedures. Data queries requiring clarification are to be communicated to the site for resolution. Only authorized personnel will make corrections to the clinical database, and all corrections are documented in an auditable manner.

9.3 Data Handling Considerations

Data that may potentially unblind the treatment assignment (ie, investigational product serum concentrations, treatment allocation, and investigational product preparation/accountability data) will be handled with special care during the data cleaning and review process. These data will be handled in such a way that, prior to unblinding, any data that may unblind study team personnel will be presented as blinded information or otherwise will not be made available. If applicable, unblinded data may be made available to quality assurance representatives for the purposes of conducting independent drug audits.

9.4 Statistical Analysis Process

The study will be analyzed by the sponsor or its agent. All data analyses will be performed by the Biometrics Department of the CRO after the study is completed and unblinded, and the database is released for analysis.

The statistical analysis plan (SAP) will provide the statistical methods and definitions for the analysis of the efficacy and safety data, as well as describe the approaches to be taken for summarizing other study information such as subject disposition, demographics and baseline characteristics, investigational product exposure, and prior and concomitant medications. The SAP will also include a description of how missing, unused, and spurious data will be addressed.

The SAP will be finalized prior to unblinding to preserve the integrity of the statistical analysis and study conclusions.

All statistical analyses will be performed using SAS® Version 8.2 (SAS Institute, Cary, NC 27513) or later version.

9.5 Planned Interim Analysis, Adaptive Design, and Data Monitoring Committee

An independent Data Monitoring Committee (DMC) will be established for this study. The purpose of the DMC is to review safety data (eg, AEs, clinical laboratory data). DMC meetings will be held when data is available from specified percentages of subjects who have completed or withdrawn from a phase or phases during the study. The data provided to the DMC will not be considered “clean” until the database is locked. Data will be provided to the DMC by an independent statistical reporting group not assigned to the project. Further details regarding the DMC and meeting frequency can be found in the DMC charter.

9.6 Sample Size Calculation and Power Considerations

This will be an estimation study with no formal hypothesis testing; therefore, this study is not powered to detect differences between treatment groups. More than 100 subjects will be screened and up to 80 subjects will be enrolled in the Double-blind Acute Phase of the study. More than 65 subjects will be screened (in addition to an expected 28 subjects who will enter the Double-blind Maintenance Phase from one of the Acute Phases) and at least 80 subjects will be enrolled in the Double-blind Maintenance Phase of the study. The sample size was chosen based on practical considerations and on agreement with the US FDA.

9.7 Study Population

The **Screened Set** will consist of all subjects who have signed an informed consent.

The Double-blind Acute Phase Safety Analysis Set will consist of randomized subjects who have taken at least 1 dose of investigational product during the Double-blind Acute Phase.

The Open-label Acute Phase Safety Analysis Set will consist of all subjects who have taken at least 1 dose of investigational product during the Open-label Acute Phase.

The Double-blind Maintenance Phase Safety Analysis Set will consist of randomized subjects who have taken at least 1 dose of investigational product during the Double-blind Maintenance Phase.

9.8 Efficacy Analyses

Baseline is defined as the last observation prior to first dose of investigational product in the study. The Double-blind Acute Phase Week 8/Withdrawal visit (Visit 3.2) is treated as the Open-label Acute Phase Week 0 for subjects continuing into the Open-label Acute Phase from this visit. The Double-blind Acute Phase Week 8/Withdrawal visit (Visit 3.2) is treated as the Double-blind Maintenance Phase Week 0 for subjects continuing into the Double-blind Maintenance Phase from this visit. The Open-label Acute Phase Week 8/Withdrawal visit (Visit 4.2) is treated as the Double-blind Maintenance Phase Week 0 for subjects continuing into the Double-blind Maintenance Phase from this visit. For subjects who enter the Double-blind Maintenance Phase directly, the Baseline Visit (Visit 2) is treated as the Double-blind Maintenance Phase Week 0.

9.8.1 Primary Efficacy Endpoint

The primary endpoints for this study are defined separately for both the Double-blind Acute Phase and Double-blind Maintenance Phases and will be conducted using the appropriate Safety Analysis Set for the Phase.

9.8.1.1 Double-blind Acute Phase

The primary efficacy endpoint for the Double-blind Acute Phase is defined as the proportion of subjects with a clinical response (defined as partial UC-DAI≤1 with rectal bleeding=0 ***and*** stool frequency ≤1 ***and*** PGA=0) at Week 8.

Subjects with missing data at Week 8 will be assumed not to have had a clinical response. The primary efficacy endpoint will be compared between treatment arms using a continuity-corrected chi-squared test on the Double-blind Acute Phase Safety Analysis Set. The null hypothesis to be tested is that there is no difference in the proportion of subjects with a clinical response at Week 8 between low and high doses of MMX mesalamine/mesalazine.

9.8.1.2 Double-blind Maintenance Phase

The primary efficacy endpoint for the Double-blind Maintenance Phase is defined as the proportion of subjects who have maintained a clinical response (defined as partial UC-DAI≤1 with rectal bleeding=0 ***and*** stool frequency ≤1 ***and*** PGA=0) at Week 26.

Subjects with missing data at Week 26 will be assumed not to have had a clinical response. The primary efficacy endpoint will be compared between treatment arms using a Cochran-Mantel-Haenszel (CMH) test stratifying for 3 levels of responder status at Week 26 of the Double-blind Maintenance Phase (entered Maintenance Phase directly, responder at Week 8 of the Double-blind Acute Phase, or responder at Week 8 of the Open-label Acute Phase) on the Double-blind Maintenance Phase Safety Analysis Set. The null hypothesis to be tested is that there is no difference in the proportion of subjects with a clinical response at Week 26 between low and high doses of MMX mesalamine/mesalazine.

9.8.2 Sensitivity Analyses of the Primary Endpoint

The following sensitivity analyses will be performed for both the Double-blind Acute and Double-blind Maintenance Phase primary endpoints:

- A modified clinical response, defined as partial UC-DAI≤1 with rectal bleeding=0 ***and*** stool frequency ≤1 will be analyzed in a similar way to the primary endpoint analysis in order to investigate the effect of removing the PGA component from the responder definition.
- An observed-case analysis will be performed, where data from those who completed Week 26 and those who withdrew from the study early and are presented and analyzed separately, using a similar analysis to the primary endpoint.
- A complete-case analysis, where subjects who withdraw early from the study will be excluded, will be performed in a similar way to the primary endpoint analysis.
- A last observation carried forward analysis, using data from the final on-treatment assessment, will be performed in a similar way to the primary endpoint analysis.
- Logistic modeling will be conducted to investigate the effect of doses in mg/kg on the clinical response in the Double-blind Acute and Double-blind Maintenance Phases.

9.8.3 Secondary Efficacy Endpoints

The secondary endpoints for this study are defined separately for both the Double-blind Acute and Double-blind Maintenance Phases and will be conducted using the appropriate Safety Analysis Set for the Phase. These endpoints will be described using appropriate summary statistics (n [%]) for categorical variables, n, mean, standard deviation, median, minimum and maximum for continuous variables).

9.8.3.1 Double-blind Acute Phase

- The proportion of subjects with a clinical and endoscopic response at Week 8, defined as UC-DAI ≤2 with rectal bleeding=0 ***and*** stool frequency ≤1 ***and*** PGA=0, ***and*** with mucosal healing (endoscopy score ≤1) based on central reading. In addition, there must be at least a 1-point reduction in endoscopy score from baseline. This endpoint will be compared between treatment arms using a continuity-corrected chi-squared test.
- The proportion of subjects with a clinical and endoscopic response at Week 8, defined as UC-DAI ≤2 with rectal bleeding=0 ***and*** stool frequency ≤1 ***and*** PGA=0, ***and*** with mucosal healing (endoscopy score ≤1) based on local reading. In addition, there must be at least a 1-point reduction in endoscopy score from baseline. This endpoint will be compared between treatment arms using a continuity-corrected chi-squared test.
- The change in the DUCS score from baseline to Week 8 of the Double-blind Acute Phase. This endpoint will be compared between treatment arms using an analysis of covariance, including the baseline DUCS score as a covariate in the model.
- The percentage of subjects with an improvement (change of ≥20 points) in PUCAI score from baseline to Week 8 of the Double-blind Acute Phase. This endpoint will be compared between treatment arms using a continuity-corrected chi-squared test.

9.8.3.2 Double-blind Maintenance Phase

- The proportion of subjects who have maintained a clinical and endoscopic response at Week 26, defined as UC-DAI ≤2 with rectal bleeding=0 ***and*** stool frequency ≤1 ***and*** PGA=0 ***and*** with mucosal healing (endoscopy score ≤1) based on central reading. This endpoint will be compared between treatment arms using a CMH test stratifying by Week 8 responder status.
- The proportion of subjects who have maintained a clinical and endoscopic response at Week 26, defined as UC-DAI ≤2 with rectal bleeding=0 ***and*** stool frequency ≤1 ***and*** PGA=0, ***and*** with mucosal healing (endoscopy score ≤1) based on local reading. This endpoint will be compared between treatment arms using a CMH test stratifying by Week 8 responder status.
- The change in the DUCS score from Double-blind Maintenance Phase Week 0 to Week 26. This endpoint will be compared between treatment arms using an analysis of covariance, including the DUCS score at Double-blind Maintenance Phase Week 0 and Week 8 responder status as covariates in the model.
- The percentage of subjects in remission (PUCAI <10) at Double-blind Maintenance Phase Week 26. This endpoint will be compared between treatment arms using a CMH test stratifying by Week 8 responder status.

9.8.4 Exploratory Efficacy Endpoints

- [redacted]
- [redacted]

9.8.5 Exploratory Pharmacokinetic Endpoint

- [redacted]

9.9 Safety Analyses

Baseline is defined as the last observation prior to first dose of investigational product in the study. The Double-blind Acute Phase Week 8/Withdrawal visit (Visit 3.2) is treated as the Open-label Acute Phase Week 0 for subjects continuing into the Open-label Acute Phase from this visit. The Double-blind Acute Phase Week 8/Withdrawal visit (Visit 3.2) is treated as the Double-blind Maintenance Phase Week 0 for subjects continuing into the Double-blind Maintenance Phase from this visit. The Open-label Acute Phase Week 8/Withdrawal visit (Visit 4.2) is treated as the Double-blind Maintenance Phase Week 0 for subjects continuing into the Double-blind Maintenance Phase from this visit. For subjects who enter the Double-blind Maintenance Phase directly, the Baseline Visit (Visit 2) is treated as the Double-blind Maintenance Phase Week 0.

All summaries of safety data will be produced separately for the Double-blind Acute, Open-label Acute, and Double-blind Maintenance Phase Safety Analysis Sets.

Adverse events will be coded using the Medical Dictionary for Regulatory Activities. The number of events, incidence, and percentage of TEAEs will be calculated overall, by system organ class, by preferred term, and by treatment group (ie, low or high dose). Treatment-emergent AEs will be defined in the SAP. Treatment-emergent AEs will be further summarized by severity and relationship to investigational product. Treatment-emergent AEs related to investigational product, TEAEs leading to withdrawal, SAEs, and deaths will be summarized/listed.

Clinical laboratory tests and vital signs at each time point and their change from baseline in the Double-blind Acute Phase or change from Open-label Acute Phase Week 0, or change from Double-blind Maintenance Phase Week 0 will be summarized appropriately.

In general, continuous variables will be summarized using number of observations, mean, standard deviation, median, minimum, and maximum values and categorical data will be summarized using number of observations and percentages. Other safety parameters will be summarized appropriately at each time point.

The number of withdrawals from the study, and the reasons for withdrawal, will be summarized by treatment arm and overall.

Narratives will be presented for all deaths, SAEs, and subjects withdrawn due to AEs.

9.10 Other Analyses

9.10.1 Health-related Quality-of-Life Analyses

In this study the final validation steps for the PRO instrument, the DUCS, will be performed. The psychometric properties of the DUCS, including reliability, validity, and responsiveness, will be examined using data from an appropriate number of subjects from the Double-blind Acute Phase and an appropriate number of subjects from the Double-blind Maintenance Phase prior to unblinding. To examine convergent validity, the DUCS scores will be correlated with the IMPACT III, Overall Current Health and selected clinical data.

The validation of the DUCS will be described in a separate SAP specific for the DUCS.

10. SPONSOR'S AND INVESTIGATOR'S RESPONSIBILITIES

This study is conducted in accordance with current applicable regulations, ICH, EU Directive 2001/20/EC and its updates, and local ethical and legal requirements.

The name and address of each third party vendor (eg, CRO) used in this study will be maintained in the investigator's and sponsor's files, as appropriate.

10.1 Sponsor's Responsibilities

10.1.1 Good Clinical Practice Compliance

The study sponsor and any third party to whom aspects of the study management or monitoring have been delegated will undertake their assigned roles for this study in compliance with all applicable industry regulations, ICH Good Clinical Practice (GCP) Guideline E6 (1996), EU Directive 2001/20/EC, as well as all applicable national and local laws and regulations.

Visits to sites are conducted by representatives of the study sponsor and/or the company organizing/managing the research on behalf of the sponsor to inspect study data, subjects' medical records, and eCRFs in accordance with current GCP and the respective local and (inter)national government regulations and guidelines. Records and data may additionally be reviewed by auditors or by regulatory authorities.

The sponsor ensures that local regulatory authority requirements are met before the start of the study. The sponsor (or a nominated designee) is responsible for the preparation, submission, and confirmation of receipt of any regulatory authority approvals required prior to release of investigational product for shipment to the site.

10.1.2 Indemnity/Liability and Insurance

The sponsor of this research adheres to the recommendations of the Association of British Pharmaceutical Industry Guidelines. If appropriate, a copy of the indemnity document is supplied to the investigator before study initiation, per local country guidelines.

The sponsor ensures that suitable clinical study insurance coverage is in place prior to the start of the study. An insurance certificate is supplied to the CRO/investigator as necessary.

10.1.3 Public Posting of Study Information

The sponsor is responsible for posting appropriate study information on applicable websites. Information included in clinical study registries may include participating investigators' names and contact information.

10.1.4 Submission of Summary of Clinical Study Report to Competent Authorities of Member States Concerned and Ethics Committees

The sponsor will provide a summary of the clinical study report to the competent authority of the member state(s) concerned as required by regulatory requirement(s) and to comply with the Community guideline on GCP. This requirement will be fulfilled within 6 months of the end of the study completion date for pediatric studies and within 1 year for non-pediatric studies as per guidance. The sponsor will provide the ECs with a copy of the same summary.

10.1.5 Study Suspension, Termination, and Completion

The sponsor may suspend or terminate the study, or part of the study, at any time for any reason. If the study is suspended or terminated, the sponsor will ensure that applicable sites, regulatory agencies, and IRBs/ECs are notified as appropriate. Additionally, the discontinuation of a registered clinical study which has been posted to a designated public website will be updated accordingly.

The sponsor will make an end-of-study declaration to the relevant competent authority as required by Article 10 (c) of Directive 2001/20/EC.

10.2 Investigator's Responsibilities

10.2.1 Good Clinical Practice Compliance

The investigator must undertake to perform the study in accordance with ICH GCP Guideline E6 (1996), EU Directive 2001/20/EC, and applicable regulatory requirements and guidelines.

It is the investigator's responsibility to ensure that adequate time and appropriately trained resources are available at the site prior to commitment to participate in this study. The investigator should also be able to estimate or demonstrate a potential for recruiting the required number of suitable subjects within the agreed recruitment period.

The investigator will maintain a list of appropriately qualified persons to whom the investigator has delegated significant study-related tasks, and shall, upon request of the sponsor, provide documented evidence of any licenses and certifications necessary to demonstrate such qualification. Curriculum vitae for investigators and sub-investigators are provided to the study sponsor (or designee) before starting the study.

If a potential research subject has a primary care physician, the investigator should, with the subject's consent, inform them of the subject's participation in the study.

A coordinating principal investigator is appointed to review the final clinical study report for multicenter studies. Agreement with the final clinical study report is documented by the signed and dated signature of the coordinating principal investigator, in compliance with Directive 2001/83/EC as amended by Directive 2003/63/EC and ICH Guidance E3 (1995).

10.2.2 Protocol Adherence and Investigator Agreement

The investigator and any co-investigators must adhere to the protocol as detailed in this document. The investigator is responsible for enrolling only those subjects who have met protocol eligibility criteria. Investigators are required to sign an investigator agreement to confirm acceptance and willingness to comply with the study protocol.

If the investigator suspends or terminates the study at their site, the investigator will promptly inform the sponsor and the IRB/EC and provide them with a detailed written explanation. The investigator will also return all investigational product, containers, and other study materials to the sponsor. Upon study completion, the investigator will provide the sponsor, IRB/EC, and regulatory agency with final reports and summaries as required by (inter)national regulations.

Communication with local IRBs/ECs, to ensure that accurate and timely information is provided at all phases during the study, may be done by the sponsor, applicable CRO, investigator, or for multicenter studies, the coordinating principal investigator according to national provisions and will be documented in the investigator agreement.

10.2.3 Documentation and Retention of Records

10.2.3.1 Electronic Case Report Forms

Electronic case report forms are supplied by the CRO and should be handled in accordance with instructions from the sponsor.

The investigator is responsible for maintaining adequate and accurate medical records from which accurate information is recorded into eCRFs, which have been designed to record all observations and other data pertinent to the clinical investigation. Electronic case report forms must be completed by the investigator or designee as stated in the site delegation log.

All data will have separate source documentation; no data will be recorded directly into the eCRF.

All data sent to the sponsor must be endorsed by the investigator.

The clinical research associate/study monitor will verify the contents against the source data per the monitoring plan. If the data are unclear or contradictory, queries are sent for corrections or verification of data.

10.2.3.2 Recording, Access, and Retention of Source Data and Study Documents

Original source data to be reviewed during this study will include, but are not limited to: subject's medical file including histology reports, subject-completed questionnaires and original clinical laboratory reports.

All key data must be recorded in the subject's medical records.

The investigator must permit authorized representatives of the sponsor, the respective national, local, or foreign regulatory authorities, the IRB/EC, and auditors to inspect facilities and to have direct access to original source records relevant to this study, regardless of media.

The clinical research associate/study monitor (and auditors, IRB/EC or regulatory inspectors) may check the eCRF entries against the source documents. The consent/assent form includes a statement by which the subject/LAR agrees to the monitor/auditor from the sponsor or its representatives, national or local regulatory authorities, or the IRB/EC, having access to source data (eg, subject's medical file, appointment books, original laboratory reports, X-rays etc). Non-study site personnel will not disclose any personal information or personal medical information.

These records must be made available within reasonable times for inspection and duplication, if required, by a properly authorized representative of any regulatory agency (eg, the US FDA, European Medicines Agency, United Kingdom Medicines and Healthcare products Regulatory Agency) or an auditor.

Essential documents must be maintained according to ICH GCP requirements and may not be destroyed without written permission from the sponsor.

10.2.3.3 Audit/Inspection

To ensure compliance with relevant regulations, data generated by this study must be available for inspection upon request by representatives of, for example, the US FDA (as well as other US national and local regulatory authorities), the European Medicines Agency, the United Kingdom Medicines and Healthcare products Regulatory Agency, other regulatory authorities, the sponsor or its representatives, and the IRB/EC for each site.

10.2.3.4 Financial Disclosure

The investigator is required to disclose any financial arrangement during the study and for 1 year after, whereby the outcome of the study could be influenced by the value of the compensation for conducting the study, or other payments the investigator received from the sponsor. The following information is collected: any significant payments from the sponsor or subsidiaries such as a grant to fund ongoing research, compensation in the form of equipment, retainer for ongoing consultation or honoraria; any proprietary interest in investigational product; any significant equity interest in the sponsor or subsidiaries as defined in Title 21 of the US Code of Federal Regulations Part 54 2(b) (1998).

10.3 Ethical Considerations

10.3.1 Informed Consent/Assent

It is the responsibility of the investigator to obtain written informed consent and assent where applicable from all study subjects prior to any study-related procedures including screening assessments.

All consent and assent documentation must be in accordance with applicable regulations and GCP. Each subject or the subject's LAR, as applicable, is requested to sign and date the subject informed consent/assent form or a certified translation if applicable, after the subject has received and read (or been read) the written subject information and received an explanation of what the study involves, including but not limited to: the objectives, potential benefits and risk, inconveniences, and the subject's rights and responsibilities. A copy of the informed consent and assent documentation (ie, a complete set of subject information sheets and fully executed signature pages) must be given to the subject or the subject's LAR, as applicable. This document may require translation into the local language. Signed consent/assent forms must remain in each subject's study file and must be available for verification at any time.

Within the source documents, site personnel should document instruction of and understanding by the parent/LAR/caregiver of the safe, responsible storage and administration of investigational product to the study subject.

The principal investigator provides the sponsor with a copy of the consent form and assent form where applicable which was reviewed by the IRB/EC and which received their favorable opinion/approval. A copy of the IRB/EC's written favorable opinion/approval of these documents must be provided to the sponsor, prior to the start of the study unless it is agreed to and documented (abiding by regulatory guidelines and national provisions) prior to study start that another party (ie, sponsor or coordinating principal investigator) is responsible for this action. Additionally, if the IRB/EC requires modification of the sample subject information and consent document provided by the sponsor, the documentation supporting this requirement must be provided to the sponsor.

10.3.2 Institutional Review Board or Ethics Committee

For sites outside the EU, it is the responsibility of the investigator to submit this protocol, the informed consent/assent document (approved by the sponsor or their designee), relevant supporting information and all types of subject recruitment information to the IRB/EC for review, and all must be approved prior to site initiation.

For sites within the EU, the applicant for an EC opinion can be the sponsor, the investigator, or for multicenter studies the coordinating principal investigator or sponsor, according to national provisions.

Responsibility for coordinating with IRBs/ECs is defined in the investigator agreement.

Prior to implementing changes in the study, the sponsor and the IRB/EC must approve any revisions of all informed consent/assent documents and amendments to the protocol unless there is a subject safety issue.

Investigational product supplies will not be released until the sponsor has received written IRB/EC approval of and copies of revised documents.

For sites outside the EU, the investigator is responsible for keeping the IRB/EC appraised of the progress of the study and of any changes made to the protocol, but in any case at least once a year; for sites within the EU, this can be done by the sponsor, the investigator or for multicenter studies the coordinating principal investigator, according to national provisions. The investigator must also keep the local IRB/EC informed of any serious and significant AEs.

10.4 Privacy and Confidentiality

All US-based sites and laboratories or entities providing support for this study, must, where applicable, comply with Health Insurance Portability and Accountability Act of 1996. A site that is not a covered entity as defined by Health Insurance Portability and Accountability Act must provide documentation of this fact to the CRO/sponsor.

The confidentiality of records that may be able to identify subjects will be protected in accordance with applicable laws, regulations, and guidelines.

After subjects have consented to take part in the study, the sponsor and/or its representative reviews their medical records and data collected during the study. These records and data may, in addition, be reviewed by others including the following: independent auditors who validate the data on behalf of the sponsor; third parties with whom the sponsor may develop, register, or market MMX Mesalamine/mesalazine; national or local regulatory authorities; and the IRBs/ECs which gave approval for the study to proceed. The sponsor and/or its representatives accessing the records and data will take all reasonable precautions in accordance with applicable laws, regulations, and guidelines to maintain the confidentiality of subjects' identities.

Subjects are assigned a unique identifying number; however, their initials and date of birth may also be collected and used to assist the sponsor to verify the accuracy of the data (eg, to confirm that laboratory results have been assigned to the correct subject).

The results of studies – containing subjects' unique identifying number, relevant medical records, and possibly initials and dates of birth – will be recorded. They may be transferred to, and used in, other countries which may not afford the same level of protection that applies within the countries where this study is conducted. The purpose of any such transfer would include: to support regulatory submissions, to conduct new data analyses to publish or present the study results, or to answer questions asked by regulatory or health authorities.

10.5 Study Results/Publication Policy

Shire will endeavor to publish the results of all qualifying, applicable, and covered studies according to external guidelines in a timely manner regardless of whether the outcomes are perceived as positive, neutral, or negative. Additionally, Shire adheres to external guidelines (eg, Good Publication Practices 2) when forming a publication steering committee, which is done for large, multicenter Phase 2-4 and certain other studies as determined by Shire. The purpose of the publication steering committee is to act as a non-commercial body that advises or decides on dissemination of scientific study data in accordance with the scope of this policy.

All publications relating to Shire products or projects must undergo appropriate technical and intellectual property review, with Shire agreement to publish prior to release of information. The review is aimed at protecting the sponsor's proprietary information existing either at the commencement of the study or generated during the study. To the extent permitted by the publisher and copyright law, the principal investigator will own (or share with other authors) the copyright on his/her publications. To the extent that the principal investigator has such sole, joint or shared rights, the principal investigator grants the sponsor a perpetual, irrevocable, royalty-free license to make and distribute copies of such publications.

The term "publication" refers to any public disclosure including original research articles, review articles, oral presentations, abstracts, and posters at medical congresses, journal supplements, letters to the editor, invited lectures, opinion pieces, book chapters, electronic postings on medical/scientific websites, or other disclosure of the study results, in printed, electronic, oral or other form.

Subject to the terms of the paragraph below, the investigator shall have the right to publish the study results, and any background information provided by the sponsor that is necessary to include in any publication of study results, or necessary for other scholars to verify such study results. Notwithstanding the foregoing, no publication that incorporates the sponsor's confidential information shall be submitted for publication without the sponsor's prior written agreement to publish, and shall be given to the sponsor for review at least 60 days prior to submission for publication. If requested in writing by Shire, the institution and principal investigator shall withhold submission of such publication for up to an additional 60 days to allow for filing of a patent application.

If the study is part of a multicenter study, the first publication of the study results shall be made by the sponsor in conjunction with the sponsor's presentation of a joint, multicenter publication of the compiled and analyzed study results. If such a multicenter publication is not submitted to a journal for publication by the sponsor within an 18-month period after conclusion, abandonment, or termination of the study at all sites, or after the sponsor confirms there shall be no multicenter study publication of the study results, an investigator may individually publish the study results from the specific site in accordance with this section. The investigator must, however, acknowledge in the publication the limitations of the single-site data being presented.

Unless otherwise required by the journal in which the publication appears, or the forum in which it is made, authorship will comply with the International Committee of Medical Journal Editors current standards. Participation as an investigator does not confer any rights to authorship of publications.

11. REFERENCES

Mendeloff AI, Calkins BM 1988. The epidemiology of idiopathic inflammatory bowel disease. In: Kirsner, JB, Shorter, RG (Eds), *Inflammatory Bowel Disease*. Lea Febiger, Philadelphia.

Otley A, Smith C, Nicholas D, Munk M, Avolio J, Sherman PM, et al. 2002. The IMPACT Questionnaire: A valid measure of health-related quality of life in pediatric inflammatory bowel disease. *J Pediatr Gastroenterol Nutr*; 35(4): 557-63.

Turner D, Otley A, Mack D, Hyams J, De Bruijne J, Uusoue K, et al. 2007. Development, validation, and evaluation of a pediatric ulcerative colitis activity index: a prospective multicenter study. *Gastroenterology*; 133: 423-32.

Turner D, Hyams J, Markowitz J, Lerer T, Mack DR, Evans J, et al. 2009. Appraisal of the Pediatric Ulcerative Colitis Activity Index (PUCAI). *Inflamm Bowel Dis*; 15(8): 1218-23.

Turner D, Levine A, Escher JC, Griffiths AM, Russell RK, Dignass A, et al. 2012. Management of pediatric ulcerative colitis: Joint ECCO and ESPGHAN evidence-based consensus guidelines. *J Pediatr Gastroenterol Nutr*; 55(3): 340-61.

Schroeder KW, Tremaine WJ, Ilstrup DM 1987. Coated oral 5-aminosalicylic acid therapy for mildly to moderately active ulcerative colitis. A randomized study. *N Engl J Med*; 317(26): 1625-9.

Sutherland LR, Martin F, Greer S, Robinson M, Greenberger N, Saibil F, et al. 1987. 5-Aminosalicylic acid enema in the treatment of distal ulcerative colitis, proctosigmoiditis, and proctitis. *Gastroenterology*; 92(6): 1894-8.

12. APPENDICES

APPENDIX 1 PROTOCOL HISTORY

Document	Date	Global/Country/Site Specific
Original Protocol	11 Sep 2013	Global
Amendment 1	17 Dec 2013	Global
Amendment 2	14 Jul 2014	Global
Amendment 3	09 Feb 2015	Global
Amendment 4	28 Nov 2016	Global
Amendment 5	10 Apr 2017	Global

Protocol Amendments		
Summary of Change(s) Since Last Version of Approved Protocol		
**Amendment Number**<br>**4**	**Amendment Date**<br>**28 Nov 2016**	**Global/Country/Site Specific**<br>**Global**
**Description of Change**		**Section(s) Affected by Change**
Minor copyediting performed for consistency in style and presentation of the document.		Global
Updated sponsor's address		Title Page, Product Quality Complaints Page
Updated sponsor's approval signatory.		Protocol Signature Page
Updated non-emergency e-mail contact information for CRO medical monitors.		Emergency Contact Information
Added abbreviation for Cochran-Mantel-Haenszel		Abbreviations
Updated approximate number of subjects to be screened.<br>**Rationale: To reflect the fact that based on actual screening numbers, it is anticipated that more subjects will need to be screened to achieve 160 subjects in total (80 subjects in the Double-blind Acute Phase and 80 subjects in the Double-blind Maintenance Phase).**		Synopsis, Section 3.1
Added text indicating that enrollment in the Double-blind Maintenance Phase will be considered complete once 80 subjects have been randomized into this phase.<br>**Rationale: Capping enrollment in the Double-blind Maintenance Phase will allow for extension of the recruitment period for the Double-blind Acute Phase. Subjects will continue to roll over into the Double-blind maintenance phase; however, the data collected from these additional subjects will be analyzed in an addendum to the clinical study report.**		Synopsis, Section 3.1
Increased maximum duration of screening period from 14 to 21 days.<br>**Rationale: To allow sites additional time to complete screening assessments prior to enrollment.**		Synopsis, Table 1, Table 3, Section 3.1, Section 5.3, Section 7.1.1.1, Section 7.1.1.3, Section 7.2.2.8

Protocol Amendments		
**Summary of Change(s) Since Last Version of Approved Protocol**		
**Amendment Number**<br>**4**	**Amendment Date**<br>**28 Nov 2016**	**Global/Country/Site Specific**<br>**Global**
**Description of Change**		**Section(s) Affected by Change**
Clarified eligibility criteria for Double-blind Acute Phase and Double-blind Maintenance Phase to indicate that all 3 components of the UC-DAI score are required as well as assessment of the mucosal appearance (endoscopy score).<br>**Rationale: To clarify eligibility criteria and ensure consistency of these criteria throughout the protocol.**		Synopsis, Table 1, Section 4.1, Section 7.1.1.3, Section 7.2.2.8
Added text as follows:<br>• Subjects with partial UC-DAI score between 1 and 2 will not be eligible to enter the Double-blind Acute Phase or the Double-blind Maintenance Phase.<br>• Subjects who do not meet all the eligibility criteria for either the Double-blind Acute Phase or the Double-blind Maintenance Phase will be considered screen failures.<br>• A minimum of 1 score total for stool frequency and rectal bleeding must be available in order to randomize subjects into either the Double-blind Acute Phase or the Double-blind Maintenance Phase (see Section 7.2.1.1 for details). Subjects with no symptom score data available will be considered screen failures as these symptom score data are used to calculate the partial UC-DAI score needed for enrollment.<br>**Rationale: To clarify eligibility criteria for Double-blind Acute and Double-blind Maintenance Phases and to emphasize that subjects who do not meet these criteria will be considered screen failures.**		Synopsis, Section 3.1, Section 7.1.1.3
Generated new study design flow chart to increase clarity on determination of eligibility for entry into each of the 3 treatment phases.		Section 3.1
Updated Double-blind Acute Phase Inclusion criterion number 8 to clarify mucosal appearance (endoscopy score) at Baseline Visit as "mucosal appearance (endoscopy score)=2 or 3" (previously defined as "mucosal appearance [endoscopy score] >1")		Synopsis, Section 4.1, Section 7.1.1.3, Figure 1
Updated Double-blind Acute Phase Exclusion criterion number 27 to clarify mucosal appearance and endoscopic score definition as, "Mucosal appearance (defined by endoscopic score=0 or 1)" (previously defined as "Normal mucosal appearance [defined by endoscopic score=0]")		Synopsis, Section 4.2
Updated Double-blind Maintenance Phase Inclusion criterion number 10 to clarify mucosal appearance (endoscopy score) at Baseline Visit as "mucosal appearance (endoscopy score)=0 or 1" (previously defined as "mucosal appearance [endoscopy score] <1")		Synopsis, Study Schedule (Table 1), Section 3.1, Section 4.1, Section 7.1.1.3, Figure 1

Protocol Amendments		
**Summary of Change(s) Since Last Version of Approved Protocol**		
**Amendment Number**<br>**4**	**Amendment Date**<br>**28 Nov 2016**	**Global/Country/Site Specific**<br>**Global**
**Description of Change**		**Section(s) Affected by Change**
Updated Exclusion criteria as follows:<br>• Removed exclusion of UC confined to the rectum<br>**Rationale: Removed as proctitis is an unusual type of UC in children that, in some cases, does not differ from regular UC either clinically or histologically.**<br>• Clarified hepatic impairment exclusion<br>**Rationale: Clarified as UC is usually paired with primary sclerosing cholangitis (PSC) and mild to moderate PSC is often treated with mesalazine. The revised text offers some flexibility around these cases.**<br>• Updated criterion regarding corticosteroid use to indicate that concomitant rectal corticosteroid use will be allowed following randomization<br>**Rationale: Modified to indicate that concomitant rectal corticosteroid use will be allowed following randomization to align with the widely used standard of care that is outlined in the Joint European Crohn's and Colitis Organisation and European Society for Paediatric Gastroenterology, Hepatology, and Nutrition guidelines.**<br>• Removed exclusion of prebiotic/probiotic use<br>**Rationale: Removed this restriction as prebiotic/probiotic use is common in patients with mild UC and excluding their use would negatively impact recruitment.**<br>• Clarified criterion that excluded subjects previously screened or randomized into this study. Previously screened subjects will be eligible and previously randomized subjects will not be eligible<br>**Rationale: To date, many screen failures for this study can be attributed to technical reasons (ie, late arrival of laboratory results or biopsy, delay in endoscopy, etc). In addition, as the UC status of subjects change, some subjects who may not have qualified for the Double-blind Acute Phase might subsequently qualify for the Double-blind Maintenance Phase. Allowing rescreening of screen failures increases the pool of eligible subjects who may benefit from this study.**<br>• Updated definition of current use of medication to reflect extended screening period<br>**Rationale: Updated window for current medication use to reflect extended screening period; screening period was extended to allow additional time for performing endoscopies and obtaining histology.**		Synopsis, Section 4.2, Section 5.3, Section 7.1.1.1

Protocol Amendments		
**Summary of Change(s) Since Last Version of Approved Protocol**		
**Amendment Number**<br>**4**	**Amendment Date**<br>**28 Nov 2016**	**Global/Country/Site Specific**<br>**Global**
**Description of Change**		**Section(s) Affected by Change**
Removed the model-based imputation analysis from the sensitivity analyses of the primary endpoint.<br>**Rationale: Removed this analysis as this study is not powered to detect differences between treatment groups. Additionally, other sensitivity analyses (ie, last observation carried forward and complete case) intended to examine the robustness of the treatment estimate (under the assumption that the subjects with missing Week 8 or Week 26 results are treatment failures) have been included.**		Synopsis, Section 9.8.2
Added a column and a row to the Schedule of Assessments to increase clarity and awareness for sites that it is recommended that site staff telephone subjects within 4 to 7 days prior to the Baseline Visit and prior to each site visit to remind subject or the subject's caregiver to enter their UC-DAI symptoms (rectal bleeding and stool frequency) into their e-diaries every night. This was previously only mentioned in Sections 7.1 and 7.2.		Schedule of Assessments (Table 1, Table 2, Table 3)
Added text describing the recommended telephone call to be performed by site staff within 4 to 7 days prior to the Baseline Visit and prior to each site visit to remind subject or the subject's caregiver to enter their UC-DAI symptoms (rectal bleeding and stool frequency) into their e-diaries every night.		Section 7.1.1.2, Section 7.1.2.1, Section 7.1.3.1, Section 7.1.4.1
Added a footnote indicating that subjects may bring in a standard of care stool sample (if collected within 24 hours) for screening assessments and evaluations.		Schedule of Assessments (Table 1, Table 2, Table 3)
Added text indicating that stool samples collected for culture may be processed by a local laboratory and that any subjects with positive stool cultures will not be eligible for the study.		Section 7.1.1.1, Section 7.2.2.8
Updated assessment time points in schedule of assessments for Double-blind Acute Phase, Open-label Acute Phase, and Double-blind Maintenance Phase.		Schedule of Assessments (Table 1, Table 2, Table 3)
Clarified that subjects who do not meet all the eligibility criteria for either the Double-blind Acute Phase or the Double-blind Maintenance Phase, and who are considered screen failures, may be rescreened after consultation with the medical monitor.		Synopsis, Section 3.1, Section 7.1.1.1, Section 7.1.1.3
Updated site-specific labeling requirements in line with the Administrative Change #3 memo dated 02 Jul 2015.		Section 6.3.1

Protocol Amendments		
**Summary of Change(s) Since Last Version of Approved Protocol**		
**Amendment Number**<br>**3**	**Amendment Date**<br>**09 Feb 2015**	**Global/Country/Site Specific**<br>**Global**
**Description of Change**		**Section(s) Affected by Change**
Updated sponsor's approval signatory.		Protocol Signature Page
Added Shire and CRO contact information for SAE reporting.		Emergency Contact Information
Added non-emergency e-mail contact information for CRO medical monitors.		Emergency Contact Information
Added abbreviation for Ac-5-ASA.		Abbreviations
Increased maximum duration of screening period from 10 to 14 days.		Synopsis, Table 1, Table 3, Section 7.1.1.1, Section 7.1.1.2, Section 7.2.2.8
Added an exception to exclusion criteria #21 regarding re-screening of subjects.		Synopsis, Section 4.2
Updated approximate number of study sites from 43 to 48.		Synopsis, Section 3.3
Added pharmacokinetic blood sampling.		Table 1, Table 2, Table 3, Section 7.1.2.2, Section 7.1.3.2 Section 7.1.4.3
Added urinalysis assessment.		Table 1, Table 2, Table 3
Clarification added regarding the ability to perform a second endoscopy during the period of the Screening Visit (Visit 1). This is applicable when eligibility cannot be determined from the initial endoscopy.		Table 1, Table 3, Section 7.1.1.1
Added text to allow up to additional 14 days after eligibility endoscopy for repeat endoscopy for subjects with inadequate bowel preparation.		Table 1, Table 3, Section 7.1.1.1
Added exploratory objective for pharmacokinetic assessment.		Section 2.2.3
Updated approximate number of countries participating in the study from 9 to 8.		Section 3.3
Added text to describe the situation where a subject may be re-screened.		Section 7.1.1.1
Added text to allow for a stool sample obtained per standard of care within 24 hours prior to the Screening Visit (Visit 1), to be used for screening stool assessments.		Section 7.1.1.1, Section 7.2.2.8
Clarification of additional care of subjects added.		Section 7.1.6
[redacted]		Section 7.2.2.8
Added section to describe pharmacokinetic blood sampling collection, handling, shipping and assay methodology.		Section 7.2.3
Updated contact information for reporting of SAEs to the CRO.		Section 8.2.2
Clarified subject status included for DMC review.		Section 9.5
Added exploratory endpoint for pharmacokinetic assessment.		Section 9.8.5

Summary of Change(s) Since Last Version of Approved Protocol		
**Amendment Number**<br>**2**	**Amendment Date**<br>**14 Jul 2014**	**Global/Country/Site Specific**<br>**Global**
**Description of Change**		**Section(s) Affected by Change**
Updated United States Premier Research Medical Monitor.		Emergency Contact Information
Data Monitoring Committee added.		Abbreviations, Section 9.5
Corrected definition of Clinical Response by removing "Week 8".		Definitions
"Continuity-corrected" chi-squared test was added to the method to test the primary/secondary endpoints.		Synopsis, Section 9.8.3.1
Revised number of active study sites from 35 to 43		Synopsis, Section 3.3
Added comment to footnotes to clarify that final visit of Double-blind Acute Phase and of the Open-label Acute Phase will be used as Week 0 for the subject's next phase of participation.		Table 1, Table 2
Clarified footnote regarding endoscopy requirements for subjects entering the Double-blind Maintenance Phase directly.		Table 3
Added height assessments.		Table 1, Table 2, Table 3
Text amended to allow for data from an endoscopy performed within 7 days prior to the Screening Visit (Visit 1) to be used in place of an endoscopy performed during the Screening Visit (Visit 1) or Baseline Visit (Visit 2) for subjects entering the Double-blind Acute Phase.		Table 1, Table 3, Section 7.1.1.1, Section 7.1.1.2, Section 7.2.1.2
Removed height from physical examination.		Section 7.2.2.2
Added IRT Entry to schedule of assessments.		Table 1, Table 2, Table 3
Added text to indicate that PUCAI will not be collected by all study sites.		Table 1, Table 2, Table 3, Section 7.1.1.2, Section 7.1.2.2, Section 7.1.3.2, Section 7.1.4.3, Section 7.2.3.1
Text related to description of subjects who may have an endoscopy removed for clarity.		Table 2
Added weight assessment to the end of the Double-blind Maintenance Phase.		Table 3, Section 7.2.2.3
Removed blank column for 90 days.		Table 5
Revised information to describe the rationale for the study design.		Section 2.1
Added clarification that an additional endoscopy is not required for subjects enrolling into the Double-blind Maintenance Phase from the Double-blind Acute Phase or the Open-label Acute Phase.		Synopsis, Section 3.1, Section 4.3
Corrected visit number.		Section 4.5
Information regarding the provision of additional care after the study included.		Section 7.1.6
Clarified entry requirements for Double-blind Maintenance Phase by adding endoscopy score component.		Section 3.1, Section 7.1.1.2
Added section to describe standardization of height and weight measurements.		Section 7.2.2.3
Deleted duplicate entry for total bilirubin.		Section 7.2.2.6
Clarified naming of laboratory assessments.		Section 7.2.2.6

Summary of Change(s) Since Last Version of Approved Protocol		
**Amendment Number**<br>**2**	**Amendment Date**<br>**14 Jul 2014**	**Global/Country/Site Specific**<br>**Global**
**Description of Change**		**Section(s) Affected by Change**
Added reference to Section 8.1.5 for potential additional laboratory assessments in the case of elevated liver enzymes.		Section 7.2.2.6
Clarification made to requirement for stool sample collection prior to administration of bowel preparation medications.		Section 7.2.2.8
Detail added to clarify the baseline timepoint for each study phase.		Section 9.8, Section 9.9
Corrected reference to buccal swab from blood sample.		Appendix 5

**Amendment Number**<br>**1**	**Amendment Date**<br>**17 Dec 2013**	**Global/Country/Site Specific**<br>**Global**
**Description of Change**		**Section(s) Affected by Change**
Added second principal coordinating investigator.		Cover Page
Added visit (Weeks 2-4) to Double-blind Maintenance Phase for drug dispensing purposes; revised subsequent visit numbers.		Synopsis, Table 3, Section 7.1.1.3, Section 7.1.4.1, Section 7.1.4.2, Section 7.1.4.3, Section 7.2.1.1, Section 7.2.2.6
Corrected terminology for subject disease state in the primary and secondary objectives for the Double-blind Maintenance Phase from "with mild to moderate UC" to "who are in remission."		Synopsis, Section 2.2.1, Section 2.2.2
Removed normal mucosal appearance criterion from "general" exclusion criteria; added to the individual exclusion criteria of the Double-blind Acute Phase. Created individual exclusion criterion for the Double-blind Maintenance Phase.		Synopsis, Section 4.2
Specified in re-randomization criteria that subjects may be re-randomized into the Double-blind Maintenance Phase if they turned 18 during participation in either Acute Phase of the study.		Synopsis, Section 4.3
Removed text regarding sensitivity analysis of the primary endpoint from "Double Blind Maintenance Phase" section of the protocol in Section 9.8. Also removed text from Synopsis regarding sensitivity analysis of the primary endpoint. Replaced with the newly created Sensitivity Analyses of the Primary Endpoint section.		Synopsis, Section 9.8.1.2, Section 9.8.2
Added detail specifying how primary and secondary efficacy endpoints will be described and compared in Double-blind Acute Phases, Double-blind Maintenance Phases, and overall.		Synopsis, Section 9.8.3, Section 9.8.3.1, Section 9.8.3.2
Changed Double-blind Maintenance Phase primary efficacy endpoint test from chi-squared test to Mantel-Haenszel test with stratification by responder details.		Synopsis, Section 9.8.1.2, Section 9.8.3.2
Included Physician's Global Assessment at Week 2 and Week 4 of the Double-blind Acute Phase.		Table 1, Section 7.1.2.1

Amendment Number<br>1	Amendment Date<br>17 Dec 2013	Global/Country/Site Specific<br>Global
**Description of Change**		**Section(s) Affected by Change**
Corrected ages for questions and responses for rectal bleeding and stool frequency in e-diary for both versions: children and adolescents (ages 11-17 years) and caregivers of children (children aged 5-10 years). Specified version of e-diary to be utilized in the case of a change in subject age during the study. Also updated age groups for Global Change in Health questionnaire.		Table 1, Table 3, Section 7.2.1.1, Section 7.2.3
Made correction to currently available pediatric ulcerative colitis treatment.		Section 1.1
Added site evaluation procedure to minimize missing data in the study.		Section 4.5.2
Clarified stable dose exception (fish oil) for prohibited medication.		Section 5.3
Corrected open-label bottle size from 75cc to 100cc.		Section 6.3.2
Clarified period of Screening Visit (Visit 1).		Section 7.1.1.1, Section 7.1.1.2 Section 7.2.2.7
Specified video as the preferred method for provision of endoscopic images to central reader for evaluation.		Section 7.1.1.1, Section 7.1.2.2, Section 7.1.3.2, Section 7.1.4.3, Section 7.2.1.2
Added intention to collect results of endoscopies obtained as a part of routine clinical care during a subject's participation in the study.		Section 7.2.1.2
Added text to specify that ulcerative colitis history will be collected.		Section 7.2.2.1
Added requirement to report serious adverse events to the CRO Medical Monitor.		Section 8.2.2
Removed double-blind Acute Phase Full Analysis Set and Double-blind Maintenance Phase Full Analysis Set.		Section 9.7
Full Analysis sets have been changed to the Safety Analysis sets.		Section 9.8.1, Section 9.8.1.1, Section 9.8.1.2, Section 9.8.3

APPENDIX 2 THE MODIFIED FULL ULCERATIVE COLITIS DISEASE ACTIVITY INDEX (UC-DAI)

The modified full UC-DAI Score is computed as the sum of scores in 4 categories of clinical symptoms: stool frequency, rectal bleeding, and mucosal appearance (endoscopy score) and PGA.

The 4 components of the modified full UC-DAI are scored in the following way:

Stool frequency Score

Normal	0
1-2 more than normal/day	1
3-4 more than normal/day	2
>4 more than normal/day	3

Rectal bleeding (most severe bleeding of the day)

None	0
Streaks of blood	1
Obvious blood	2
Mostly blood	3

Mucosal appearance (endoscopy score)

Normal (intact vascular pattern; no friability or granulation)	0
Mild (erythema, decreased vascular pattern, minimal granularity)	1
Moderate (marked erythema, granularity, friability, absent vascular pattern, bleeding with minimal trauma, no ulcerations)	2
Severe (ulceration, spontaneous bleeding)	3

Physician's Global Assessment

No active disease	0
Mild disease	1
Moderate disease	2
Severe disease	3

APPENDIX 3 SUBJECT QUESTIONNAIRES

The following subject completed questionnaires will be utilized in this study:

Daily Ulcerative Colitis Scale for Children and Caregivers

Overall Current Health

Rectal Bleeding

Stool Frequency

Global Change in Health

IMPACT III

A separate master file containing each subject questionnaire listed above will be provided to the site. Updates to subject questionnaires during the study (if applicable) will be documented in the table above and a new master file containing the revised subject questionnaire(s) will be provided to the site.

APPENDIX 4 PEDIATRIC ULCERATIVE COLITIS ACTIVITY INDEX

Item	Points
1. Abdominal pain	
No pain	0
Pain can be ignored	5
Pain cannot be ignored	10
2. Rectal bleeding	
None	0
Small amount only, in less than 50% of stools	10
Small amount with most stools	20
Large amount (>50% of the stool content)	30
3. Stool consistency of most stools	
Formed	0
Partially formed	5
Completely unformed	10
4. Number of stools per 24 hours	
0–2	0
3–5	5
6–8	10
>8	15
5. Nocturnal stools (any episode causing wakening)	
No	0
Yes	10
6. Activity level	
No limitation of activity	0
Occasional limitation of activity	5
Severe restricted activity	10
Sum of PUCAI (0–85)	

© Copyright to The Hospital for Sick Children, Toronto, Canada, 2006.

[redacted]

[redacted]

[redacted]

[redacted]
